# Reconsidering how we define quality: insights from the co-development of an actionable definition of oral healthcare quality

**DOI:** 10.1186/s12961-026-01536-8

**Published:** 2026-09-11

**Authors:** Michael Lorenz, Ziade Sarroukh, Stefan Listl

**Affiliations:** 1 Section for Oral Health, Heidelberg Institute of Global Health, Heidelberg University Hospital, Medical Faculty Heidelberg, Heidelberg University, Heidelberg, Germany; 2 Department of Dentistry, Quality and Safety of Oral Health Care, Radboud University Medical Center, Nijmegen, The Netherlands

**Keywords:** Oral health, Quality of care, Definition, Co-development, Deliberative methods, Europe

## Abstract

**Background:**

The burden of oral diseases on society remains relevant as vulnerable groups lack accessibility to oral healthcare and it is expensive on a societal scale. While there is renewed political will from the UN to expand Universal Health Coverage to oral health, quality issues remain. Solving such complex systematic problems requires coherent efforts that span multiple stakeholders and sectors. The co-development of a definition of oral healthcare quality serves as a key enabler for quality improvement interventions as it facilitates collective understanding and normative directionality.

**Methods:**

As part of the EU DELIVER project, a five-step co-development process was leveraged for consenting an actionable definition for quality of oral healthcare: (1) literature screening to identify relevant themes of quality of oral healthcare; (2) compilation of a starting list from these themes; (3) World Café sessions to deliberate about themes to define quality of oral healthcare; (4) thematic analysis of information from the World Café sessions; (5) final voting and completion of the definition. The definition provides a clear foundation and normative directionality for co-developing quality indicators and targeted interventions for quality improvement. The Consolidated criteria for reporting qualitative studies (COREQ-32) were used to report the study.

**Results:**

Drawing from the breadth of the extant scientific literature and the depth of stakeholder insights from the World Café sessions, the following working definition was agreed upon: "Quality oral healthcare refers to the contribution of healthcare to optimal oral health. This entails that care alleviates oral health inequity, is comprehensive, empowers patients, mitigates harm and encourages learning systematically, improves well-being, minimizes waste of resources and is provided at the right time. To maximize quality, care should be equitable, accessible, patient-centered, safe, effective, efficient and timely." The definition forms the basis for the operationalization of targeted quality improvement interventions on the practice-, community- and health systems levels.

**Conclusions:**

The definition for quality of oral healthcare is the first that was co-developed together with providers, policy-makers, and patients from various European countries. This definition provides meaningful normative direction and is highly relevant to collective problem-solving aimed at optimizing the quality of oral healthcare.

**Supplementary Information:**

The online version contains supplementary material available at https://doi.org/10.1186/s12961-026-01536-8.

## Background

The global burden of oral diseases has remained persistently high over the past 30 years, with nearly half the world's population affected, and this burden is disproportionately concentrated among vulnerable groups [1, 2]. Within the European Union (EU), oral conditions are the third most expensive diseases to treat [3] and have been shown to be a main driver of catastrophic health expenditures [4, 5]. Healthcare systems in the EU have thus far failed to uphold the goals of Universal Health Coverage (UHC) set by the UN and WHO [6], with many EU citizens lacking access to quality oral healthcare without financial hardship, particularly among poor and marginalized groups [5, 7].

Despite the multilateral damage to society, a low sense of urgency for quality improvement persists in policy and practice. For those who do not have access to affordable oral care, this poses serious safety threats as the exacerbation of oral diseases can lead to infections, emergency hospital admissions, or even death [8]. For those who can access and afford oral health care, recent evidence raises serious concerns about adverse effects due to diagnostic and therapeutic errors, such as overuse of dental X-raying [9, 10], limited materiovigilance [11], and medication lapses such as antibiotic overprescribing [12] and concerns regarding antimicrobial resistance [13]. Additionally, evidence suggests distrust of patients toward their providers and oral healthcare being perceived as a poor value for the money [14]. These multifaceted challenges highlight the disconnect between different stakeholders and the need for quality improvement through consented definitions and measures [15–17].

The incapacity of current health systems to alleviate the oral disease burden [1] proves that there is a serious lack of synergistic problem-solving involving citizens, patients, providers, and policy-makers. Nevertheless, solving such complex systematic problems requires coherent quality improvement strategies and efforts that span multiple stakeholders and sectors. So far, there is an enormous dearth of comprehensive quality improvement initiatives in the oral health context [18–21]. Oral health systems largely operate in isolation from the rest of medicine [22], creating an enclosed ecosystem with idiosyncratic and intricate challenges for oral care quality improvement.

To this end, the EU DELIVER (DELiberative ImproVEment of oRal care quality) project aims to enhance the quality of oral healthcare through deliberative dialogue and action involving citizens, patients, providers, payers, and policy-makers. DELIVER strives to create a synergistic problem-solving ecosystem to convert deliberative dialogues into meaningful improvement of oral care quality. The co-development of a mutual understanding of what constitutes quality of oral healthcare was identified as a essential requirement to facilitate collective understanding and normative directionality. The purpose of this paper is to describe the development of the DELIVER definition for quality of oral healthcare, which serves as a fundamental enabler for quality improvement interventions and as a basis for all subsequent activities within the DELIVER project. The key objectives of the present study are therefore the co-development of a definition of oral healthcare quality with contextual relevance across several European countries and the description of a co-development process applicable to other contexts.

## Methods

**Fig. 1:**
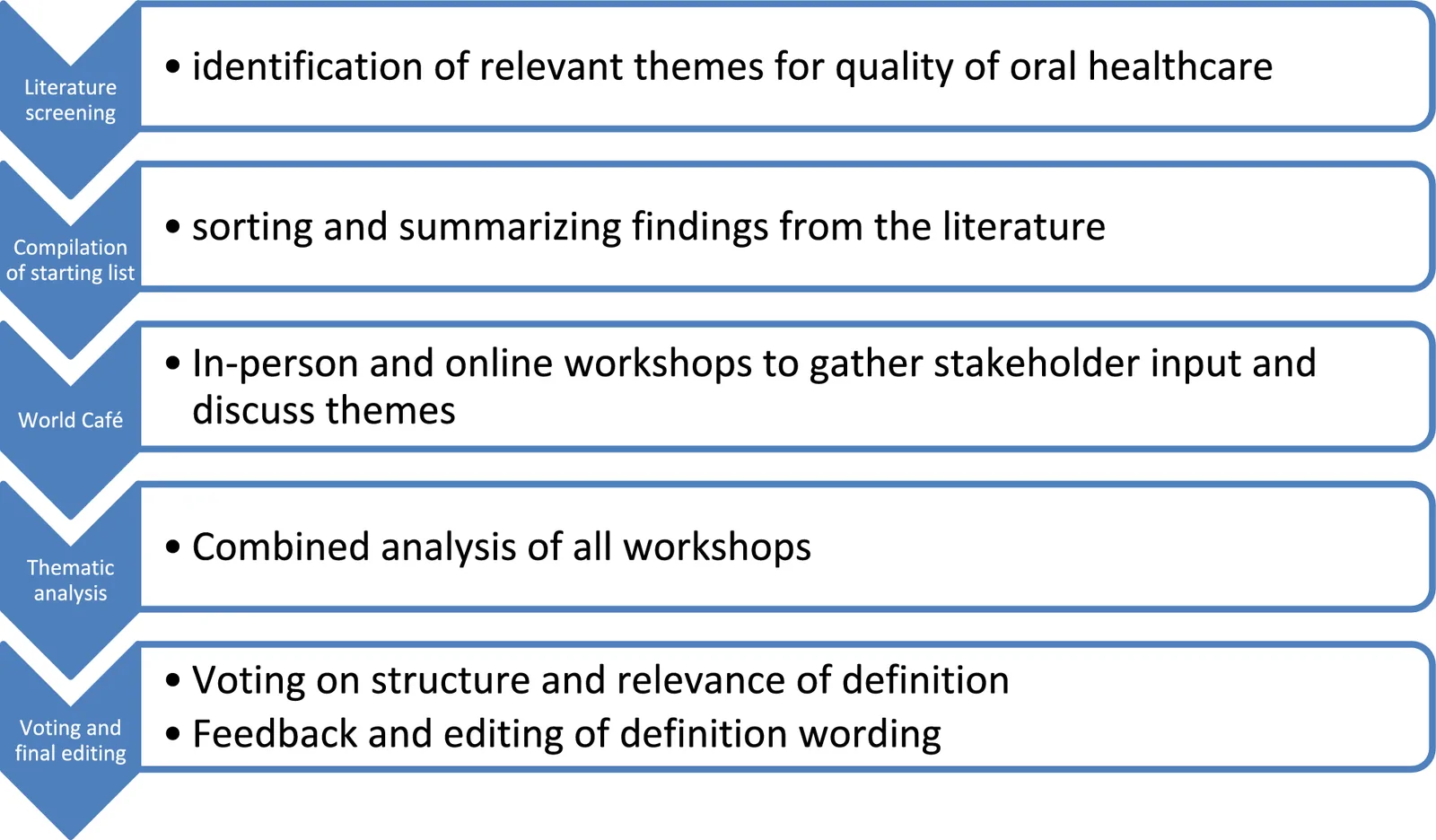
The five-step co-development process

A five-step co-development process was used to develop a definition of quality oral healthcare; see Fig. 1. Reporting of this study follows the COREQ-32 criteria [23], and further details can be found in Annex 1 where all items on the COREQ-32 checklist are addressed.

The overall framework of the definition is based on the Institute of Medicine (IOM) definition of healthcare quality, which includes patient safety, effectiveness, patient-centredness, timeliness, efficiency, and equitability as domains [24] and its adaptation by Righolt et al., which notably added accessibility as a domain of its own as this was perceived to be highly important for oral healthcare specifically [25].

### Literature screening

Building on previous work by Righolt et al. [25], an updated literature review was conducted to include new relevant literature and literature specific to the domain of accessibility. Papers discussing the quality of oral healthcare were searched using MeSH Terms in PubMed and included if published in 2018 or later, as the search by Righolt et al. was done in December 2017 [25]. If the scope and theme of a paper were clear from the abstract, no full-text screening took place, and only the themes emergent from the abstract were included. This streamlining was deemed appropriate as the focus was not a deep literature review but rather familiarization with the vast topic list relevant to oral healthcare quality. Searches were carried out between 19 September 2022 and 30 September 2022, and the screening of search results was conducted between 19 September 2022 and 31 October 2022 by two researchers (ML, ZS). The search only included publications in which the abstract was available in English, German, or French; German or French content was freely translated by one of the authors (ML) to capture the key concepts and topics. Further details including the search strategy and search results are given in Annex 2: Overview literature review, search strategy and search results.

### Initial topic list

The results of the literature review complemented the list of topics described by a previous review [25]. Two researchers screened the publications and assigned the topics identified there to overarching themes. Remaining uncertainties were resolved by consensus among the reviewers. This topic list served two purposes: it familiarized the researchers with the breadth of topics that could be relevant, and it was designed to be used as a tool for the moderators of the following World Café workshops to initiate and sustain discussions among stakeholders if needed. There was no calculation of inter-reviewer reliability and no duplicate reviewing; this was deemed justifiable as it was estimated that the literature screening could achieve the stated goals more efficiently this way.

### World Café

The World Café is a method for engaging a group designed to spur dynamic discussions, gather input from all participants, and generate new ideas [26]. Workshops followed a consistent structure: First, a general overview of the study and methodology was given, including repeating study information, data protection information, and consent as well as rules to allow for open discussion, such as confidentiality. Next, the participants were split into smaller groups and joined by a moderator. Each group had 10 min per domain to discuss what this domain would mean for them, whether and how it was relevant to them, and whatever else came to mind when thinking about this subject and hearing from the other participants. The moderators’ role was limited to delivering short summaries of what the previous group(s) had discussed in this domain, and, if needed, exposing participants to some quality themes that emerged in the literature screening to get the discussion going. During the workshops, the moderators’ task was also to document the group discussion on flipchart paper or a digital whiteboard, with all participants able to see what was written, and the moderator actively checking with the group to identify the intended wording. If there were disagreements, the moderator facilitated the discussion until consensus within the group was reached. After the workshops, the moderators discussed the session and took additional notes. All workshops were held in English. The first of these workshops was held during DELIVER’s first in-person consortium meeting in November 2022 (Porto, Portugal), where seven groups were formed and the moderators moved from table to table. Next, online sessions were organized in December 2022 and January 2023 to allow for broader participation of other stakeholders. These sessions had fewer participants, so fewer or no subgroups were formed. Each stakeholder group was assigned special workshops as this homogeneity allowed participants to discuss topics comfortably among their peers and in the same “language”. The notes from the World Café then were the basis for the next step.

### Thematic analysis

Inductive and deductive coding of input from all World Café sessions was conducted, applying thematic analysis. For deductive coding, the starting list developed from the literature screening served as a point of reference. Regarding decision-making during coding, the aim was to develop codes that represent raised topics closely to preserve the variety of raised topics. This variety was then used to check whether the suggested domains could be used to structure the topics. As the stakeholder topics were already sorted into the different domains within the workshop, the construction of themes outside of these topics also was aligned with this structure. However, there were some cases where constructed themes could potentially fit multiple domains, in which case the main aspect was elicited in discussion between the researchers and in consultation with the source material—the notes from the workshops. Differences in coding were clarified through discussion between the two researchers. While no intercoder reliability was calculated to document the process, all sources were coded in duplicate, and all coding differences were resolved, resulting in complete intercoder agreement. MAXQDA and Nvivo were used to simplify the coding process. The result was a list of topics for each domain.

### Online voting

To allow participants from different groups to see and evaluate the input of the other groups, an online voting system was set up using LimeSurvey so that participants could provide feedback and refine the definition of quality. In this anonymous survey, the participants were asked to provide their country of residence and the stakeholder group they represented. Given the small sample, there was re-identification risk; however, this was deemed acceptable as the information collected via the survey did not introduce additional risks to participants compared with participation in the workshops. Participant information and consent forms reflected the use of personal data for study purposes. Then, they could vote on the relevance of each topic, which was the result of coding the input from the workshops on a 4-point Likert scale (0 = not relevant, 1 = less relevant, 2 = relevant, 3 = highly relevant). Then, they could vote on the relevance of each presented domain on the same scale. Finally, they were given the option to restructure the domains by voting either “domain” or “sub-item of another domain” for each domain. If they chose “sub-item of another domain”, they were asked to specify which domain this could best be subordinated to. No cut-off points were pre-specified. Pretesting functionality, ease of navigation, and understandability was investigated by the study team and additionally with a colleague from Heidelberg University unfamiliar with the topic but experienced in designing online surveys in February 2023. The survey was completed by the participants in the same month.

### Drafting of the definition and final edits

After receiving quantitative feedback from participants, the study team drafted the definition text and sent it out for participants' approval, comments, or changes.

### Sampling

The participants were selected using purposive, convenience, and snowball sampling, aiming to include a broad range of stakeholders with diverse backgrounds residing in European countries. The participants were approached via personal contact, phone, or email from the wider DELIVER consortium and then via email from the researcher team to organize workshop appointments and transfer consent forms and study information leaflets. More information on sampling is provided in the supplementary materials within Annex 1, points 10–13.

### Ethics

The study received ethics approval from Heidelberg University Medical Faculty (study no. S-598/2022).

## Results

The literature search yielded 1 278 papers, of which 1 084 were abstract- and 73 were full-text-screened; in total, 801 papers were included. Further details regarding the search are given in Annex 2. Out of these findings, the initial topic list was constructed, and for each domain, new topics (n) were added to the findings of Righolt et al. [25]: Patient safety (*n* = 16), Effectiveness (*n* = 14), Patient-centredness (*n* = 35), Timeliness (*n* = 21), Efficiency (*n* = 15), Equitability (*n* = 36), and Accessibility (*n* = 35). The initial topic list is given in Annex 5.

A total of 44 participants took part in the World Café workshops: Policy-makers (*n* = 5), Patients (*n* = 6), Providers (*n* = 10), Representatives of vulnerable populations (*n* = 3), and researchers (*n* = 20). While no detailed sociodemographic data were collected from participants, they shared some information voluntarily during the workshops. Within all groups, both men and women were represented, as well as people of different ages. Providers were dentists with varying levels experience, including specialists and generalists; both public and private care providers were represented. Within the representative of vulnerable populations group were professionals working with vulnerable groups within municipal or charity contexts. The group of patients included people of various educational backgrounds with lived experience of both good and bad self-perceived oral health. Represented in this sample are stakeholders from the following European countries: Germany (*n* = 8), Denmark (*n* = 8), the Netherlands (*n* = 8), Portugal (*n* = 8), Sweden (*n* = 5), United Kingdom (*n* = 4), and Malta (*n* = 3). Only for Germany and Denmark are all stakeholder groups covered within this sample. A more detailed table is given in Annex 4.

**Fig. 2:**
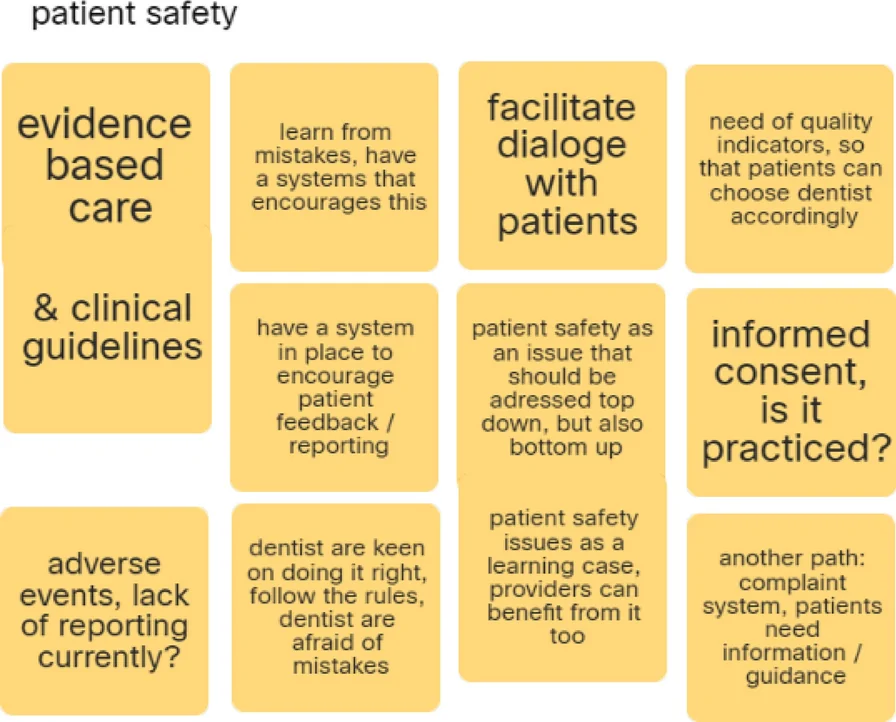
Notes from a policy-maker group about the patient safety domain

Figure 2 presents an example of the whiteboards used to document the group discussions.

All input from the World Café workshops was coded to form general themes: Patient safety (*n* = 10), Effectiveness (*n* = 7), Patient-centredness (*n* = 12), Timeliness (*n* = 8), Efficiency (*n* = 8), Equitability (*n* = 9), and Accessibility (*n* = 8).

Thirty-seven participants took part in the online survey on the relevance of domains and the structure of the definition. The mean across participants was calculated, yielding a mean relevance range of 1.6–2.7 per topic, with a standard deviation of 0.4–0.9. Thus, all topics were seen as relevant (not relevant = 0, less relevant = 1, relevant = 2, highly relevant = 3) enough to include in the final definition. The domains received mean scores of 2.27–2.70, with standard deviations of 0.51–0.72. Therefore, all domains were also considered relevant.

The voting on the structure of the domains resulted in domains receiving a mean score of 0.6–0.9, where 0 = 'not a domain on its own', and 1 = 'a domain on its own', with a standard deviation of 0.3–0.5. As all domains were voted to have at least a mean of 0.6, all domains were kept, with the “new” domain of accessibility being placed third in terms of being a self-sufficient domain. While there were no predefined cut-off points, the study team decided that it would be sensible to categorize the domains and topics by rounding to the next integer, i.e., a majority vote. Undue results could be corrected by the stakeholders in the next step. The voting results were compared with alternative calculations, in which equal weights were applied to the grouped means by stakeholder group and by country, respectively. This revealed no significant difference; the result is shown in Annex 3.

### Final editing

Given that participants voted that all domains and topics were relevant and agreed on the structure, there were no further changes to the World Café workshop outputs. After gathering feedback on the wording from the participants, the final definition of oral healthcare quality was:

“*Quality oral healthcare refers to the contribution of healthcare to optimal oral health. This entails that care alleviates oral health inequity, is comprehensive, empowers patients, mitigates harm and encourages learning systematically, improves the well-being, minimizes waste of resources and is provided at the right time. To maximize quality, care should be equitable, accessible, patient-centered, safe, effective, efficient and timely*.”

**Fig. 3:**
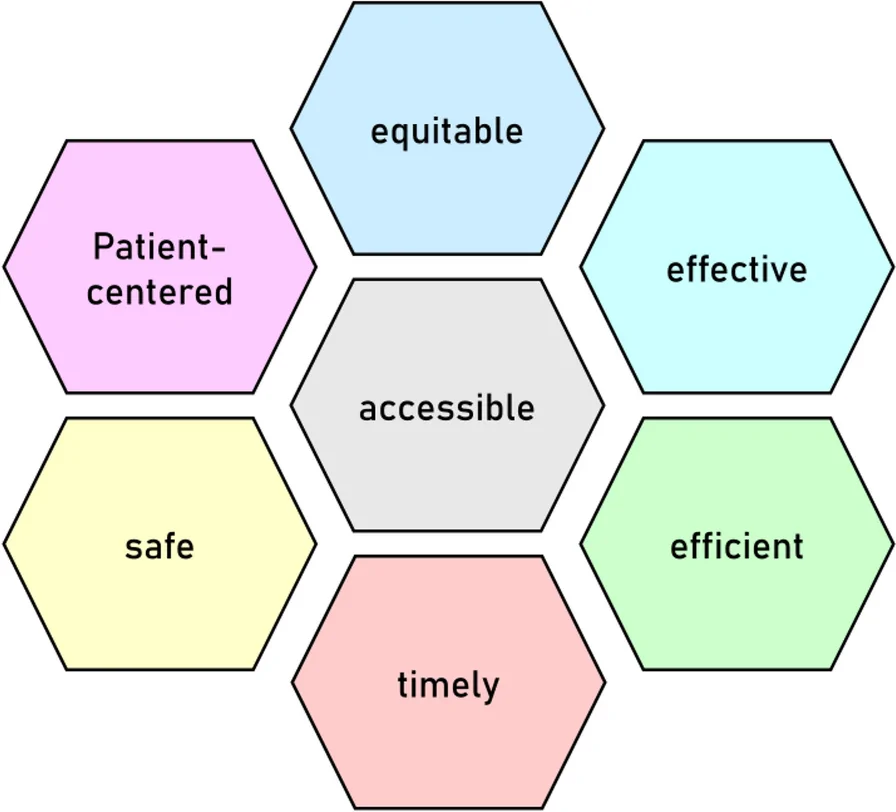
DELIVER domains of quality of oral care

Figure 3 illustrates the seven domains of the DELIVER definition for quality of oral care: equitability, accessibility, timeliness, patient-centredness, efficiency, safety, and effectiveness.

Table 1 shows identified sub-items per domain of the DELIVER definition for quality of oral care.

**Table 1:** Domains and selected sub-items of the DELIVER definition for quality of oral care

Domains of quality	Sub-items of the domain (selection)
Equitability	Care is affordable Care addresses socioeconomic, demographic, gender, geographic, and health-related inequality Efforts are targeted to vulnerable groups
Accessibility	Care is comprehensive and covers preventive, curative and rehabilitative care Care is provided to the whole population
Patient-centredness	Patients are empowered (autonomy, transparency) Patients are involved in treatment planning and decision-making Patients are supported to self-care/self-manage their oral health
Safety	Harmful waiting times, adverse events and over- and undertreatment are minimized or mitigated Medication use is appropriate Continuity of care is assured
Effectiveness	Providers have sufficient skill for their scope of practice Accurate assessment and diagnosis precede care Care is evidence-based and supported by guidelines
Efficiency	The health care system is planned according to needs Care is evaluated (regularly) and efficient care is prioritized Resources are managed well, and waste is minimized
Timeliness	Emergency care is available when needed The health care system addresses disparities in timely access Public health measures for oral health are implemented

## Discussion

This study presents, to our knowledge, the first definition for quality of oral healthcare, which was developed together with all relevant stakeholder groups from various European countries. Drawing on a five-step deliberative approach and engaging diverse stakeholders from various countries, the resulting definition emphasizes that care should be equitable, accessible, patient-centered, safe, effective, efficient, and timely. In addition, the derived list of sub-items provides concrete cues for translating the definition into tangible intervention points.

This study aligns with a previously reported definition [25] corroborating the high relevance of access to care in the oral health context in Europe. While access to care was not a separate domain in the IOM definition [24], the working definition by Righolt et al. [25] suggested that access might warrant particular attention in the context of oral healthcare. This aligns with previous literature that stresses the lack of access to oral healthcare in many countries worldwide [27, 28], including due to high out-of-pocket payments for dental care [5, 7]. Therefore, it can be argued that current oral health systems struggle with access more than other healthcare specialties due to dentistry’s legacy, which has been termed the dental-medical divide [29]. Then again, globally, the high relevance of access to all medical care is also appreciable as it is a target of the sustainable development goals of the United Nations to achieve universal health coverage [6]. Consequently, it can be argued that while any distinct definition of quality healthcare may be desirable for higher specificity and easier operationalization, the fundamental concepts of quality apply to all healthcare. Here, careful consideration must be given to allow for specialty ownership while also avoiding isolation.

Financial protection is both an enabling condition for access and a final outcome of access, and it is access that links quality health services and quality health systems [30, 31]. The implication of this linkage between quality care services and quality health systems underscores the high relevance of health systems interventions for quality improvement. The co-development of a definition of healthcare quality, as presented in this study, can provide the normative direction needed to develop complex health system interventions.

It is important to highlight that this definition both aligns with documents of the World Dental Federation (FDI) as the leading oral health professional organization and supports filling gaps identified previously by FDI. FDI’s policy statement on quality in dentistry underlined the importance of cross-stakeholder collaboration and the need for a shared understanding of quality [32]; both aspects are addressed by the co-developed definition presented. Moreover, the presented definition can have a concrete impact on FDI’s advocacy and policy-making, as it strengthens the evidence base of the definition from Righolt et al. [25] already used within FDI’s guidance documents on quality [33].

The presented definition shows considerable overlap with leading international publications in both content and application. The WHO, the World Bank, and the OECD define quality care in terms of effectiveness, safety, people-centeredness, timeliness, equitability, and efficiency, which are all covered by the presented definition, as well as integration, but accessibility is missing [34]. As highlighted before, accessibility is of particular importance in oral health care, even in high-income countries. On the other hand, it is has become apparent that too much focus has been been placed on accessibility, neglecting the other dimensions of quality, especially in low- and-middle-income-countries [35]. As access to oral healthcare is a global problem, this is a chance to learn from the mistakes made in other parts of the health system that now require a complete overhaul [35]. The presented five-step process can inspire similar activities in other contexts and serve as an operational example of quality planning guidance from WHO. In their planning guide for quality health services, WHO describes eight elements of the national quality policy and strategy approach, two of which can be implemented via the presented process: local definition of quality and stakeholder engagement [36]. The document explicitly highlights the need for “develop[ing] a shared understanding among stakeholders of quality, usually articulated in an agreed […] definition” [36], which is arguably relevant for all levels of health care.

The definition of quality developed in this study is more comprehensive, both in topic breadth and stakeholder engagement, than those in past studies and thus provides deeper insight into what quality of oral healthcare actually means to stakeholders active in or affected by oral healthcare. The use of deliberative and participatory methods for the co-development of a definition of quality of care is seminal, as this endeavour is currently dominated by opinion pieces and institutional contributions [30, 37], and the transformative potential for healthcare research is recognized, as it can greatly improve research relevance [38, 39]. A previous study only included English primary care dentists [40], in which they agreed to include appropriateness, technical quality, continuity of care, and empathy in addition to the dimensions of our definition. The exclusion of other stakeholders leaves room for ambiguity or even unintelligibility, creating a barrier to collaboration and combined efforts with other stakeholders to improve quality.

Comparing the co-development process applied in this study with other deliberative methods of stakeholder engagement in health policy reveals key differences and similarities. Compared with “Dental Policy Labs”, which exclusively invited professional stakeholders for expert input with a predefined goal [41], the presented approach aimed at broader stakeholder inclusion to address a more general question. Policy dialogues [42] are another method of stakeholder engagement, focusing on high-priority issues and allowing for dialogue (not debate) concerning a specific topic such as “technology use in long-term care” [43]; it is compared to the approach applied in this study in Table 2. While both methods seek to provide a forum for open discussion and context-specific input, policy dialogues are targeted toward concrete problems and their potential solution, whereas the presented co-development of a definition is seeking to provide a common language. Hence, it could be argued that defining this common goal could enable and improve follow-up processes, such as a targeted policy dialogue.

**Table 2:** Comparison of policy dialogues and the presented co-development process of the study

Policy dialogues [42]	Five-step process: co-development of oral healthcare quality
Addresses high-priority issue	Addresses foundational gap
Dialogue provides opportunities to discuss the problem, options to address it, and implementation considerations	Allows for discussion connecting key topics to a structured framework, organizing important aspects into a common understanding
Informed by a pre-circulated policy brief and by a discussion about factors influencing the policy-making process	Informed by literature screening, but prioritizing stakeholder discussions for final outcome
Ensures fair representation of those involved in or affected by future decisions	Ensures broad representation, ensuring fairness
Engages a facilitator	Engages a facilitator
Follows a rule about not attributing comments to individuals	Confidentiality is assured, following rules
Does NOT aim for consensus, but allows it	Aims specifically for consensus, creating a common ground
Production of outputs and follow-up activities to support action	Production of final definition, follow-up activities possible such as situational analysis, indicator selection, monitoring, and improvement efforts

The definition developed in this study provides the directionality and common understanding needed for quality improvement and quality measurement, which were hitherto lacking. This applies, for example, to quality indicator development [44], where more consensus and concentrated efforts are needed [45]. Within the DELIVER project, the definition of quality oral healthcare is used to support quality indicator consensus, the gathering of data relevant to quality improvement, and the development of surveys, interview guides, training tools, and quality improvement interventions. The definition provides a comprehensive framework for further research and discussions about quality and can be applied at all levels of healthcare and health systems. Future research could include testing and evaluating the usefulness and adaptability in various contexts, especially in low-and middle-income countries, and for various purposes. The definition of quality oral healthcare provided here is a starting point and a tool. Its value can only be realized if it leads to action for which it created a common groundwork. Defining clearly what we expect from healthcare and health systems can lead to better accountability and performance. Implementing such a definition in policy-making practice could include evaluating current or future policies regarding their impact across the quality domains. For example, how will a policy that implements oral health screening in preschools affect accessibility? Will it improve or decrease the efficiency of the health system? Does it have the potential to improve or worsen equitability? The definition, therefore, provides a structured way to evaluate suggested policies comprehensively with a focus on stakeholder needs. Context-specific sub-items, as presented in Table 1, can further facilitate this process.

The present study is not without limitations. It must be stressed that the normative definition developed in this study reflects current societal values and beliefs and thus must be updated regularly to track societal developments. This study does not provide evidence on how best to update a once-established definition of quality of care and when to undertake such efforts. Future studies could evaluate differences between completely or partially repeated applications of the presented co-development process.

Additionally, the relatively small sample of Northern/Western European participants can limit its applicability in other contexts; however, this study can provide a basis for other contexts and proved the feasibility of such a deliberative exercise to tackle foundational abstract terms with stakeholders of various backgrounds, including non-academics. While the different stakeholder groups and countries had different numbers of participants, the researchers were aware of potential imbalances from both the numerical and power perspectives. As such, the workshops were stakeholder-specific. Each stakeholder group input was equally weighted in the qualitative analysis, and the voting was checked for undue numerical influence of groups. Of course, there might be remaining causes for asymmetries, such as between participants and the facilitating researchers. By clearly defining roles and rules, and by emphasizing the value of stakeholder input for the study through stakeholder engagements and self-reflection within the researcher team, we sought to limit this. While potentially interesting, the study did not focus on analyzing differences in prioritization of specific topics or domains by certain stakeholder groups. However, the relatively high agreement seen in the voting results can be interpreted as a high level of consensus; this may be due to high pre-existing consensus or due to the process, which amalgamated the inputs from each stakeholder into common themes that were then perceived as highly agreeable. It may also be due to the study's context being at a rather high international level; more contested topics may appear if the process is repeated in a more local and specific context, where stakeholders may perceive higher stakes at a personal level.

Consensus on the final definition was determined via the final feedback round. While this was an opportunity for all participants to voice their agreement or disagreement, this mechanism is also limited in quantifying the achieved consensus, but the voting results can serve as a proxy showing high agreement. The presented five-step process of co-development does not include measurements of disagreement for each of the steps; again, the voting results are the only concrete measurement of this kind. Hence, if the goal of a future project were to document disagreement in addition to arriving at a consensus result, further steps would need to be taken. This could include targeted qualitative note-taking during the workshops or a process evaluation, possibly including a survey or interviews.

The literature screening was intentionally pragmatic. While the search strategy was replicating a systematic review, further steps were aligned with the goals of (1) familiarization of the researchers with the topic and (2) creation of an initial topic list that could be used to support the workshops. While this risks missing more topics than a true systematic review, it was deemed appropriate, as the initial topic list was not the main research output. The main research output relies on the involved stakeholders as they were in charge of naming the important topics for each of the definition’s domains. Therefore, the impact of including additional topics in the initial topics list is expected to be minimal. The same applies to “short-cuts” taken in screening: because some topics were included using only information available from abstracts, there might be some topics that were missed and some nuance that was not recorded. The minor impact this could have on the co-developed definition was deemed acceptable considering resource constraints.

The co-development presented within this study was limited to participation in the study and parts of the analysis and interpretation (approving themes, final editing of the definition). Future studies researching normative definitions could explore additional steps of co-development in the study design and the interpretation of results, which could further increase the relevance of research outputs for the involved stakeholders.

The selection of the definition by Righolt [25] as a starting point might have had a substantial impact on the outcome of this study, and many competing frameworks could have been used [46]. Nevertheless, this starting point was deemed appropriate as there was already evidence of its usefulness within the field of oral health [25, 33]. The definition was further validated through the presented five-step co-development process.

The strengths of this study include the application of a deliberative method to tap into the knowledge and experience of relevant stakeholder groups of diverse backgrounds. Also, the thorough preparation of the workshops and the continued stakeholder feedback possibilities throughout the project can be highlighted.

## Conclusion

To our knowledge, the present paper presents the most comprehensive definition of the quality of oral care so far. The application of deliberative methods in co-creating the definition highlights the feasibility and success of such methods, even when tackling more abstract healthcare concepts with stakeholders from diverse backgrounds, including citizens. The definition is crucial for driving change in oral health systems as it stimulates normative directionality and continuous reflective learning. The need for periodic reconsideration and adaptation of the definition over time and across other contexts is emphasized, as the study presents a stakeholder-informed Northern-Western European perspective.

## Supplementary Information

Supplementary material 1. Annex 1: COREQ-32 checklist. Annex 2: Overview literature review, search strategy and search results. Annex 3: Voting results sensitivity checks. Annex 4: Detailed list of non-researcher participants by country and stakeholder group. Annex 5: Initial topic list derived from the literature screening and used as supporting material in workshops.

## Data Availability

Additional to the data presented in the supplemental materials, other study data are available from the authors upon reasonable request.
