# Supplementary material for: Reconsidering how we define quality: insights from the co-development of an actionable definition of oral healthcare quality
Source: Health Res Policy Syst. 2026 Sep 11;24:77. doi: 10.1186/s12961-026-01536-8 (PMC13573396; doi:10.1186/s12961-026-01536-8)
Supplement: Supplementary file 1 [file 12961_2026_1536_MOESM1_ESM.docx]

**Supplemental Materials**

- **Annex 1:** COREQ-32 Checklist
- **Annex 2:** Overview Literature Review, search strategy and search results
- **Annex 3:** Voting results sensitivity checks
- **Annex 4:** Detailed list of non-researcher participants by country and stakeholder group
- **Annex 5:** initial topic list derived from the literature screening and used as supporting material in workshops

**Annex 1: COREQ-32 Checklist**

**Consolidated criteria for reporting qualitative studies (COREQ): 32-item checklist**

Personal Characteristics

1) *Interviewer/facilitator*

*Which author/s conducted the interview or focus group?*

a. ML and ZS conducted the workshops, aided by: Valeska Fehrer, Kasper Rosing, Paulo Melo, Maria Pereira, Matthew Byrne, Shaila Akter

2) *Credentials*

*What were the researcher's credentials? E.g. PhD, MD*

a. ML: dentist+doctoral student, ZS: Msc+PhD student, Valeska Fehrer: Msc+PhD student, Kasper Rosing PhD, Paulo Melo: PhD, Maria Pereira: DMD, Matthew Byrne: PhD, Shaila Akter: PhD student, Stefan Listl: PhD

3) *Occupation*

*What was their occupation at the time of the study?*

a. All where academics in the oral health field

4) *Gender*

*Was the researcher male or female?*

a. All co-authors are male

5) *Experience and training*

*What experience or training did the researcher have?*

a. The supervising researcher and the aiding researchers have extensive experience in qualitative research, for the first two authors this was the first original qualitative research, but they received extensive training and supervision

*Relationship with participants*

6) *Relationship established*

*Was a relationship established prior to study commencement?*

a. All participants had a connection to at least one of the researchers of the DELIVER consortium, or were recruited via such direct contacts

7) *Participant knowledge of the interviewer*

*What did the participants know about the researcher? e.g. personal goals, reasons for doing the research*

a. Participants received a short introduction about the DELIVER project as a whole, which has the goal to improve quality of oral healthcare in Europe, the participants knew the academic background of the researchers

8) Interviewer characteristics

*What characteristics were reported about the interviewer/facilitator? e.g. Bias, assumptions, reasons and interests in the research topic?*

*The moderators all had a background in oral public health research coming from diverse backgrounds both culturally (various European and non-European countries) and scientifically (dentistry, medicine, economics); no self-reflection of potential biases, assumptions or interests was carried out.*

Domain 2: study design

Theoretical framework

9) Methodological orientation and Theory

What methodological orientation was stated to underpin the study? e.g. grounded theory, discourse analysis, ethnography, phenomenology, content analysis

a. The methodologic underpinning of this study was a five-step process including 1) literature screening to familiarize the researchers with the potential topics 2) compilation of starting list to summarize previously gathered information 3)World café type workshops to co-develop definition based on key-topics coming from stakeholders 4) Thematic analysis to identify themes across workshop outputs 5) Voting and final editing to approve summarized findings from the stakeholders’ perspective Participant selection

10) Sampling

How were participants selected? e.g. purposive, convenience, consecutive, snowball

a. Purposive, convenience and snowball sampling

11) Method of approach

How were participants approached? e.g. face-to-face, telephone, mail, email

a. Approached via email from the researcher team, from other Consortium contacts also via other methods such as phone call/ text, personal contact or email.

12) Sample size

How many participants were in the study?

a. 44

13) Non-participation

How many people refused to participate or dropped out? Reasons?

a. See table below. No reasons collected.

| Group | No-response | Declined | Accepted | Drop-out before workshop | Drop-out voting or after | Completed full study |
| --- | --- | --- | --- | --- | --- | --- |
| Policymakers | 18 | 8 | 5 | 0 | 0-2 | 3-7/5 |
| Patients | 11 | 4 | 7 | 1 | 2 | 4 |
| Providers | 12 | 7 | 10 | 0 | 1 | 9 |
| Vulnerable Populations Representatives | 21 | 6 | 3 | 0 | 2 | 1 |
| Researcher | 0 | 0 | 20 | 0 | 0-4 | 16-20 |
| Total | 62 | 25 | 45 | 1 | 7 | 37 |

Setting

14) Setting of data collection

Where was the data collected? e.g. home, clinic, workplace

a. In person meeting of researchers: professional setting during Consortium meeting
b. Other participants: online meetings: they were at a place of their choice, mostly home, some at work

15) Presence of non-participants

Was anyone else present besides the participants and researchers?

a. no

16) Description of sample

What are the important characteristics of the sample? e.g. demographic data, date

a. No demographic data was collected, roughly speaking all ages between mid-twenties to late sixties were represented

Data collection

17) Interview guide

Were questions, prompts, guides provided by the authors? Was it pilot tested?

a. The discussion prompts were only used to get the discussion started, they were based on the literature, the prompts were only piloted during the meetings of the three co-authors

18) Repeat interviews

Were repeat interviews carried out? If yes, how many?

a. No, everyone took only part once in a workshop, but they had the chance to change the outcome of the research by voting and email discussion of the pre-final output

19) Audio/visual recording

Did the research use audio or visual recording to collect the data?

a. No, only field notes were taken + a flipchart/ a digital whiteboard where the output of the group discussion was documented for all to see

20) Field notes

Were field notes made during and/or after the interview or focus group?

a. yes

21) Duration

What was the duration of the interviews or focus group?

a. Approx. 90min, 10min per each domain + introduction

22) Data saturation

Was data saturation discussed?

a. Yes, we did two rounds of recruitment + workshops for each stakeholder group to be sure the most important topics are identified

23) Transcripts returned

Were transcripts returned to participants for comment and/or correction?

a. No, as there were no transcripts. But participants had access to the whiteboard the whole time during the meeting and were send the pre-final output for comments/ changes and confirmation

Domain 3: analysis and findings

Data analysis

24) Number of data coders

How many data coders coded the data?

a. Two

25) Description of the coding tree

Did authors provide a description of the coding tree?

a. We used the quality domains as codes and sorted the fitting topics together with participants into these domains

26) Derivation of themes

Were themes identified in advance or derived from the data?

a. Themes were identified before the participant data was collected, but there was the possibility for the participants to express other theme needs

27) Software

What software, if applicable, was used to manage the data?

a. MS word, MS excel, MAXQDA, Nvivo

28) Participant checking

Did participants provide feedback on the findings?

a. Yes, quantitative through voting and qualitative through email comments/ changes to the pre-final draft of definition

Reporting

29) Quotations presented

Were participant quotations presented to illustrate the themes / findings? Was each quotation identified? e.g. participant number

a. We can present an example of the whiteboards of the online meetings

30) Data and findings consistent

Was there consistency between the data presented and the findings?

a. yes

31) Clarity of major themes

Were major themes clearly presented in the findings?

a. Yes

32) Clarity of minor themes

Is there a description of diverse cases or discussion of minor themes?

a. Already during the workshops the goal was to achieve a list of only major themes, thus leading to consolidation of minor themes from the very beginning.

**Annex 2: Overview Literature Review, search strategy and search results**

| Domain | Patient safety | Effectiveness | Patient-centeredness | Timeliness | Efficiency | Equitability | Accessibility |
| --- | --- | --- | --- | --- | --- | --- | --- |
| Titles screened | 195 | 191 | 103 | 75 | 117 | 348 | 249 |
| Abstract screened | 122 | 178 | 82 | 62 | 60 | 337 | 243 |
| Full text screened | 17 | 2 | 11 | 4 | 4 | 8 | 27 |

| **Domain** | **Search strategy Pubmed** | **Search results** |
| --- | --- | --- |
| **Patient safety** | ((((((((("Oral Health"[Mesh]) OR oral health*[tiab]) OR oral care[tiab]) OR Dental health*[tiab]) OR dental care[tiab])) AND (("Quality of Health Care"[Mesh]) OR quality[tw])))  AND  ((((("Safety"[Mesh:NoExp]) OR "Safety Management"[Mesh:NoExp]) OR "Patient Safety"[Mesh]) OR "Patient Harm"[Mesh]) OR safety[tiab]))  Sort by: Author Filters: published since 2018 | 195 |
| **Effectiveness** | ((("Comparative Effectiveness Research"[Mesh] OR Effectiv*[ti])) AND ((((((("Oral Health"[Mesh]) OR oral health*[tiab]) OR oral care[tiab]) OR Dental health*[tiab]) OR dental care[tiab]))  AND  (("Quality of Health Care"[Mesh]) OR quality[tw])))  Sort by: Author Filters: published since 2018 | 191 |
| **Patient-centeredness** | ((((((((("Oral Health"[Mesh]) OR oral health*[tiab]) OR oral care[tiab]) OR Dental health*[tiab]) OR dental care[tiab])) AND (("Quality of Health Care"[Mesh]) OR quality[tw])))  AND  (((Patient-Focus*[tiab]) OR patient oriented[tiab]) OR ((("Patient-Centered Care"[Mesh:NoExp]) OR "Patient Navigation"[Mesh]) OR patient centered*[tiab])))  Sort by: Author Filters:  since 2018 | 103 |
| **Timeliness** | ((((((((("Oral Health"[Mesh]) OR oral health*[tiab]) OR oral care[tiab]) OR Dental health*[tiab]) OR dental care[tiab])) AND (("Quality of Health Care"[Mesh]) OR quality[tw])))  AND  (((("Time-to-Treatment"[Mesh]) OR (timeliness[tiab] OR timely[tiab])) OR Time-to-Treatment*[tiab]))  Sort by: Author Filters: published since 2018 | 75 |
| **Efficiency** | (((("Efficiency"[Mesh]) OR efficienc*[tiab])) AND ((((((("Oral Health"[Mesh]) OR oral health*[tiab]) OR oral care[tiab]) OR Dental health*[tiab]) OR dental care[tiab]))  AND  (("Quality of Health Care"[Mesh]) OR quality[tw])))  Sort by: Author Filters: published since 2018 | 117 |
| **Equitability** | ((((("Health Status Disparities"[Mesh] OR disparit*[tiab] OR equitab*[tiab])) OR inequit* [tiab])) AND  ((((((("Oral Health"[Mesh]) OR oral health*[tiab]) OR oral care[tiab]) OR Dental health*[tiab]) OR dental care[tiab])) AND (("Quality of Health Care"[Mesh]) OR quality[tw])))  Sort by: Author Filters: published since 2018 | 348 |
| **Accessibility** | ((((((((("Oral Health"[Mesh]) OR oral health*[tiab]) OR oral care[tiab]) OR Dental health*[tiab]) OR dental care[tiab])) AND (("Quality of Health Care"[Mesh]) OR quality[tw])))  AND  ("Health Services Accessibility"[Mesh]))  Sort by: Author Filters: published since 2018 | 249 |

Searches were done using Pubmed, text / abstracts were only screened if available freely or through University of Heidelberg subscription and if the language of the abstract and/or text was english, german or french

Searches were done between 19.09.2022 and 30.09.2022

Literature screening was done in September and October 2022

# Patient safety

According to “An International Working Definition for Quality of Oral Healthcare” (Righolt et. al, 2019) aims are:

In order to be considered safe, oral healthcare should

• Avoid, mitigate, or minimize adverse events.

• Avoid or minimize medical errors and treatment errors (this includes over- and undertreatment).

• Advocate a blame-free culture to facilitate quality improvement.

• Learn from safety incidents to improve the quality of care.

• Enact minimum safety standards.

Number of titles screened: 195

Number of abstracts screened: 122

Number of full texts screened: 17

| **Possible topics deduced from literature** | **Reference** |
| --- | --- |
| Oral health literacy | Previously covered (old literature screening see Righolt et. al, 2019 |
| Data privacy | Previously covered (old literature screening see Righolt et. al, 2019 |
| Data security | Previously covered (old literature screening see Righolt et. al, 2019 |
| Risk assessment to measure disease | Previously covered (old literature screening see Righolt et. al, 2019 |
| Clinical audit | Previously covered (old literature screening see Righolt et. al, 2019 |
| Adverse events | Previously covered (old literature screening see Righolt et. al, 2019 |
| Trigger tools | Previously covered (old literature screening see Righolt et. al, 2019 |
| Safety checklist | Previously covered (old literature screening see Righolt et. al, 2019 |
| Reporting systems | Previously covered (old literature screening see Righolt et. al, 2019 |
| Medical error/patient safety incidents | Previously covered (old literature screening see Righolt et. al, 2019 |
| hazardous healthcare  conditions | Previously covered (old literature screening see Righolt et. al, 2019 |
| Unintended harm | Previously covered (old literature screening see Righolt et. al, 2019 |
| Trusting dentist-patient relationship | Previously covered (old literature screening see Righolt et. al, 2019 |
| Prescribing medicine | Previously covered (old literature screening see Righolt et. al, 2019 |
| Medical interaction/medically complex patients | Previously covered (old literature screening see Righolt et. al, 2019 |
| Near misses | Previously covered (old literature screening see Righolt et. al, 2019 |
| Standardized guidelines for treatment | Previously covered (old literature screening see Righolt et. al, 2019 |
| Standardized diagnostic codes | Previously covered (old literature screening see Righolt et. al, 2019 |

| Continued education | 1. Yurt A, Ayrancıoğlu C, Kılınç G, Ergönül E. Knowledge, attitude, and behavior of Turkish dentists about radiation protection and radiation safety. Dentomaxillofac Radiol. 2022 Jan 1;51(1):20210120. doi: 10.1259/dmfr.20210120. Epub 2021 Jul 14. PMID: 34260311; PMCID: PMC8693318. |
| --- | --- |
| Radiation control | 1. Yurt A, Ayrancıoğlu C, Kılınç G, Ergönül E. Knowledge, attitude, and behavior of Turkish dentists about radiation protection and radiation safety. Dentomaxillofac Radiol. 2022 Jan 1;51(1):20210120. doi: 10.1259/dmfr.20210120. Epub 2021 Jul 14. PMID: 34260311; PMCID: PMC8693318. 2. Righolt AJ, Duijster D, van der Wees PJ, Listl S, Smits KPJ. Dutch oral health care quality measures: a modified Delphi study. Int Dent J. 2020 Aug;70(4):277-286. doi: 10.1111/idj.12566. Epub 2020 Apr 14. PMID: 32285939; PMCID: PMC7497121. |
| Effective communication / language barrier | 1. Woll A, Quick KK, Mazzei C, Selameab T, Miller JL. Working With Interpreters as a Team in Health Care (WITH Care) Curriculum Tool Kit for Oral Health Professions. MedEdPORTAL. 2020 Apr 10;16:10894. doi: 10.15766/mep_2374-8265.10894. PMID: 32352031; PMCID: PMC7187915. |
| Inspection (method / framework) | 1. Weenink JW, Wallenburg I, Leistikow I, Bal RA. Publication of inspection frameworks: a qualitative study exploring the impact on quality improvement and regulation in three healthcare settings. BMJ Qual Saf. 2021 Oct;30(10):804-811. doi: 10.1136/bmjqs-2020-011337. Epub 2020 Dec 2. PMID: 33268448; PMCID: PMC8461449. |
| Pandemic / Covid-19  (guidlines, PPE, testing, infrastructure adaption, patient care management) | 1. Vieira-Meyer APGF, Coutinho MB, Santos HPG, Saintrain MV, Candeiro GTM. Brazilian Primary and Secondary Public Oral Health Attention: Are Dentists Ready to Face the COVID-19 Pandemic? Disaster Med Public Health Prep. 2022 Feb;16(1):254-261. doi: 10.1017/dmp.2020.342. Epub 2020 Sep 10. PMID: 32907660; PMCID: PMC7684023. 2. Tysiąc-Miśta M, Bulanda S. The utilization of rapid serological tests in COVID-19 diagnostics - a high risk of false-negative results in outpatient care, with particular emphasis on dental treatment. Med Pr. 2021 Apr 9;72(2):155-162. doi: 10.13075/mp.5893.01034. Epub 2020 Oct 22. PMID: 33095209. 3. Expósito-Delgado AJ, Ausina-Márquez V, Mateos-Moreno MV, Martínez-Sanz E, Del Carmen Trullols-Casas M, Llamas-Ortuño ME, Blanco-González JM, Almerich-Torres T, Bravo M, Martínez-Beneyto Y. Delivery of Health Care by Spanish Dental Hygienists in Private and Public Dental Services during the COVID-19 De-Escalation Phase (June 2020): A Cross-Sectional Study. Int J Environ Res Public Health. 2021 Aug 5;18(16):8298. doi: 10.3390/ijerph18168298. PMID: 34444048; PMCID: PMC8394270. 4. Bizzoca ME, Campisi G, Muzio LL. Covid-19 Pandemic: What Changes for Dentists and Oral Medicine Experts? A Narrative Review and Novel Approaches to Infection Containment. Int J Environ Res Public Health. 2020 May 27;17(11):3793. doi: 10.3390/ijerph17113793. PMID: 32471083; PMCID: PMC7312076. |
| Interprofessional working / communication | 1. Sturrock A, Preshaw PM, Hayes C, Wilkes S. General dental practitioners' perceptions of, and attitudes towards, improving patient safety through a multidisciplinary approach to the prevention of medication-related osteonecrosis of the jaw (MRONJ): a qualitative study in the North East of England. BMJ Open. 2019 Jun 17;9(6):e029951. doi: 10.1136/bmjopen-2019-029951. PMID: 31213454; PMCID: PMC6597093. 2. Dragan IF, Veglia V, Geisinger ML, Alexander DC. Dental Care as a Safe and Essential Part of a Healthy Pregnancy. Compend Contin Educ Dent. 2018 Feb;39(2):86-91; quiz 92. PMID: 29388782. |
| Cultural safety | 1. Shrivastava R, Campeau R, Couturier Y, Torrie J, Girard F, Marie-Pierre B, Emami E. Anthropological perspectives on Miyupimaatisiiun and the integration of oral health in primary care in the Cree communities of Northern Quebec. PLoS One. 2020 Apr 9;15(4):e0231406. doi: 10.1371/journal.pone.0231406. PMID: 32271846; PMCID: PMC7144986. 2. Linton JM, Green A; COUNCIL ON COMMUNITY PEDIATRICS. Providing Care for Children in Immigrant Families. Pediatrics. 2019 Sep;144(3):e20192077. doi: 10.1542/peds.2019-2077. Epub 2019 Aug 19. PMID: 31427460. |
| Additionaly to patient safety, ocupational and instrumental safety was considered | 1. Salkinoja P, Tuononen T, Suominen AL, Lammintakanen J, Raittio E. Oral health care quality as viewed by leading dentists and their superiors - a qualitative study. Acta Odontol Scand. 2022 Jan;80(1):38-43. doi: 10.1080/00016357.2021.1936161. Epub 2021 Jun 21. PMID: 34153210. |
| Medical history | 1. Righolt AJ, Duijster D, van der Wees PJ, Listl S, Smits KPJ. Dutch oral health care quality measures: a modified Delphi study. Int Dent J. 2020 Aug;70(4):277-286. doi: 10.1111/idj.12566. Epub 2020 Apr 14. PMID: 32285939; PMCID: PMC7497121. |
| Communication / follow-up | 1. Raimondi DC, Bernal SCZ, Matsuda LM. Patient safety culture from the perspective of workers and primary health care teams. Rev Saude Publica. 2019;53:42. doi: 10.11606/S1518-8787.2019053000788. Epub 2019 May 6. PMID: 31066820; PMCID: PMC6536107. |
| Patient engagement to improve safety | 1. Obadan-Udoh E, Panwar S, Yansane AI, Nayudu A, Pang J, White J, Kalenderian E. Are Dental Patients Concerned About Safety? An Exploratory Study. J Evid Based Dent Pract. 2020 Sep;20(3):101424. doi: 10.1016/j.jebdp.2020.101424. Epub 2020 Mar 3. PMID: 32921378. 2. Obadan-Udoh E, Van der Berg-Cloete S, Ramoni R, Kalenderian E, White JG. Patient-Reported Dental Safety Events: A South African Perspective. J Patient Saf. 2021 Dec 1;17(8):e866-e873. doi: 10.1097/PTS.0000000000000464. PMID: 29369072. 3. Obadan-Udoh EM, Gharpure A, Lee JH, Pang J, Nayudu A. Perspectives of Dental Patients About Safety Incident Reporting: A Qualitative Pilot Study. J Patient Saf. 2021 Dec 1;17(8):e874-e882. doi: 10.1097/PTS.0000000000000863. PMID: 34009866. |
| Strategies for overcoming failure | 1. Lee JR, Gil YM. Exploring dentists' strategies for overcoming failure in daily dental practice. J Dent. 2022 Jun;121:104079. doi: 10.1016/j.jdent.2022.104079. Epub 2022 Feb 27. PMID: 35235815. |
| Evidence-based dentistry | 1. Krut AG. USE OF EVIDENCE-BASED MEDICINE BY DENTISTS. Wiad Lek. 2022;75(5 pt 1):1100-1104. doi: 10.36740/WLek202205109. PMID: 35758485. |
| Patient mangement / Urgent appointments | 1. Kirton JA, Thompson W, Pearce M, Brown JM. Ability of the wider dental team to triage patients with acute conditions: a qualitative study. Br Dent J. 2020 Jan;228(2):103-107. doi: 10.1038/s41415-020-1199-1. PMID: 31980786. |
| Health literacy, espacially through community engagement / partnerships with community support agencies | 1. Kempster C, Gray J, Burman K. Student-driven partnerships enhancing oral health literacy. Health Promot J Austr. 2021 Feb;32 Suppl 1:98-103. doi: 10.1002/hpja.376. Epub 2020 Nov 24. PMID: 32589306. |
| Terminology of adverse events or similar | 1. Jokstad A. Oral health professionals must use the correct terminology when explaining risks for complications and undesirable health outcomes as a basis for informed consent for clinical treatment. Clin Exp Dent Res. 2019 Aug 21;5(4):313-315. doi: 10.1002/cre2.237. PMID: 31452941; PMCID: PMC6704026. |
| Work environment, especially time-off to go to dentist | 1. Harolds JA. Quality and Safety in Healthcare, Part LXII: Efforts by National Organizations to Promote Resident Wellness. Clin Nucl Med. 2020 Oct;45(10):778-780. doi: 10.1097/RLU.0000000000002885. PMID: 31876823. |
| Medical emergencies | 1. de Bedout T, Kramer K, Blanchard S, Hamada Y, Eckert GJ, Maupome G, John V. Assessing the Medical Emergency Preparedness of Dental Faculty, Residents, and Practicing Periodontists: An Exploratory Study. J Dent Educ. 2018 May;82(5):492-500. doi: 10.21815/JDE.018.058. PMID: 29717073. |
| Integrated electronic health record | 1. Claman DB, Molina JL, Peng J, Fischbach H, Casamassimo PS. Accuracy of Parental Self-Report of Medical History in a Dental Setting: Integrated Electronic Health Record and Nonintegrated Dental Record. Pediatr Dent. 2021 May 15;43(3):230-236. PMID: 34172118. |
| Safety of workforce, especially psychologically | 1. Bsoul EA, Challa SN, Loomer PM. Multifaceted impact of COVID-19 on dental practice: American dental care professionals prepared and ready during unprecedented challenges. J Am Dent Assoc. 2022 Feb;153(2):132-143. doi: 10.1016/j.adaj.2021.07.023. Epub 2021 Aug 7. PMID: 34763816; PMCID: PMC8520822. |

# Effectiveness

According to “An International Working Definition for Quality of Oral Healthcare” (Righolt et. al, 2019) aims are:

In order to be considered effective, oral healthcare should

• Be informed by the most recent available scientific evidence and guidelines.

• Translate scientific evidence into clinical practice.

• Maximize the intended health outcome of care.

• Be provided to the patients who benefit from the care, and aspire to minimize harm.

• Provide the best available care for the needs and preferences of each patient.

Number of titles screened: 191

Number of abstracts screened: 178

Number of full texts screened: 2

| **Possible topics deduced from literature** | **Reference** |
| --- | --- |
| Scientific knowledge/Evidence based care | Previously covered (old literature screening see Righolt et. al, 2019 |
| Care tailored for the target group | Previously covered (old literature screening see Righolt et. al, 2019 |
| Managing available resources | Previously covered (old literature screening see Righolt et. al, 2019 |
| Managing available time | Previously covered (old literature screening see Righolt et. al, 2019 |
| Cost effectiveness | Previously covered (old literature screening see Righolt et. al, 2019 |

| **Possible topics deduced from literature** | **Reference** |
| --- | --- |
| kinesiophobia | 1. Zhang Q, Zhang J, Ran W, Yu S, Jin Y. Effectiveness of cognitive behavioral therapy on kinesiophobia and oral health-related quality of life in patients with temporomandibular disorders, study protocol for a randomized controlled trial. Medicine (Baltimore). 2020 Nov 20;99(47):e23295. doi: 10.1097/MD.0000000000023295. PMID: 33217860; PMCID: PMC7676583. |
| (O)HRQOL, OHIP-14, PROMS, General Oral Health Assessment Index (GOHAI), etc., quality of life (in general), quality-adjusted-life-year | 1. Zhang Q, Zhang J, Ran W, Yu S, Jin Y. Effectiveness of cognitive behavioral therapy on kinesiophobia and oral health-related quality of life in patients with temporomandibular disorders, study protocol for a randomized controlled trial. Medicine (Baltimore). 2020 Nov 20;99(47):e23295. doi: 10.1097/MD.0000000000023295. PMID: 33217860; PMCID: PMC7676583. 2. Widbiller M, Knüttel H, Meschi N, Durán-Sindreu Terol F. Effectiveness of endodontic tissue engineering in treatment of apical periodontitis: A systematic review. Int Endod J. 2022 Jun 14. doi: 10.1111/iej.13784. Epub ahead of print. PMID: 35699668. 3. Ting CC, Ko EC, Chen CC, Chang WY, Tu HP, Chang CS. Effectiveness of an oral function intervention for older Taiwanese people. Gerodontology. 2019 Dec;36(4):374-381. doi: 10.1111/ger.12412. Epub 2019 Jul 5. PMID: 31274226. 4. Tickle M, Ricketts DJN, Duncan A, O'Malley L, Donaldson PM, Clarkson JE, Black M, Boyers D, Donaldson M, Floate R, Forrest MM, Fraser A, Glenny AM, Goulao B, McDonald A, Ramsay CR, Ross C, Walsh T, Worthington HV, Young L, Bonetti DL, Gouick J, Mitchell FE, Macpherson LE, Lin YL, Pretty IA, Birch S. Protocol for a Randomised controlled trial to Evaluate the effectiveness and cost benefit of prescribing high dose FLuoride toothpaste in preventing and treating dEntal Caries in high-risk older adulTs (reflect trial). BMC Oral Health. 2019 May 24;19(1):88. doi: 10.1186/s12903-019-0749-x. PMID: 31126270; PMCID: PMC6534863. 5. Sihra R, Schroth RJ, Bertone M, Martin H, Patterson B, Mittermuller BA, Lee V, Patterson B, Moffatt ME, Klus B, Fontana M, Robertson L. The Effectiveness of Silver Diamine Fluoride and Fluoride Varnish in Arresting Caries in Young Children and Associated Oral Health-Related Quality of Life. J Can Dent Assoc. 2020 Jun;86:k9. PMID: 32543369. 6. Nogueira TE, Aguiar FMO, Esfandiari S, Leles CR. Effectiveness of immediately loaded single-implant mandibular overdentures versus mandibular complete dentures: A 1-year follow-up of a randomized clinical trial. J Dent. 2018 Oct;77:43-50. doi: 10.1016/j.jdent.2018.07.006. Epub 2018 Jul 10. PMID: 30006117. 7. Plessas A, Nasser M. Can we deliver effective alcohol-related brief advice in general dental practice? Evid Based Dent. 2019 Sep;20(3):77-78. doi: 10.1038/s41432-019-0036-3. PMID: 31562406. 8. Mohammadi F, Oshvandi K, Kamallan SR, Khazaei S, Ranjbar H, Ahmadi-Motamayel F, Gillespie M, Jenabi E, Vafaei SY. Effectiveness of sodium bicarbonate and zinc chloride mouthwashes in the treatment of oral mucositis and quality of life in patients with cancer under chemotherapy. Nurs Open. 2022 May;9(3):1602-1611. doi: 10.1002/nop2.1168. Epub 2022 Feb 16. PMID: 35170247; PMCID: PMC8994952. 9. McLister C, Donnelly M, Cardwell C, Moore C, McKenna G. Effectiveness of prosthodontic interventions and survival of remaining teeth in adult patients with shortened dental arches-Protocol for a systematic review and meta-analysis. BDJ Open. 2018 Apr 27;4:17023. doi: 10.1038/bdjopen.2017.23. PMID: 30637120; PMCID: PMC5921535. 10. McLister C, Donnelly M, Cardwell CR, Moore C, O'Neill C, Brocklehurst P, McKenna G. Effectiveness of prosthodontic interventions and survival of remaining teeth in adult patients with shortened dental arches-A systematic review. J Dent. 2018 Nov;78:31-39. doi: 10.1016/j.jdent.2018.02.003. Epub 2018 Feb 21. PMID: 29476794. 11. Lopes CCA, Limirio JPJO, Zanatta LSA, Simamoto VRN, Dechichi P, Limirio APHJO. Effectiveness of Photobiomodulation Therapy on Human Bone Healing in Dentistry: A Systematic Review. Photobiomodul Photomed Laser Surg. 2022 Jul;40(7):440-453. doi: 10.1089/photob.2021.0092. Epub 2022 May 6. PMID: 35527692. 12. Khan S, Khalid T, Bettiol S, Crocombe LA. Non-surgical periodontal therapy effectively improves patient-reported outcomes: A systematic review. Int J Dent Hyg. 2021 Feb;19(1):18-28. doi: 10.1111/idh.12450. Epub 2020 Jul 16. PMID: 32594621. 13. Ho BV, Weijenberg RAF, van der Maarel-Wierink CD, Visscher CM, van der Putten GJ, Scherder EJA, Lobbezoo F. Effectiveness of the implementation project 'Don't forget the mouth!' of community dwelling older people with dementia: a prospective longitudinal single-blind multicentre study protocol (DFTM!). BMC Oral Health. 2019 May 28;19(1):91. doi: 10.1186/s12903-019-0786-5. PMID: 31138273; PMCID: PMC6537198. 14. Gandedkar NH, Darendeliler MA. Combined Orthodontic-Surgical Treatment May Be an Effective Choice to Improve Oral Health-Related Quality of Life for Individuals Affected With Severe Dentofacial Deformities. J Evid Based Dent Pract. 2020 Sep;20(3):101462. doi: 10.1016/j.jebdp.2020.101462. Epub 2020 Jun 16. PMID: 32921382. 15. Fueki K, Inamochi Y, Yoshida-Kohno E, Wakabayashi N. Cost-effectiveness analysis of prosthetic treatment with thermoplastic resin removable partial dentures. J Prosthodont Res. 2021 Feb 24;65(1):52-55. doi: 10.2186/jpr.JPOR_2019_418. Epub 2020 Sep 9. PMID: 32938866. 16. Xia Y, Ma C, Chen J, Witter DJ, Zhang Q, Creugers NHJ. Mandibular residual ridge morphology in relation to complete dentures and implant overdentures-Part II: Predictors for effectiveness of implant overdenture therapy. Clin Implant Dent Relat Res. 2021 Feb;23(1):140-148. doi: 10.1111/cid.12975. Epub 2021 Feb 16. PMID: 33592681. 17. Victory E, Rhiannon ET, Girvan B, Pauline A, Cynthia PM. Cost-effectiveness Analysis of the Dental RECUR Pragmatic Randomized Controlled Trial: Evaluating a Goal-oriented Talking Intervention to Prevent Reoccurrence of Dental Caries in Children. Appl Health Econ Health Policy. 2022 May;20(3):431-445. doi: 10.1007/s40258-022-00720-5. Epub 2022 Mar 17. PMID: 35298778; PMCID: PMC9021113. 18. Martins AFL, Nogueira TE, Morais MO, Oton-Leite AF, Valadares MC, Batista AC, Freitas NMA, Leles CR, Mendonça EF. Effect of photobiomodulation on the severity of oral mucositis and molecular changes in head and neck cancer patients undergoing radiotherapy: a study protocol for a cost-effectiveness randomized clinical trial. Trials. 2019 Feb 1;20(1):97. doi: 10.1186/s13063-019-3196-8. PMID: 30709370; PMCID: PMC6359861. 19. Lopes Martins AF, Nogueira TE, Morais MO, de Sousa-Neto SS, Oton-Leite AF, Valadares MC, Aires Freitas NM, Leles CR, Mendonça EF. Cost-effectiveness randomized clinical trial on the effect of photobiomodulation therapy for prevention of radiotherapy-induced severe oral mucositis in a Brazilian cancer hospital setting. Support Care Cancer. 2021 Mar;29(3):1245-1256. doi: 10.1007/s00520-020-05607-6. Epub 2020 Jul 3. PMID: 32621262. 20. Korenori A, Koji K, Yuki T, Murata T, Sachiko TM, Shunsuke B. Cost-effectiveness of molar single-implant versus fixed dental prosthesis. BMC Oral Health. 2018 Aug 20;18(1):141. doi: 10.1186/s12903-018-0604-5. PMID: 30126400; PMCID: PMC6102921. 21. Ferraz NKL, Nogueira LC, Neiva IM, Ferreira RC, Moreira AN, Magalhães CS. Longevity, effectiveness, safety, and impact on quality of life of low-concentration hydrogen peroxides in-office bleaching: a randomized clinical trial. Clin Oral Investig. 2019 May;23(5):2061-2070. doi: 10.1007/s00784-018-2607-7. Epub 2018 Sep 20. PMID: 30238417. 22. Fattori L, Sendyk M, de Paiva JB, Normando D, Neto JR. Micro-osteoperforation effectiveness on tooth movement rate and impact on oral health related quality of life. Angle Orthod. 2020 Sep 1;90(5):640-647. doi: 10.2319/110819-707.1. PMID: 33378487; PMCID: PMC8032262. 23. Ekici Ö, Dündar Ü, Büyükbosna M. Effectiveness of high-intensity laser therapy in patients with myogenic temporomandibular joint disorder: A double-blind, placebo-controlled study. J Stomatol Oral Maxillofac Surg. 2022 Jun;123(3):e90-e96. doi: 10.1016/j.jormas.2021.06.009. Epub 2021 Jun 24. PMID: 34174507. 24. Duncan HF, Nagendrababu V, El-Karim I, Dummer PMH. Outcome measures to assess the effectiveness of endodontic treatment for pulpitis and apical periodontitis for use in the development of European Society of Endodontology S3-level clinical practice guidelines: A consensus-based development. Int Endod J. 2021 Dec;54(12):2184-2194. doi: 10.1111/iej.13627. Epub 2021 Sep 22. PMID: 34553383. 25. de Resende GP, Nogueira TE, Leles CR. Effectiveness of a simplified method for final impression in complete denture treatment: A pragmatic clinical trial. Gerodontology. 2019 Dec;36(4):365-373. doi: 10.1111/ger.12411. Epub 2019 Jul 5. PMID: 31276220. 26. de Resende CMBM, de Oliveira Medeiros FGL, de Figueiredo Rêgo CR, Bispo ASL, Barbosa GAS, de Almeida EO. Short-term effectiveness of conservative therapies in pain, quality of life, and sleep in patients with temporomandibular disorders: A randomized clinical trial. Cranio. 2021 Jul;39(4):335-343. doi: 10.1080/08869634.2019.1627068. Epub 2019 Jun 15. PMID: 31204605. 27. de Barros Pascoal AL, de Freitas RFCP, da Silva LFG, Oliveira AGRC, Dos Santos Calderon P. Effectiveness of Counseling on Chronic Pain Management in Patients with Temporomandibular Disorders. J Oral Facial Pain Headache. 2020 Winter;34(1):77–82. doi: 10.11607/ofph.2163. Epub 2019 Apr 12. PMID: 30978270. 28. Czwikla J, Herzberg A, Kapp S, Kloep S, Rothgang H, Nitschke I, Haffner C, Hoffmann F. Effectiveness of a Dental Intervention to Improve Oral Health among Home Care Recipients: A Randomized Controlled Trial. Int J Environ Res Public Health. 2021 Sep 3;18(17):9339. doi: 10.3390/ijerph18179339. PMID: 34501925; PMCID: PMC8430536. 29. Clarkson JE, Pitts NB, Fee PA, Goulao B, Boyers D, Ramsay CR, Floate R, Braid HJ, Ord FS, Worthington HV, van der Pol M, Young L, Freeman R, Gouick J, Humphris GM, Mitchell FE, McDonald AM, Norrie JDT, Sim K, Douglas G, Ricketts D. Examining the effectiveness of different dental recall strategies on maintenance of optimum oral health: the INTERVAL dental recalls randomised controlled trial. Br Dent J. 2021 Feb;230(4):236-243. doi: 10.1038/s41415-021-2612-0. Epub 2021 Feb 26. PMID: 33637927; PMCID: PMC7908962. 30. Clarkson JE, Pitts NB, Bonetti D, Boyers D, Braid H, Elford R, Fee PA, Floate R, Goulão B, Humphris G, Needleman I, Norrie JDT, Ord F, van der Pol M, Ramsay CR, Ricketts DNJ, Worthington HV, Young L; INTERVAL Trial Collaboration. INTERVAL (investigation of NICE technologies for enabling risk-variable-adjusted-length) dental recalls trial: a multicentre randomised controlled trial investigating the best dental recall interval for optimum, cost-effective maintenance of oral health in dentate adults attending dental primary care. BMC Oral Health. 2018 Aug 7;18(1):135. doi: 10.1186/s12903-018-0587-2. PMID: 30086747; PMCID: PMC6081817. 31. Bramantoro T, Santoso CMA, Hariyani N, Setyowati D, Zulfiana AA, Nor NAM, Nagy A, Pratamawari DNP, Irmalia WR. Effectiveness of the school-based oral health promotion programmes from preschool to high school: A systematic review. PLoS One. 2021 Aug 11;16(8):e0256007. doi: 10.1371/journal.pone.0256007. PMID: 34379685; PMCID: PMC8357156. 32. Bersezio C, Ledezma P, Mayer C, Rivera O, Junior OBO, Fernández E. Effectiveness and effect of non-vital bleaching on the quality of life of patients up to 6 months post-treatment: a randomized clinical trial. Clin Oral Investig. 2018 Dec;22(9):3013-3019. doi: 10.1007/s00784-018-2389-y. Epub 2018 Feb 17. PMID: 29455372. 33. Baba Y, Sato Y, Owada G, Minakuchi S. Effectiveness of a combination denture-cleaning method versus a mechanical method: comparison of denture cleanliness, patient satisfaction, and oral health-related quality of life. J Prosthodont Res. 2018 Jul;62(3):353-358. doi: 10.1016/j.jpor.2018.01.005. Epub 2018 Feb 7. PMID: 29428169. 34. Anopa Y, Conway DI. Exploring the cost-effectiveness of child dental caries prevention programmes. Are we comparing apples and oranges? Evid Based Dent. 2020 Mar;21(1):5-7. doi: 10.1038/s41432-020-0085-7. PMID: 32221482. |
| pain | 1. Zhang Q, Zhang J, Ran W, Yu S, Jin Y. Effectiveness of cognitive behavioral therapy on kinesiophobia and oral health-related quality of life in patients with temporomandibular disorders, study protocol for a randomized controlled trial. Medicine (Baltimore). 2020 Nov 20;99(47):e23295. doi: 10.1097/MD.0000000000023295. PMID: 33217860; PMCID: PMC7676583. 2. Widbiller M, Knüttel H, Meschi N, Durán-Sindreu Terol F. Effectiveness of endodontic tissue engineering in treatment of apical periodontitis: A systematic review. Int Endod J. 2022 Jun 14. doi: 10.1111/iej.13784. Epub ahead of print. PMID: 35699668. 3. Duncan HF, Nagendrababu V, El-Karim I, Dummer PMH. Outcome measures to assess the effectiveness of endodontic treatment for pulpitis and apical periodontitis for use in the development of European Society of Endodontology S3-level clinical practice guidelines: A consensus-based development. Int Endod J. 2021 Dec;54(12):2184-2194. doi: 10.1111/iej.13627. Epub 2021 Sep 22. PMID: 34553383. 4. de Resende CMBM, de Oliveira Medeiros FGL, de Figueiredo Rêgo CR, Bispo ASL, Barbosa GAS, de Almeida EO. Short-term effectiveness of conservative therapies in pain, quality of life, and sleep in patients with temporomandibular disorders: A randomized clinical trial. Cranio. 2021 Jul;39(4):335-343. doi: 10.1080/08869634.2019.1627068. Epub 2019 Jun 15. PMID: 31204605. 5. de Barros Pascoal AL, de Freitas RFCP, da Silva LFG, Oliveira AGRC, Dos Santos Calderon P. Effectiveness of Counseling on Chronic Pain Management in Patients with Temporomandibular Disorders. J Oral Facial Pain Headache. 2020 Winter;34(1):77–82. doi: 10.11607/ofph.2163. Epub 2019 Apr 12. PMID: 30978270. 6. Alonaizan FA, AlFawaz YF. Is phototherapy effective in the management of post-operative endodontic pain? A systematic review of randomized controlled clinical trials. Photodiagnosis Photodyn Ther. 2019 Jun;26:53-58. doi: 10.1016/j.pdpdt.2019.03.003. Epub 2019 Mar 2. PMID: 30836213. 7. Aggarwal VR, Fu Y, Main CJ, Wu J. The effectiveness of self-management interventions in adults with chronic orofacial pain: A systematic review, meta-analysis and meta-regression. Eur J Pain. 2019 May;23(5):849-865. doi: 10.1002/ejp.1358. Epub 2019 Feb 7. PMID: 30620145. |
| maximum mouth opening | 1. Wu X, Zhu J, Zheng B, Liu J, Wu Z. Effectiveness of low-level gallium aluminium arsenide laser therapy for temporomandibular disorder with myofascial pain: A systemic review and meta-analysis. Medicine (Baltimore). 2021 Dec 30;100(52):e28015. doi: 10.1097/MD.0000000000028015. PMID: 34967349; PMCID: PMC8718212. |
| Anxiety, dental fear | 1. Zhang Q, Zhang J, Ran W, Yu S, Jin Y. Effectiveness of cognitive behavioral therapy on kinesiophobia and oral health-related quality of life in patients with temporomandibular disorders, study protocol for a randomized controlled trial. Medicine (Baltimore). 2020 Nov 20;99(47):e23295. doi: 10.1097/MD.0000000000023295. PMID: 33217860; PMCID: PMC7676583. 2. Gujjar KR, van Wijk A, Kumar R, de Jongh A. Are Technology-Based Interventions Effective in Reducing Dental Anxiety in Children and Adults? A Systematic Review. J Evid Based Dent Pract. 2019 Jun;19(2):140-155. doi: 10.1016/j.jebdp.2019.01.009. Epub 2019 Feb 2. PMID: 31326046. 3. Azher U, Srinath SK, Nayak M. Effectiveness of Bubble Breath Play Therapy in the Dental Management of Anxious Children: A Pilot Study. J Contemp Dent Pract. 2020 Jan 1;21(1):17-21. PMID: 32381795. 4. AlBhaisi IN, Kumar MSTS, Engapuram A, Shafiei Z, Zakaria ASI, Mohd-Said S, McGrath C. Effectiveness of psychological techniques in dental management for children with autism spectrum disorder: a systematic literature review. BMC Oral Health. 2022 May 6;22(1):162. doi: 10.1186/s12903-022-02200-7. PMID: 35524299; PMCID: PMC9074276. |
| depression | 1. Zhang Q, Zhang J, Ran W, Yu S, Jin Y. Effectiveness of cognitive behavioral therapy on kinesiophobia and oral health-related quality of life in patients with temporomandibular disorders, study protocol for a randomized controlled trial. Medicine (Baltimore). 2020 Nov 20;99(47):e23295. doi: 10.1097/MD.0000000000023295. PMID: 33217860; PMCID: PMC7676583. 2. Aggarwal VR, Fu Y, Main CJ, Wu J. The effectiveness of self-management interventions in adults with chronic orofacial pain: A systematic review, meta-analysis and meta-regression. Eur J Pain. 2019 May;23(5):849-865. doi: 10.1002/ejp.1358. Epub 2019 Feb 7. PMID: 30620145. |
| (oral hygiene) self-efficacy | 1. Zhang Q, Zhang J, Ran W, Yu S, Jin Y. Effectiveness of cognitive behavioral therapy on kinesiophobia and oral health-related quality of life in patients with temporomandibular disorders, study protocol for a randomized controlled trial. Medicine (Baltimore). 2020 Nov 20;99(47):e23295. doi: 10.1097/MD.0000000000023295. PMID: 33217860; PMCID: PMC7676583. 2. Ramsay CR, Clarkson JE, Duncan A, Lamont TJ, Heasman PA, Boyers D, Goulão B, Bonetti D, Bruce R, Gouick J, Heasman L, Lovelock-Hempleman LA, Macpherson LE, McCracken GI, McDonald AM, McLaren-Neil F, Mitchell FE, Norrie JD, van der Pol M, Sim K, Steele JG, Sharp A, Watt G, Worthington HV, Young L. Improving the Quality of Dentistry (IQuaD): a cluster factorial randomised controlled trial comparing the effectiveness and cost-benefit of oral hygiene advice and/or periodontal instrumentation with routine care for the prevention and management of periodontal disease in dentate adults attending dental primary care. Health Technol Assess. 2018 Jul;22(38):1-144. doi: 10.3310/hta22380. PMID: 29984691; PMCID: PMC6055082. 3. Ghaffari M, Rakhshanderou S, Ramezankhani A, Buunk-Werkhoven Y, Noroozi M, Armoon B. Are educating and promoting interventions effective in oral health?: A systematic review. Int J Dent Hyg. 2018 Feb;16(1):48-58. doi: 10.1111/idh.12305. Epub 2017 Aug 22. PMID: 28834249. 4. Divdar M, Araban M, Heydarabadi AB, Cheraghian B, Stein LAR. Effectiveness of message-framing to improve oral health behaviors and dental plaque among pregnant women. Arch Public Health. 2021 Jun 28;79(1):117. doi: 10.1186/s13690-021-00640-1. PMID: 34183061; PMCID: PMC8237427. 5. Aguirre PEA, Lotto M, Strieder AP, Cruvinel AFP, Cruvinel T. The Effectiveness of Educational Mobile Messages for Assisting in the Prevention of Early Childhood Caries: Protocol for a Randomized Controlled Trial. JMIR Res Protoc. 2019 Sep 3;8(9):e13656. doi: 10.2196/13656. PMID: 31482856; PMCID: PMC6751091. |
| Hygiene, prevention of infectious diseases (and their spreading),  Covid-19, | 1. Zhang L, Xu Y, Jin X, Shi Z, Wu M, Xu N, Yu X, Deng S, Zhang K, Zhang L, Hannig M, Chen Q, Fu B. Retrospective study on the effectiveness of a prevention strategy in a dental hospital during the COVID-19 pandemic. Clin Oral Investig. 2021 Oct;25(10):5815-5822. doi: 10.1007/s00784-021-03886-9. Epub 2021 Mar 18. PMID: 33738652; PMCID: PMC7972332. 2. Samaranayake LP, Fakhruddin KS, Ngo HC, Chang JWW, Panduwawala C. The effectiveness and efficacy of respiratory protective equipment (RPE) in dentistry and other health care settings: a systematic review. Acta Odontol Scand. 2020 Nov;78(8):626-639. doi: 10.1080/00016357.2020.1810769. Epub 2020 Sep 3. PMID: 32881590. 3. Offner D, Fernandez De Grado G, Strub M, Belotti L, Deboscker S, Musset AM. Mobile Dental Delivery System: An Effective Protocol for Hygiene and Disinfection. Int J Environ Res Public Health. 2020 Mar 2;17(5):1603. doi: 10.3390/ijerph17051603. PMID: 32131416; PMCID: PMC7084903. |
| oral hygiene / health knowledge | 1. Yeo KY, Hashimoto K, Archer T, Kenny K, Pavitt S, Zoltie T. Evaluation on the effectiveness of a peer led video on oral hygiene education in young children. J Vis Commun Med. 2020 Jul;43(3):119-127. doi: 10.1080/17453054.2020.1782728. Epub 2020 Jul 6. PMID: 32623921. 2. Verlinden DA, Schuller AA, Verrips GHW, Reijneveld SA. Effectiveness of a short web-based film targeting parental oral health knowledge in a well-child care setting. Eur J Oral Sci. 2020 Jun;128(3):226-232. doi: 10.1111/eos.12700. Epub 2020 May 12. PMID: 32396668; PMCID: PMC7317767. 3. Subedi K, Shrestha A, Bhagat T, Baral D. Effectiveness of oral health education intervention among 12-15-year-old school children in Dharan, Nepal: a randomized controlled trial. BMC Oral Health. 2021 Oct 14;21(1):525. doi: 10.1186/s12903-021-01877-6. PMID: 34649553; PMCID: PMC8515708. 4. Sfeatcu R, Dumitrache MA, Cărămidă M, Johannsen A, Perlea P. A pilot study on the effectiveness of a 2-year school-based oral health educational programme using experiential learning among adolescents. Int J Dent Hyg. 2019 Aug;17(3):221-228. doi: 10.1111/idh.12400. Epub 2019 Apr 25. PMID: 30945426. 5. Nayak PP, Nayak SS, Sathiyabalan D, Aditya NK, Das P. Assessing the Feasibility and Effectiveness of an App in Improving Knowledge on Oral Cancer-an Interventional Study. J Cancer Educ. 2018 Dec;33(6):1250-1254. doi: 10.1007/s13187-017-1239-y. PMID: 28612324. 6. Liu HY, Chen PH, Chen WJ, Huang SS, Chen JH, Yao CT. The Effectiveness of a Board Game-Based Oral Hygiene Education Program on Oral Hygiene Knowledge and Plaque Index of Adults with Intellectual Disability: A Pilot Study. Int J Environ Res Public Health. 2021 Jan 22;18(3):946. doi: 10.3390/ijerph18030946. PMID: 33499076; PMCID: PMC7908306. 7. Kuo MW, Yeh SH, Chang HM, Teng PR. Effectiveness of oral health promotion program for persons with severe mental illness: a cluster randomized controlled study. BMC Oral Health. 2020 Oct 27;20(1):290. doi: 10.1186/s12903-020-01280-7. PMID: 33109148; PMCID: PMC7590455. 8. Kılınç G, Açıkgöz A, Koca B, Toyran N, Güldaş N. An evaluation of the effectiveness of dental health training administered at a health services school. Health Promot J Austr. 2021 Apr;32(2):295-302. doi: 10.1002/hpja.353. Epub 2020 May 11. PMID: 32337756. 9. Ghaffari M, Rakhshanderou S, Ramezankhani A, Buunk-Werkhoven Y, Noroozi M, Armoon B. Are educating and promoting interventions effective in oral health?: A systematic review. Int J Dent Hyg. 2018 Feb;16(1):48-58. doi: 10.1111/idh.12305. Epub 2017 Aug 22. PMID: 28834249. 10. Geetha Priya PR, Asokan S, Janani RG, Kandaswamy D. Effectiveness of school dental health education on the oral health status and knowledge of children: A systematic review. Indian J Dent Res. 2019 May-Jun;30(3):437-449. doi: 10.4103/ijdr.IJDR_805_18. PMID: 31397422. 11. Duruk G, Gümüşboğa ZŞ. Effectiveness of the ToothSOS App as a training tool for the emergency management of traumatic dental injuries among non-dentists. Dent Traumatol. 2022 Jun;38(3):229-237. doi: 10.1111/edt.12742. Epub 2022 Mar 11. PMID: 35276026. 12. Divdar M, Araban M, Heydarabadi AB, Cheraghian B, Stein LAR. Effectiveness of message-framing to improve oral health behaviors and dental plaque among pregnant women. Arch Public Health. 2021 Jun 28;79(1):117. doi: 10.1186/s13690-021-00640-1. PMID: 34183061; PMCID: PMC8237427. 13. Deolia S, Johny J, Patil MS, Lanje NR, Patil AV. Effectiveness of "Audio-tactile Performance Technique" to improve the oral hygiene status of visually impaired schoolchildren. J Indian Soc Pedod Prev Dent. 2019 Apr-Jun;37(2):172-176. doi: 10.4103/1319-2442.261344. PMID: 31249182. 14. Bramantoro T, Santoso CMA, Hariyani N, Setyowati D, Zulfiana AA, Nor NAM, Nagy A, Pratamawari DNP, Irmalia WR. Effectiveness of the school-based oral health promotion programmes from preschool to high school: A systematic review. PLoS One. 2021 Aug 11;16(8):e0256007. doi: 10.1371/journal.pone.0256007. PMID: 34379685; PMCID: PMC8357156. 15. Alrashdi M, Hameed A, Cervantes Mendez MJ, Farokhi M. Education intervention with respect to the oral health knowledge, attitude, and behaviors of refugee families: A randomized clinical trial of effectiveness. J Public Health Dent. 2021 Jun;81(2):90-99. doi: 10.1111/jphd.12415. Epub 2020 Oct 20. PMID: 33084019; PMCID: PMC8246856. 16. Agarwal D, Kumar A, B C M, Sethi S, Yadav V, Shyam R, Kumar V. Effectiveness of oral health education on oral hygiene status among schizophrenic patients: A randomized controlled study. Spec Care Dentist. 2019 May;39(3):255-261. doi: 10.1111/scd.12373. Epub 2019 Mar 12. PMID: 30861167. |
| Oral health behavior (also of caregivers); psychosocial factors of the HAPA model; oral hygiene (plaque, gingival bleeding, brushing time, brushing technique, brushing frequency, brushing time, etc.), use of fluoridated toothpaste, oral health perception, pneumonia, oral health attitude, oral microbiome | 1. Xiang B, Wong HM, Perfecto AP, McGrath CPJ. The effectiveness of behavioral interventions to improve oral health in adolescents at different periods of follow-up: A systematic review and meta-analysis. Patient Educ Couns. 2020 Apr;103(4):725-733. doi: 10.1016/j.pec.2019.11.030. Epub 2019 Dec 3. PMID: 31813713. 2. Wu W, Hu L, Chen Y, Cao F, Ding S, Wu T, Xu J. Effectiveness of an online application of the health action process approach (HAPA) theory on oral hygiene intervention in young adults with fixed orthodontic appliances: a randomized controlled trial. BMC Oral Health. 2022 May 19;22(1):192. doi: 10.1186/s12903-022-02219-w. PMID: 35590291; PMCID: PMC9118762. 3. Wiatrak K, Morawiec T, Rój R, Kownacki P, Nitecka-Buchta A, Niedzielski D, Wychowański P, Machorowska-Pieniążek A, Cholewka A, Baldi D, Mertas A. Evaluation of Effectiveness of a Toothpaste Containing Tea Tree Oil and Ethanolic Extract of Propolis on the Improvement of Oral Health in Patients Using Removable Partial Dentures. Molecules. 2021 Jul 3;26(13):4071. doi: 10.3390/molecules26134071. PMID: 34279411; PMCID: PMC8271704. 4. Wang P, Xu Y, Zhang J, Chen X, Liang W, Liu X, Xian J, Xie H. Comparison of the effectiveness between power toothbrushes and manual toothbrushes for oral health: a systematic review and meta-analysis. Acta Odontol Scand. 2020 May;78(4):265-274. doi: 10.1080/00016357.2019.1697826. Epub 2019 Dec 9. PMID: 32285744. 5. Vpk V, Mohanty VR, Balappanavar AY, Juneja M, Gupta V, Kapoor S. Effectiveness of different parenting interventions on oral hygiene of cerebral palsy children: A randomized controlled trial. Spec Care Dentist. 2020 Jul;40(4):335-343. doi: 10.1111/scd.12481. Epub 2020 Jun 2. PMID: 32488889. 6. Tiwari BS, Ankola AV, Jalihal S, Patil P, Sankeshwari RM, Kashyap BR. Effectiveness of different oral health education interventions in visually impaired school children. Spec Care Dentist. 2019 Mar;39(2):97-107. doi: 10.1111/scd.12356. Epub 2019 Jan 18. PMID: 30657191. 7. Tidke S, Chhabra GK, Madhu PP, Reche A, Wazurkar S, Singi SR. The Effectiveness of Herbal Versus Non-Herbal Mouthwash for Periodontal Health: A Literature Review. Cureus. 2022 Aug 13;14(8):e27956. doi: 10.7759/cureus.27956. PMID: 36120261; PMCID: PMC9465625. 8. Subedi K, Shrestha A, Bhagat T, Baral D. Effectiveness of oral health education intervention among 12-15-year-old school children in Dharan, Nepal: a randomized controlled trial. BMC Oral Health. 2021 Oct 14;21(1):525. doi: 10.1186/s12903-021-01877-6. PMID: 34649553; PMCID: PMC8515708. 9. Subburaman N, Madan Kumar PD, Iyer K. Effectiveness of musical toothbrush on oral debris and gingival bleeding among 6-10-year-old children: A randomized controlled trial. Indian J Dent Res. 2019 Mar-Apr;30(2):196-199. doi: 10.4103/ijdr.IJDR_128_17. PMID: 31169149. 10. Stein C, Santos NML, Hilgert JB, Hugo FN. Effectiveness of oral health education on oral hygiene and dental caries in schoolchildren: Systematic review and meta-analysis. Community Dent Oral Epidemiol. 2018 Feb;46(1):30-37. doi: 10.1111/cdoe.12325. Epub 2017 Aug 16. PMID: 28815661. 11. Sivertsen TB, Åstrøm AN, Greve G, Aßmus J, Skeie MS. Effectiveness of an oral health intervention program for children with congenital heart defects. BMC Oral Health. 2018 Mar 23;18(1):50. doi: 10.1186/s12903-018-0495-5. PMID: 29566698; PMCID: PMC5865357. 12. Singh M, Papas A, Gerlach RW. Safety and effectiveness of a two-step dentifrice/gel sequence with medication-associated hyposalivation: A randomized controlled trial in a vulnerable population. Am J Dent. 2018 Jul;31(Sp Is A):24A-28A. PMID: 30106530. 13. Shakir A, Barngkgei I, Godson J, Joury E. Effectiveness of school-based behavioural interventions to improve children's oral health by reducing sugar intake and promoting oral hygiene: A rapid review of randomised controlled trials. Community Dent Health. 2021 Nov 29;38(4):275-283. doi: 10.1922/CDH_00014Shakir09. PMID: 34351713. 14. Sfeatcu R, Dumitrache MA, Cărămidă M, Johannsen A, Perlea P. A pilot study on the effectiveness of a 2-year school-based oral health educational programme using experiential learning among adolescents. Int J Dent Hyg. 2019 Aug;17(3):221-228. doi: 10.1111/idh.12400. Epub 2019 Apr 25. PMID: 30945426. 15. Sermsuti-Anuwat N, Pongpanich S. Effectiveness of an individually tailored oral hygiene intervention in improving gingival health among community-dwelling adults with physical disabilities in Thailand. Spec Care Dentist. 2021 Mar;41(2):202-209. doi: 10.1111/scd.12554. Epub 2020 Dec 22. PMID: 33350502. 16. Schulz-Weidner N, Bulski JC, Siahi-Benlarbi R, Bauer J, Logeswaran T, Jux C, Wetzel WE, Krämer N. Symptom-orientated oral hygiene for children after heart transplantation: Effectiveness of a standardized prophylactic program. Pediatr Transplant. 2020 Nov;24(7):e13845. doi: 10.1111/petr.13845. Epub 2020 Sep 30. PMID: 32997421. 17. Satyarup D, Dalai RP, Nagarajappa R, Naik D, Mohanty I. Effectiveness of trained health workers in improving the oral hygiene of preschool children. Rocz Panstw Zakl Hig. 2021;72(1):77-82. doi: 10.32394/rpzh.2021.0148. PMID: 33883064. 18. Ramsay CR, Clarkson JE, Duncan A, Lamont TJ, Heasman PA, Boyers D, Goulão B, Bonetti D, Bruce R, Gouick J, Heasman L, Lovelock-Hempleman LA, Macpherson LE, McCracken GI, McDonald AM, McLaren-Neil F, Mitchell FE, Norrie JD, van der Pol M, Sim K, Steele JG, Sharp A, Watt G, Worthington HV, Young L. Improving the Quality of Dentistry (IQuaD): a cluster factorial randomised controlled trial comparing the effectiveness and cost-benefit of oral hygiene advice and/or periodontal instrumentation with routine care for the prevention and management of periodontal disease in dentate adults attending dental primary care. Health Technol Assess. 2018 Jul;22(38):1-144. doi: 10.3310/hta22380. PMID: 29984691; PMCID: PMC6055082. 19. Ramli H, Nor Aripin KN, Mohd Said S, Mohamad Hanafiah R, Mohd Dom TN. The effectiveness of miswak (Salvadora persica L. and Azadirachta indica A.Juss.) practices in reducing plaque and gingivitis among adults: A systematic review and meta-analysis. J Ethnopharmacol. 2022 Nov 15;298:115598. doi: 10.1016/j.jep.2022.115598. Epub 2022 Aug 6. PMID: 35944735. 20. Rajwani AR, Hawes SND, To A, Quaranta A, Rincon Aguilar JC. Effectiveness of Manual Toothbrushing Techniques on Plaque and Gingivitis: A Systematic Review. Oral Health Prev Dent. 2020 Oct 2;18(1):843-854. doi: 10.3290/j.ohpd.a45354. PMID: 33028052. 21. Rajab LD, Assaf DH, El-Smadi LA, Hamdan AA. Comparison of effectiveness of oral hygiene instruction methods in improving plaque scores among 8-9-year children: a randomized controlled trial. Eur Arch Paediatr Dent. 2022 Apr;23(2):289-300. doi: 10.1007/s40368-021-00680-9. Epub 2021 Nov 15. PMID: 34780032. 22. Rai T, Ym K, Rao A, P AN, Natarajan S, Joseph RM. Evaluation of the effectiveness of a custom-made toothbrush in maintaining oral hygiene and gingival health in cerebral palsy patients. Spec Care Dentist. 2018 Nov;38(6):367-372. doi: 10.1111/scd.12334. Epub 2018 Oct 23. PMID: 30350870. 23. Rabello F, Araújo VE, Magalhães S. Effectiveness of oral chlorhexidine for the prevention of nosocomial pneumonia and ventilator-associated pneumonia in intensive care units: Overview of systematic reviews. Int J Dent Hyg. 2018 Nov;16(4):441-449. doi: 10.1111/idh.12336. Epub 2018 Feb 23. PMID: 29473687. 24. Wolfensberger A, Clack L, von Felten S, Kusejko K, Faes Hesse M, Jakob W, Saleschus D, Meier MT, Kouyos R, Held L, Sax H. Implementation and evaluation of a care bundle for prevention of non-ventilator-associated hospital-acquired pneumonia (nvHAP) - a mixed-methods study protocol for a hybrid type 2 effectiveness-implementation trial. BMC Infect Dis. 2020 Aug 17;20(1):603. doi: 10.1186/s12879-020-05271-5. Erratum in: BMC Infect Dis. 2021 Jan 25;21(1):115. PMID: 32807090; PMCID: PMC7429945. 25. Wu J, Dai Y, Lo ECM, Qi Y, Zhang Y, Li QL, Dai R. Using metagenomic analysis to assess the effectiveness of oral health promotion interventions in reducing risk for pneumonia among patients with stroke in acute phase: study protocol for a randomized controlled trial. Trials. 2020 Jul 10;21(1):634. doi: 10.1186/s13063-020-04528-3. PMID: 32650814; PMCID: PMC7350693. 26. Sankaran SP, Sonis S. Network meta-analysis from a pairwise meta-analysis design: to assess the comparative effectiveness of oral care interventions in preventing ventilator-associated pneumonia in critically ill patients. Clin Oral Investig. 2021 May;25(5):2439-2447. doi: 10.1007/s00784-021-03802-1. Epub 2021 Feb 3. PMID: 33537946. 27. Petker-Jung W, Weik U, Margraf-Stiksrud J, Deinzer R. What characterizes effective tooth brushing of daily users of powered versus manual toothbrushes? BMC Oral Health. 2022 Jan 16;22(1):10. doi: 10.1186/s12903-022-02045-0. PMID: 35034614; PMCID: PMC8762860. 28. Patil S, Hedad IA, Jafer AA, Abutaleb GK, Arishi TM, Arishi SA, Arishi HA, Jafer M, Gujar AN, Khan S, Raj AT. Effectiveness of mobile phone applications in improving oral hygiene care and outcomes in orthodontic patients. J Oral Biol Craniofac Res. 2021 Jan-Mar;11(1):26-32. doi: 10.1016/j.jobcr.2020.11.004. Epub 2020 Nov 17. PMID: 33344158; PMCID: PMC7736984. 29. Ng E, Lim LP. An Overview of Different Interdental Cleaning Aids and Their Effectiveness. Dent J (Basel). 2019 Jun 1;7(2):56. doi: 10.3390/dj7020056. PMID: 31159354; PMCID: PMC6630384. 30. Muric A. The Effectiveness of Reform in the Dental Health Systems of Transitional Countries: The Case of Montenegro Health Reform (pilot study). Community Dent Health. 2019 May 30;36(2):126-130. doi: 10.1922/CDH_4435Muric05. PMID: 31021565. 31. Milleman J, Bosma ML, McGuire JA, Sunkara A, McAdoo K, DelSasso A, Wills K, Milleman K. Comparative Effectiveness of Toothbrushing, Flossing and Mouthrinse Regimens on Plaque and Gingivitis: A 12-week virtually supervised clinical trial. J Dent Hyg. 2022 Jun;96(3):21-34. PMID: 35654564. 32. Melo P, Fine C, Malone S, Frencken JE, Horn V. The effectiveness of the Brush Day and Night programme in improving children's toothbrushing knowledge and behaviour. Int Dent J. 2018 May;68 Suppl 1(Suppl 1):7-16. doi: 10.1111/idj.12410. Epub 2018 Apr 16. PMID: 29660791; PMCID: PMC9379073. 33. McGowan LJ, Joyes EC, Adams EA, Coyte A, Gavin R, Richmond C, Shabaninejad H, Beyer F, Broadbridge A, Dobson K, Landes D, Moffatt S, Watt RG, Sniehotta FF, Freeman R, Paisi M, Bambra C, Craig D, Kaner E, Ramsay SE. Investigating the Effectiveness and Acceptability of Oral Health and Related Health Behaviour Interventions in Adults with Severe and Multiple Disadvantage: Protocol for a Mixed-Methods Systematic Review. Int J Environ Res Public Health. 2021 Nov 3;18(21):11554. doi: 10.3390/ijerph182111554. PMID: 34770066; PMCID: PMC8582803. 34. Liu P, Wen W, Yu KF, Gao X, Lo ECM, Wong MCM. Effectiveness of a family-centered behavioral and educational counselling approach to improve periodontal health of pregnant women: a randomized controlled trial. BMC Oral Health. 2020 Oct 16;20(1):284. doi: 10.1186/s12903-020-01265-6. PMID: 33066773; PMCID: PMC7568358. 35. Liu HY, Chen PH, Chen WJ, Huang SS, Chen JH, Yao CT. The Effectiveness of a Board Game-Based Oral Hygiene Education Program on Oral Hygiene Knowledge and Plaque Index of Adults with Intellectual Disability: A Pilot Study. Int J Environ Res Public Health. 2021 Jan 22;18(3):946. doi: 10.3390/ijerph18030946. PMID: 33499076; PMCID: PMC7908306. 36. Le Fouler A, Jeanne S, Sorel O, Brézulier D. How effective are three methods of teaching oral hygiene for adolescents undergoing orthodontic treatment? The MAHO protocol: an RCT comparing visual, auditory and kinesthetic methods. Trials. 2021 Feb 15;22(1):144. doi: 10.1186/s13063-021-05093-z. PMID: 33588930; PMCID: PMC7885203. 37. Kuwamura Y, Yoshida S, Kurahash K, Sumikawa M, Yumoto H, Uemura H, Matsuhisa M. Effectiveness of a Diabetes Oral Nursing Program Including a Modified Diabetes Oral Health Assessment Tool for Nurses (M-DiOHAT©) : A 12-Month Follow-Up Intervention Study. J Med Invest. 2022;69(1.2):86-96. doi: 10.2152/jmi.69.86. PMID: 35466152. 38. Kuo MW, Yeh SH, Chang HM, Teng PR. Effectiveness of oral health promotion program for persons with severe mental illness: a cluster randomized controlled study. BMC Oral Health. 2020 Oct 27;20(1):290. doi: 10.1186/s12903-020-01280-7. PMID: 33109148; PMCID: PMC7590455. 39. Krishnan L, Iyer K, Kumar PDM. Effectiveness of two sensory-based health education methods on oral hygiene of adolescent with autism spectrum disorders: An interventional study. Spec Care Dentist. 2021 Sep;41(5):626-633. doi: 10.1111/scd.12606. Epub 2021 May 29. PMID: 34050975. 40. Kim YW, Lee J, Lee TH, Lim S. Bioelectric effect utilized a healthcare device for effective management of dental biofilms and gingivitis. Med Eng Phys. 2022 Jun;104:103804. doi: 10.1016/j.medengphy.2022.103804. Epub 2022 Apr 17. PMID: 35641073. 41. Kılınç G, Açıkgöz A, Koca B, Toyran N, Güldaş N. An evaluation of the effectiveness of dental health training administered at a health services school. Health Promot J Austr. 2021 Apr;32(2):295-302. doi: 10.1002/hpja.353. Epub 2020 May 11. PMID: 32337756. 42. Khurana C, Tandon S, Chinmaya BR. A crossover clinical trial to assess the effectiveness of different oral hygiene regimens on the reduction of morning bad breath in healthy young adults. Indian J Dent Res. 2018 Jul-Aug;29(4):434-439. doi: 10.4103/ijdr.IJDR_800_16. PMID: 30127192. 43. Keyong E, Thitasomakul S, Tianviwat S. Effectiveness of an Oral Health Promotion Program for the Elderly in Khiri Mat District, Sukhothai Province: A Randomized Control Trial. J Int Soc Prev Community Dent. 2019 May-Jun;9(3):225-231. doi: 10.4103/jispcd.JISPCD_27_19. Epub 2019 Jun 7. PMID: 31198693; PMCID: PMC6559037. 44. Janakiram C, Venkitachalam R, Fontelo P, Iafolla TJ, Dye BA. Effectiveness of herbal oral care products in reducing dental plaque & gingivitis - a systematic review and meta-analysis. BMC Complement Med Ther. 2020 Feb 11;20(1):43. doi: 10.1186/s12906-020-2812-1. PMID: 32046707; PMCID: PMC7076867. 45. Ho BV, Weijenberg RAF, van der Maarel-Wierink CD, Visscher CM, van der Putten GJ, Scherder EJA, Lobbezoo F. Effectiveness of the implementation project 'Don't forget the mouth!' of community dwelling older people with dementia: a prospective longitudinal single-blind multicentre study protocol (DFTM!). BMC Oral Health. 2019 May 28;19(1):91. doi: 10.1186/s12903-019-0786-5. PMID: 31138273; PMCID: PMC6537198. 46. Hashmi S, Mohanty VR, Balappanavar AY, Yadav V, Kapoor S, Rijhwani K. Effectiveness of dental health education on oral hygiene among hearing impaired adolescents in India: A randomized control trial. Spec Care Dentist. 2019 May;39(3):274-280. doi: 10.1111/scd.12374. Epub 2019 Apr 9. PMID: 30964562. 47. Ghaffari M, Rakhshanderou S, Ramezankhani A, Buunk-Werkhoven Y, Noroozi M, Armoon B. Are educating and promoting interventions effective in oral health?: A systematic review. Int J Dent Hyg. 2018 Feb;16(1):48-58. doi: 10.1111/idh.12305. Epub 2017 Aug 22. PMID: 28834249. 48. George A, Sousa MS, Kong AC, Blinkhorn A, Patterson Norrie T, Foster J, Dahlen HG, Ajwani S, Johnson M. Effectiveness of preventive dental programs offered to mothers by non-dental professionals to control early childhood dental caries: a review. BMC Oral Health. 2019 Aug 2;19(1):172. doi: 10.1186/s12903-019-0862-x. PMID: 31375106; PMCID: PMC6679429. 49. Geetha Priya PR, Asokan S, Janani RG, Kandaswamy D. Effectiveness of school dental health education on the oral health status and knowledge of children: A systematic review. Indian J Dent Res. 2019 May-Jun;30(3):437-449. doi: 10.4103/ijdr.IJDR_805_18. PMID: 31397422. 50. Faisal MR, Mishu MP, Jahangir F, Younes S, Dogar O, Siddiqi K, Torgerson DJ. The effectiveness of behaviour change interventions delivered by non-dental health workers in promoting children's oral health: A systematic review and meta-analysis. PLoS One. 2022 Jan 11;17(1):e0262118. doi: 10.1371/journal.pone.0262118. PMID: 35015771; PMCID: PMC8751985. 51. Divdar M, Araban M, Heydarabadi AB, Cheraghian B, Stein LAR. Effectiveness of message-framing to improve oral health behaviors and dental plaque among pregnant women. Arch Public Health. 2021 Jun 28;79(1):117. doi: 10.1186/s13690-021-00640-1. PMID: 34183061; PMCID: PMC8237427. 52. Deolia S, Johny J, Patil MS, Lanje NR, Patil AV. Effectiveness of "Audio-tactile Performance Technique" to improve the oral hygiene status of visually impaired schoolchildren. J Indian Soc Pedod Prev Dent. 2019 Apr-Jun;37(2):172-176. doi: 10.4103/1319-2442.261344. PMID: 31249182. 53. Das D, Suresan V, Jnaneswar A, Pathi J, Bala Subramaniam G. Effectiveness of a novel oral health education technique in maintenance of gingival health and plaque removal efficacy among institutionalized visually impaired children of Bhubaneswar city: A randomized controlled trial. Spec Care Dentist. 2019 Mar;39(2):125-134. doi: 10.1111/scd.12350. Epub 2018 Dec 30. PMID: 30597580. 54. Chua H, Sardana D, Turner R, Ting G, Ekambaram M. Effectiveness of oral health education methods on oral hygiene in children and adolescents with visual impairment: A systematic review. Int J Paediatr Dent. 2021 Nov;31(6):724-741. doi: 10.1111/ipd.12788. Epub 2021 May 6. PMID: 33730385. 55. Cheng HC, Hu HT, Chang YC. Effectiveness of Enzyme Dentifrices on Oral Health in Orthodontic Patients: A Randomized Controlled Trial. Int J Environ Res Public Health. 2019 Jun 25;16(12):2243. doi: 10.3390/ijerph16122243. PMID: 31242669; PMCID: PMC6617311. 56. Chatzopoulos GS, Karakostas P, Kavakloglou S, Assimopoulou A, Barmpalexis P, Tsalikis L. Clinical Effectiveness of Herbal Oral Care Products in Periodontitis Patients: A Systematic Review. Int J Environ Res Public Health. 2022 Aug 15;19(16):10061. doi: 10.3390/ijerph191610061. PMID: 36011693; PMCID: PMC9408146. 57. Ceyhan D, Akdik C, Kirzioglu Z. An educational programme designed for the evaluation of effectiveness of two tooth brushing techniques in preschool children. Eur J Paediatr Dent. 2018 Sep;19(3):181-186. doi: 10.23804/ejpd.2018.19.03.3. PMID: 30063148. 58. Bramantoro T, Santoso CMA, Hariyani N, Setyowati D, Zulfiana AA, Nor NAM, Nagy A, Pratamawari DNP, Irmalia WR. Effectiveness of the school-based oral health promotion programmes from preschool to high school: A systematic review. PLoS One. 2021 Aug 11;16(8):e0256007. doi: 10.1371/journal.pone.0256007. PMID: 34379685; PMCID: PMC8357156. 59. Brady MC, Stott DJ, Weir CJ, Chalmers C, Sweeney P, Barr J, Pollock A, Bowers N, Gray H, Bain BJ, Collins M, Keerie C, Langhorne P. A pragmatic, multi-centered, stepped wedge, cluster randomized controlled trial pilot of the clinical and cost effectiveness of a complex Stroke Oral healthCare intervention pLan Evaluation II (SOCLE II) compared with usual oral healthcare in stroke wards. Int J Stroke. 2020 Apr;15(3):318-323. doi: 10.1177/1747493019871824. Epub 2019 Sep 30. PMID: 31564241; PMCID: PMC7153219. 60. Bhor KB, Vinay V, Ambildhok K, Shetty V. Effectiveness of oral health educational interventions on oral health of visually impaired school children: A systematic review and meta-analysis. Spec Care Dentist. 2021 May;41(3):291-308. doi: 10.1111/scd.12567. Epub 2021 Jan 20. PMID: 33471416. 61. Barbe AG, Al-Barwari A, Hamacher S, Deinzer R, Weik U, Noack MJ. Effectiveness of brushing teeth in patients with reduced oral hygiene by laypeople: a randomized, controlled study. BMC Oral Health. 2021 Apr 30;21(1):225. doi: 10.1186/s12903-021-01590-4. PMID: 33941170; PMCID: PMC8091671. 62. Balian A, Cirio S, Salerno C, Wolf TG, Campus G, Cagetti MG. Is Visual Pedagogy Effective in Improving Cooperation Towards Oral Hygiene and Dental Care in Children with Autism Spectrum Disorder? A Systematic Review and Meta-Analysis. Int J Environ Res Public Health. 2021 Jan 18;18(2):789. doi: 10.3390/ijerph18020789. PMID: 33477719; PMCID: PMC7832292. 63. Asaad N, Laflouf M. Effectiveness of Apple Cider Vinegar and Mechanical Removal on Dental Plaque and Gingival Inflammation of Children With Cerebral Palsy. Cureus. 2022 Jul 15;14(7):e26874. doi: 10.7759/cureus.26874. PMID: 35978745; PMCID: PMC9375849. 64. Alrashdi M, Hameed A, Cervantes Mendez MJ, Farokhi M. Education intervention with respect to the oral health knowledge, attitude, and behaviors of refugee families: A randomized clinical trial of effectiveness. J Public Health Dent. 2021 Jun;81(2):90-99. doi: 10.1111/jphd.12415. Epub 2020 Oct 20. PMID: 33084019; PMCID: PMC8246856. 65. Al-Mahmood S, Sabea DW. Comparative Evaluation of the Effectiveness of 40% Miswak Mouthwash and 0.12% Chlorhexidine Mouthwash in Treating Gingivitis: A Blinded, Randomised Clinical Trial. Oral Health Prev Dent. 2021 Jan 7;19(1):229-233. doi: 10.3290/j.ohpd.b1179501. PMID: 33829720. 66. Akera P, Kennedy SE, Lingam R, Obwolo MJ, Schutte AE, Richmond R. Effectiveness of primary school-based interventions in improving oral health of children in low- and middle-income countries: a systematic review and meta-analysis. BMC Oral Health. 2022 Jun 29;22(1):264. doi: 10.1186/s12903-022-02291-2. PMID: 35768801; PMCID: PMC9245251. 67. Aguirre PEA, Lotto M, Strieder AP, Cruvinel AFP, Cruvinel T. The Effectiveness of Educational Mobile Messages for Assisting in the Prevention of Early Childhood Caries: Protocol for a Randomized Controlled Trial. JMIR Res Protoc. 2019 Sep 3;8(9):e13656. doi: 10.2196/13656. PMID: 31482856; PMCID: PMC6751091. 68. Agarwal D, Kumar A, B C M, Sethi S, Yadav V, Shyam R, Kumar V. Effectiveness of oral health education on oral hygiene status among schizophrenic patients: A randomized controlled study. Spec Care Dentist. 2019 May;39(3):255-261. doi: 10.1111/scd.12373. Epub 2019 Mar 12. PMID: 30861167. |
| Caries (prevalence, caries increment, DMFT, DMFS, number of new / arrested carious surface, number needed to treat, prevented fraction (PF), relative risks (RRs), Total Observed Caries Experience (TOCE), prevention, untreated decay, white spot lesions, risk assessment, cavity-free-months, untreated caries) | 1. Wu S, Zhang T, Liu Q, Yu X, Zeng X. Effectiveness of fluoride varnish on caries in the first molars of primary schoolchildren: a 3-year longitudinal study in Guangxi Province, China. Int Dent J. 2020 Apr;70(2):108-115. doi: 10.1111/idj.12528. Epub 2019 Nov 8. PMID: 31705529; PMCID: PMC9379175. 2. Tickle M, Ricketts DJN, Duncan A, O'Malley L, Donaldson PM, Clarkson JE, Black M, Boyers D, Donaldson M, Floate R, Forrest MM, Fraser A, Glenny AM, Goulao B, McDonald A, Ramsay CR, Ross C, Walsh T, Worthington HV, Young L, Bonetti DL, Gouick J, Mitchell FE, Macpherson LE, Lin YL, Pretty IA, Birch S. Protocol for a Randomised controlled trial to Evaluate the effectiveness and cost benefit of prescribing high dose FLuoride toothpaste in preventing and treating dEntal Caries in high-risk older adulTs (reflect trial). BMC Oral Health. 2019 May 24;19(1):88. doi: 10.1186/s12903-019-0749-x. PMID: 31126270; PMCID: PMC6534863. 3. Subbiah GK, Gopinathan NM. Is Silver Diamine Fluoride Effective in Preventing and Arresting Caries in Elderly Adults? A Systematic Review. J Int Soc Prev Community Dent. 2018 May-Jun;8(3):191-199. doi: 10.4103/jispcd.JISPCD_99_18. Epub 2018 May 17. PMID: 29911054; PMCID: PMC5985673. 4. Spencer AJ, Do LG, Ha DH. Contemporary evidence on the effectiveness of water fluoridation in the prevention of childhood caries. Community Dent Oral Epidemiol. 2018 Aug;46(4):407-415. doi: 10.1111/cdoe.12384. Epub 2018 Jun 5. PMID: 29869803. 5. Sivertsen TB, Åstrøm AN, Greve G, Aßmus J, Skeie MS. Effectiveness of an oral health intervention program for children with congenital heart defects. BMC Oral Health. 2018 Mar 23;18(1):50. doi: 10.1186/s12903-018-0495-5. PMID: 29566698; PMCID: PMC5865357. 6. Shakir A, Barngkgei I, Godson J, Joury E. Effectiveness of school-based behavioural interventions to improve children's oral health by reducing sugar intake and promoting oral hygiene: A rapid review of randomised controlled trials. Community Dent Health. 2021 Nov 29;38(4):275-283. doi: 10.1922/CDH_00014Shakir09. PMID: 34351713. 7. Sfeatcu R, Dumitrache MA, Cărămidă M, Johannsen A, Perlea P. A pilot study on the effectiveness of a 2-year school-based oral health educational programme using experiential learning among adolescents. Int J Dent Hyg. 2019 Aug;17(3):221-228. doi: 10.1111/idh.12400. Epub 2019 Apr 25. PMID: 30945426. 8. Ruff RR. Total observed caries experience: assessing the effectiveness of community-based caries prevention. J Public Health Dent. 2018 Sep;78(4):287-290. doi: 10.1111/jphd.12284. Epub 2018 Aug 16. PMID: 30114726; PMCID: PMC6279466. 9. Ruff RR, Niederman R. Comparative effectiveness of treatments to prevent dental caries given to rural children in school-based settings: protocol for a cluster randomised controlled trial. BMJ Open. 2018 Apr 13;8(4):e022646. doi: 10.1136/bmjopen-2018-022646. PMID: 29654053; PMCID: PMC5905731. 10. Ruff RR, Niederman R. Comparative effectiveness of school-based caries prevention: a prospective cohort study. BMC Oral Health. 2018 Mar 27;18(1):53. doi: 10.1186/s12903-018-0514-6. PMID: 29587715; PMCID: PMC5872543. 11. Muñoz-Millán P, Zaror C, Espinoza-Espinoza G, Vergara-Gonzalez C, Muñoz S, Atala-Acevedo C, Martínez-Zapata MJ. Effectiveness of fluoride varnish in preventing early childhood caries in rural areas without access to fluoridated drinking water: A randomized control trial. Community Dent Oral Epidemiol. 2018 Feb;46(1):63-69. doi: 10.1111/cdoe.12330. Epub 2017 Aug 29. PMID: 28850712. 12. Lam PP, Sardana D, Lo EC, Yiu CK. FISSURE SEALANT IN A NUTSHELL. EVIDENCE-BASED META-EVALUATION OF SEALANTS' EFFECTIVENESS IN CARIES PREVENTION AND ARREST. J Evid Based Dent Pract. 2021 Sep;21(3):101587. doi: 10.1016/j.jebdp.2021.101587. Epub 2021 May 17. PMID: 34479663. 13. Jabir E, McGrade C, Quinn G, McGarry J, Nic Iomhair A, Kelly N, Srinivasan M, Watson S, McKenna GJ. Evaluating the effectiveness of fluoride varnish in preventing caries amongst Long-Term Care Facility Residents. Gerodontology. 2022 Sep;39(3):250-256. doi: 10.1111/ger.12563. Epub 2021 May 24. PMID: 34028089. 14. Gugnani N, Gugnani S. Are sealants effective in preventing caries in primary molars? Evid Based Dent. 2022 Jun;23(2):60-61. doi: 10.1038/s41432-022-0262-y. Epub 2022 Jun 24. PMID: 35750728. 15. Ghaffari M, Rakhshanderou S, Ramezankhani A, Buunk-Werkhoven Y, Noroozi M, Armoon B. Are educating and promoting interventions effective in oral health?: A systematic review. Int J Dent Hyg. 2018 Feb;16(1):48-58. doi: 10.1111/idh.12305. Epub 2017 Aug 22. PMID: 28834249. 16. George A, Sousa MS, Kong AC, Blinkhorn A, Patterson Norrie T, Foster J, Dahlen HG, Ajwani S, Johnson M. Effectiveness of preventive dental programs offered to mothers by non-dental professionals to control early childhood dental caries: a review. BMC Oral Health. 2019 Aug 2;19(1):172. doi: 10.1186/s12903-019-0862-x. PMID: 31375106; PMCID: PMC6679429. 17. Fraihat N, Madae'en S, Bencze Z, Herczeg A, Varga O. Clinical Effectiveness and Cost-Effectiveness of Oral-Health Promotion in Dental Caries Prevention among Children: Systematic Review and Meta-Analysis. Int J Environ Res Public Health. 2019 Jul 25;16(15):2668. doi: 10.3390/ijerph16152668. PMID: 31349691; PMCID: PMC6696287. 18. Effenberger S, Greenwall L, Cebula M, Myburgh N, Simpson K, Smit D, Wicht MJ, Schwendicke F. Cost-effectiveness and efficacy of fluoride varnish for caries prevention in South African children: A cluster-randomized controlled community trial. Community Dent Oral Epidemiol. 2022 Oct;50(5):453-460. doi: 10.1111/cdoe.12702. Epub 2021 Oct 21. PMID: 34676577. 19. Cheng HC, Hu HT, Chang YC. Effectiveness of Enzyme Dentifrices on Oral Health in Orthodontic Patients: A Randomized Controlled Trial. Int J Environ Res Public Health. 2019 Jun 25;16(12):2243. doi: 10.3390/ijerph16122243. PMID: 31242669; PMCID: PMC6617311. 20. Ceyhan D, Akdik C, Kirzioglu Z. An educational programme designed for the evaluation of effectiveness of two tooth brushing techniques in preschool children. Eur J Paediatr Dent. 2018 Sep;19(3):181-186. doi: 10.23804/ejpd.2018.19.03.3. PMID: 30063148. 21. Cagetti MG, Bontà G, Cocco F, Lingstrom P, Strohmenger L, Campus G. Are standardized caries risk assessment models effective in assessing actual caries status and future caries increment? A systematic review. BMC Oral Health. 2018 Jul 16;18(1):123. doi: 10.1186/s12903-018-0585-4. PMID: 30012136; PMCID: PMC6048716. 22. Bergström EK, Davidson T, Moberg Sköld U. Cost-Effectiveness through the Dental-Health FRAMM Guideline for Caries Prevention among 12- to 15-Year-Olds in Sweden. Caries Res. 2019;53(3):339-346. doi: 10.1159/000495360. Epub 2019 Jan 16. PMID: 30650426. 23. Bakdach WMM, Hadad R. Effectiveness of different adjunctive interventions in the management of orthodontically induced white spot lesions: A systematic review of systematic reviews and meta-analyses. Dent Med Probl. 2020 Jul-Sep;57(3):305-325. doi: 10.17219/dmp/118330. PMID: 33064375. 24. Anopa Y, Conway DI. Exploring the cost-effectiveness of child dental caries prevention programmes. Are we comparing apples and oranges? Evid Based Dent. 2020 Mar;21(1):5-7. doi: 10.1038/s41432-020-0085-7. PMID: 32221482. 25. Alayadi H, Bernabé E, Sabbah W. Effectiveness of School Dental Screening to Reduce Untreated Caries: A Cluster-Randomized Controlled Trial. Caries Res. 2021;55(6):577-584. doi: 10.1159/000519770. Epub 2021 Sep 22. PMID: 34551412. 26. Alayadi H, Sabbah W, Bernabé E. Effectiveness of school dental screening on dental visits and untreated caries among primary schoolchildren: study protocol for a cluster randomised controlled trial. Trials. 2018 Apr 13;19(1):224. doi: 10.1186/s13063-018-2619-2. PMID: 29653545; PMCID: PMC5899334. 27. Akera P, Kennedy SE, Lingam R, Obwolo MJ, Schutte AE, Richmond R. Effectiveness of primary school-based interventions in improving oral health of children in low- and middle-income countries: a systematic review and meta-analysis. BMC Oral Health. 2022 Jun 29;22(1):264. doi: 10.1186/s12903-022-02291-2. PMID: 35768801; PMCID: PMC9245251. 28. Ahn E, Kim SM. The cost- effectiveness of early dental visit in infants and toddlers focused on regional deprivation in South Korea: A retrospective cohort study. PLoS One. 2022 Jun 13;17(6):e0269770. doi: 10.1371/journal.pone.0269770. PMID: 35696402; PMCID: PMC9191701. 29. Aguirre PEA, Lotto M, Strieder AP, Cruvinel AFP, Cruvinel T. The Effectiveness of Educational Mobile Messages for Assisting in the Prevention of Early Childhood Caries: Protocol for a Randomized Controlled Trial. JMIR Res Protoc. 2019 Sep 3;8(9):e13656. doi: 10.2196/13656. PMID: 31482856; PMCID: PMC6751091. 30. Water fluoridation safe and effective. Br Dent J. 2018 May 11;224(9):674. doi: 10.1038/sj.bdj.2018.377. PMID: 29747180. |
| Tooth Survival / restoration survival (rate) | 1. Widbiller M, Knüttel H, Meschi N, Durán-Sindreu Terol F. Effectiveness of endodontic tissue engineering in treatment of apical periodontitis: A systematic review. Int Endod J. 2022 Jun 14. doi: 10.1111/iej.13784. Epub ahead of print. PMID: 35699668. 2. Meschi N, Palma PJ, Cabanillas-Balsera D. Effectiveness of revitalization in treating apical periodontitis: A systematic review and meta-analysis. Int Endod J. 2022 May 17. doi: 10.1111/iej.13778. Epub ahead of print. PMID: 35579093. 3. McLister C, Donnelly M, Cardwell C, Moore C, McKenna G. Effectiveness of prosthodontic interventions and survival of remaining teeth in adult patients with shortened dental arches-Protocol for a systematic review and meta-analysis. BDJ Open. 2018 Apr 27;4:17023. doi: 10.1038/bdjopen.2017.23. PMID: 30637120; PMCID: PMC5921535. 4. McLister C, Donnelly M, Cardwell CR, Moore C, O'Neill C, Brocklehurst P, McKenna G. Effectiveness of prosthodontic interventions and survival of remaining teeth in adult patients with shortened dental arches-A systematic review. J Dent. 2018 Nov;78:31-39. doi: 10.1016/j.jdent.2018.02.003. Epub 2018 Feb 21. PMID: 29476794. 5. Duncan HF, Nagendrababu V, El-Karim I, Dummer PMH. Outcome measures to assess the effectiveness of endodontic treatment for pulpitis and apical periodontitis for use in the development of European Society of Endodontology S3-level clinical practice guidelines: A consensus-based development. Int Endod J. 2021 Dec;54(12):2184-2194. doi: 10.1111/iej.13627. Epub 2021 Sep 22. PMID: 34553383. |
| Signs of illness (swelling, tenderness, radiographic evidence, | 1. Widbiller M, Knüttel H, Meschi N, Durán-Sindreu Terol F. Effectiveness of endodontic tissue engineering in treatment of apical periodontitis: A systematic review. Int Endod J. 2022 Jun 14. doi: 10.1111/iej.13784. Epub ahead of print. PMID: 35699668. 2. Pirani C, Camilleri J. Effectiveness of root canal filling materials and techniques for treatment of apical periodontitis: A systematic review. Int Endod J. 2022 Jun 23. doi: 10.1111/iej.13787. Epub ahead of print. PMID: 35735776. 3. Duncan HF, Nagendrababu V, El-Karim I, Dummer PMH. Outcome measures to assess the effectiveness of endodontic treatment for pulpitis and apical periodontitis for use in the development of European Society of Endodontology S3-level clinical practice guidelines: A consensus-based development. Int Endod J. 2021 Dec;54(12):2184-2194. doi: 10.1111/iej.13627. Epub 2021 Sep 22. PMID: 34553383. |
| need for medication | 1. Widbiller M, Knüttel H, Meschi N, Durán-Sindreu Terol F. Effectiveness of endodontic tissue engineering in treatment of apical periodontitis: A systematic review. Int Endod J. 2022 Jun 14. doi: 10.1111/iej.13784. Epub ahead of print. PMID: 35699668. |
| Function, sensation | 1. Widbiller M, Knüttel H, Meschi N, Durán-Sindreu Terol F. Effectiveness of endodontic tissue engineering in treatment of apical periodontitis: A systematic review. Int Endod J. 2022 Jun 14. doi: 10.1111/iej.13784. Epub ahead of print. PMID: 35699668. 2. Ting CC, Ko EC, Chen CC, Chang WY, Tu HP, Chang CS. Effectiveness of an oral function intervention for older Taiwanese people. Gerodontology. 2019 Dec;36(4):374-381. doi: 10.1111/ger.12412. Epub 2019 Jul 5. PMID: 31274226. 3. Helmi M, AlDosari M, Tavares M. Phentolamine Mesylate May be a Safe and Effective Option to Reduce Discomfort and Time to Recovery After Dental Care With Local Anesthesia. J Evid Based Dent Pract. 2018 Jun;18(2):181-184. doi: 10.1016/j.jebdp.2018.02.005. Epub 2018 Feb 24. PMID: 29747805. |
| Need for further interventions / maintenance / restoration / number of dental visits | 1. Widbiller M, Knüttel H, Meschi N, Durán-Sindreu Terol F. Effectiveness of endodontic tissue engineering in treatment of apical periodontitis: A systematic review. Int Endod J. 2022 Jun 14. doi: 10.1111/iej.13784. Epub ahead of print. PMID: 35699668. 2. Tickle M, Ricketts DJN, Duncan A, O'Malley L, Donaldson PM, Clarkson JE, Black M, Boyers D, Donaldson M, Floate R, Forrest MM, Fraser A, Glenny AM, Goulao B, McDonald A, Ramsay CR, Ross C, Walsh T, Worthington HV, Young L, Bonetti DL, Gouick J, Mitchell FE, Macpherson LE, Lin YL, Pretty IA, Birch S. Protocol for a Randomised controlled trial to Evaluate the effectiveness and cost benefit of prescribing high dose FLuoride toothpaste in preventing and treating dEntal Caries in high-risk older adulTs (reflect trial). BMC Oral Health. 2019 May 24;19(1):88. doi: 10.1186/s12903-019-0749-x. PMID: 31126270; PMCID: PMC6534863. 3. Stafford GL. The effectiveness of different attachment systems maxillary and mandibular implant overdentures. Evid Based Dent. 2019 Mar;20(1):26-27. doi: 10.1038/s41432-019-0011-z. PMID: 30903126. 4. Effenberger S, Greenwall L, Cebula M, Myburgh N, Simpson K, Smit D, Wicht MJ, Schwendicke F. Cost-effectiveness and efficacy of fluoride varnish for caries prevention in South African children: A cluster-randomized controlled community trial. Community Dent Oral Epidemiol. 2022 Oct;50(5):453-460. doi: 10.1111/cdoe.12702. Epub 2021 Oct 21. PMID: 34676577. 5. Duncan HF, Nagendrababu V, El-Karim I, Dummer PMH. Outcome measures to assess the effectiveness of endodontic treatment for pulpitis and apical periodontitis for use in the development of European Society of Endodontology S3-level clinical practice guidelines: A consensus-based development. Int Endod J. 2021 Dec;54(12):2184-2194. doi: 10.1111/iej.13627. Epub 2021 Sep 22. PMID: 34553383. 6. de Resende GP, Nogueira TE, Leles CR. Effectiveness of a simplified method for final impression in complete denture treatment: A pragmatic clinical trial. Gerodontology. 2019 Dec;36(4):365-373. doi: 10.1111/ger.12411. Epub 2019 Jul 5. PMID: 31276220. 7. Anopa Y, Conway DI. Exploring the cost-effectiveness of child dental caries prevention programmes. Are we comparing apples and oranges? Evid Based Dent. 2020 Mar;21(1):5-7. doi: 10.1038/s41432-020-0085-7. PMID: 32221482. |
| Adverse events | 1. Widbiller M, Knüttel H, Meschi N, Durán-Sindreu Terol F. Effectiveness of endodontic tissue engineering in treatment of apical periodontitis: A systematic review. Int Endod J. 2022 Jun 14. doi: 10.1111/iej.13784. Epub ahead of print. PMID: 35699668. |
| Not failing / success / failure rate | 1. Waterhouse P. Are different pulp treatment techniques and associated medicaments effective for the treatment of extensive decay in primary teeth? Evid Based Dent. 2021 Jan;22(1):12-13. doi: 10.1038/s41432-021-0162-6. PMID: 33772121. 2. Stafford GL. The effectiveness of different attachment systems maxillary and mandibular implant overdentures. Evid Based Dent. 2019 Mar;20(1):26-27. doi: 10.1038/s41432-019-0011-z. PMID: 30903126. 3. Meschi N, Palma PJ, Cabanillas-Balsera D. Effectiveness of revitalization in treating apical periodontitis: A systematic review and meta-analysis. Int Endod J. 2022 May 17. doi: 10.1111/iej.13778. Epub ahead of print. PMID: 35579093. 4. Liu S, Silikas N, Ei-Angbawi A. Analysis of the effectiveness of the fiber-reinforced composite lingual retainer: A systematic review and meta-analysis. Am J Orthod Dentofacial Orthop. 2022 Aug 26:S0889-5406(22)00432-2. doi: 10.1016/j.ajodo.2022.07.003. Epub ahead of print. PMID: 36031511. |
| Cost-effectiveness (perspective costs, willingness to pay, net benefit, net benefits (mean WTP minus mean costs), societal cost) | 1. Tickle M, Ricketts DJN, Duncan A, O'Malley L, Donaldson PM, Clarkson JE, Black M, Boyers D, Donaldson M, Floate R, Forrest MM, Fraser A, Glenny AM, Goulao B, McDonald A, Ramsay CR, Ross C, Walsh T, Worthington HV, Young L, Bonetti DL, Gouick J, Mitchell FE, Macpherson LE, Lin YL, Pretty IA, Birch S. Protocol for a Randomised controlled trial to Evaluate the effectiveness and cost benefit of prescribing high dose FLuoride toothpaste in preventing and treating dEntal Caries in high-risk older adulTs (reflect trial). BMC Oral Health. 2019 May 24;19(1):88. doi: 10.1186/s12903-019-0749-x. PMID: 31126270; PMCID: PMC6534863. 2. Ramsay CR, Clarkson JE, Duncan A, Lamont TJ, Heasman PA, Boyers D, Goulão B, Bonetti D, Bruce R, Gouick J, Heasman L, Lovelock-Hempleman LA, Macpherson LE, McCracken GI, McDonald AM, McLaren-Neil F, Mitchell FE, Norrie JD, van der Pol M, Sim K, Steele JG, Sharp A, Watt G, Worthington HV, Young L. Improving the Quality of Dentistry (IQuaD): a cluster factorial randomised controlled trial comparing the effectiveness and cost-benefit of oral hygiene advice and/or periodontal instrumentation with routine care for the prevention and management of periodontal disease in dentate adults attending dental primary care. Health Technol Assess. 2018 Jul;22(38):1-144. doi: 10.3310/hta22380. PMID: 29984691; PMCID: PMC6055082. 3. Fueki K, Inamochi Y, Yoshida-Kohno E, Wakabayashi N. Cost-effectiveness analysis of prosthetic treatment with thermoplastic resin removable partial dentures. J Prosthodont Res. 2021 Feb 24;65(1):52-55. doi: 10.2186/jpr.JPOR_2019_418. Epub 2020 Sep 9. PMID: 32938866. 4. Victory E, Rhiannon ET, Girvan B, Pauline A, Cynthia PM. Cost-effectiveness Analysis of the Dental RECUR Pragmatic Randomized Controlled Trial: Evaluating a Goal-oriented Talking Intervention to Prevent Reoccurrence of Dental Caries in Children. Appl Health Econ Health Policy. 2022 May;20(3):431-445. doi: 10.1007/s40258-022-00720-5. Epub 2022 Mar 17. PMID: 35298778; PMCID: PMC9021113. 5. Ganzer N, Feldmann I, Petrén S, Bondemark L. A cost-effectiveness analysis of anchorage reinforcement with miniscrews and molar blocks in adolescents: a randomized controlled trial. Eur J Orthod. 2019 Mar 29;41(2):180-187. doi: 10.1093/ejo/cjy041. PMID: 30668660. |
| Caries arrest | 1. Thakur S, Sojan M, Singhal P, Chauhan D. A Comparative Study to Evaluate the Effectiveness of Silver Diamine Fluoride at Different Time Durations of Application in Treating Carious Primary Teeth: A Randomized Trial. Int J Clin Pediatr Dent. 2022;15(Suppl 2):S147-S150. doi: 10.5005/jp-journals-10005-2326. PMID: 35645534; PMCID: PMC9108840. 2. Sihra R, Schroth RJ, Bertone M, Martin H, Patterson B, Mittermuller BA, Lee V, Patterson B, Moffatt ME, Klus B, Fontana M, Robertson L. The Effectiveness of Silver Diamine Fluoride and Fluoride Varnish in Arresting Caries in Young Children and Associated Oral Health-Related Quality of Life. J Can Dent Assoc. 2020 Jun;86:k9. PMID: 32543369. |
| Patient satisfaction / preference / | 1. Stafford GL. The effectiveness of different attachment systems maxillary and mandibular implant overdentures. Evid Based Dent. 2019 Mar;20(1):26-27. doi: 10.1038/s41432-019-0011-z. PMID: 30903126. 2. Nogueira TE, Aguiar FMO, Esfandiari S, Leles CR. Effectiveness of immediately loaded single-implant mandibular overdentures versus mandibular complete dentures: A 1-year follow-up of a randomized clinical trial. J Dent. 2018 Oct;77:43-50. doi: 10.1016/j.jdent.2018.07.006. Epub 2018 Jul 10. PMID: 30006117. 3. Kothari S, Jum'ah AA, Gray AR, M Lyons K, Yap M, Brunton PA. A randomized clinical trial investigating three vital tooth bleaching protocols and associated efficacy, effectiveness and participants' satisfaction. J Dent. 2020 Apr;95:103322. doi: 10.1016/j.jdent.2020.103322. Epub 2020 Mar 19. PMID: 32200009. 4. Fueki K, Inamochi Y, Yoshida-Kohno E, Wakabayashi N. Cost-effectiveness analysis of prosthetic treatment with thermoplastic resin removable partial dentures. J Prosthodont Res. 2021 Feb 24;65(1):52-55. doi: 10.2186/jpr.JPOR_2019_418. Epub 2020 Sep 9. PMID: 32938866. 5. Xia Y, Ma C, Chen J, Witter DJ, Zhang Q, Creugers NHJ. Mandibular residual ridge morphology in relation to complete dentures and implant overdentures-Part II: Predictors for effectiveness of implant overdenture therapy. Clin Implant Dent Relat Res. 2021 Feb;23(1):140-148. doi: 10.1111/cid.12975. Epub 2021 Feb 16. PMID: 33592681. 6. de Resende GP, Nogueira TE, Leles CR. Effectiveness of a simplified method for final impression in complete denture treatment: A pragmatic clinical trial. Gerodontology. 2019 Dec;36(4):365-373. doi: 10.1111/ger.12411. Epub 2019 Jul 5. PMID: 31276220. 7. Baba Y, Sato Y, Owada G, Minakuchi S. Effectiveness of a combination denture-cleaning method versus a mechanical method: comparison of denture cleanliness, patient satisfaction, and oral health-related quality of life. J Prosthodont Res. 2018 Jul;62(3):353-358. doi: 10.1016/j.jpor.2018.01.005. Epub 2018 Feb 7. PMID: 29428169. |
| Tooth erosion | 1. Sivertsen TB, Åstrøm AN, Greve G, Aßmus J, Skeie MS. Effectiveness of an oral health intervention program for children with congenital heart defects. BMC Oral Health. 2018 Mar 23;18(1):50. doi: 10.1186/s12903-018-0495-5. PMID: 29566698; PMCID: PMC5865357. |
| frequency and/or amount of free sugars intake / diet | 1. Shakir A, Barngkgei I, Godson J, Joury E. Effectiveness of school-based behavioural interventions to improve children's oral health by reducing sugar intake and promoting oral hygiene: A rapid review of randomised controlled trials. Community Dent Health. 2021 Nov 29;38(4):275-283. doi: 10.1922/CDH_00014Shakir09. PMID: 34351713. 2. Satyarup D, Dalai RP, Nagarajappa R, Naik D, Mohanty I. Effectiveness of trained health workers in improving the oral hygiene of preschool children. Rocz Panstw Zakl Hig. 2021;72(1):77-82. doi: 10.32394/rpzh.2021.0148. PMID: 33883064. 3. McGowan LJ, Joyes EC, Adams EA, Coyte A, Gavin R, Richmond C, Shabaninejad H, Beyer F, Broadbridge A, Dobson K, Landes D, Moffatt S, Watt RG, Sniehotta FF, Freeman R, Paisi M, Bambra C, Craig D, Kaner E, Ramsay SE. Investigating the Effectiveness and Acceptability of Oral Health and Related Health Behaviour Interventions in Adults with Severe and Multiple Disadvantage: Protocol for a Mixed-Methods Systematic Review. Int J Environ Res Public Health. 2021 Nov 3;18(21):11554. doi: 10.3390/ijerph182111554. PMID: 34770066; PMCID: PMC8582803. 4. Aguirre PEA, Lotto M, Strieder AP, Cruvinel AFP, Cruvinel T. The Effectiveness of Educational Mobile Messages for Assisting in the Prevention of Early Childhood Caries: Protocol for a Randomized Controlled Trial. JMIR Res Protoc. 2019 Sep 3;8(9):e13656. doi: 10.2196/13656. PMID: 31482856; PMCID: PMC6751091. |
| Utilization / attendance | 1. Sanjeevan V, Janakiram C, Joseph J. Effectiveness of school-based dental screening in increasing dental care utilization: A systematic review and meta-analysis. Indian J Dent Res. 2019 Jan-Feb;30(1):117-124. doi: 10.4103/ijdr.IJDR_523_18. PMID: 30900669. 2. Hodgins F, Sherriff A, Gnich W, Ross AJ, Macpherson LMD. The effectiveness of Dental Health Support Workers at linking families with primary care dental practices: a population-wide data linkage cohort study. BMC Oral Health. 2018 Nov 21;18(1):191. doi: 10.1186/s12903-018-0650-z. PMID: 30463549; PMCID: PMC6249895. |
| alcohol consumption / substance use | 1. Plessas A, Nasser M. Can we deliver effective alcohol-related brief advice in general dental practice? Evid Based Dent. 2019 Sep;20(3):77-78. doi: 10.1038/s41432-019-0036-3. PMID: 31562406. 2. McGowan LJ, Joyes EC, Adams EA, Coyte A, Gavin R, Richmond C, Shabaninejad H, Beyer F, Broadbridge A, Dobson K, Landes D, Moffatt S, Watt RG, Sniehotta FF, Freeman R, Paisi M, Bambra C, Craig D, Kaner E, Ramsay SE. Investigating the Effectiveness and Acceptability of Oral Health and Related Health Behaviour Interventions in Adults with Severe and Multiple Disadvantage: Protocol for a Mixed-Methods Systematic Review. Int J Environ Res Public Health. 2021 Nov 3;18(21):11554. doi: 10.3390/ijerph182111554. PMID: 34770066; PMCID: PMC8582803. |
| Mucositis (presence, severity, duration) | 1. Oshvandi K, Vafaei SY, Kamallan SR, Khazaei S, Ranjbar H, Mohammadi F. Effectiveness of zinc chloride mouthwashes on oral mucositis and weight of patients with cancer undergoing chemotherapy. BMC Oral Health. 2021 Jul 22;21(1):364. doi: 10.1186/s12903-021-01706-w. PMID: 34294072; PMCID: PMC8296564. 2. Mohammadi F, Oshvandi K, Kamallan SR, Khazaei S, Ranjbar H, Ahmadi-Motamayel F, Gillespie M, Jenabi E, Vafaei SY. Effectiveness of sodium bicarbonate and zinc chloride mouthwashes in the treatment of oral mucositis and quality of life in patients with cancer under chemotherapy. Nurs Open. 2022 May;9(3):1602-1611. doi: 10.1002/nop2.1168. Epub 2022 Feb 16. PMID: 35170247; PMCID: PMC8994952. 3. Martins AFL, Nogueira TE, Morais MO, Oton-Leite AF, Valadares MC, Batista AC, Freitas NMA, Leles CR, Mendonça EF. Effect of photobiomodulation on the severity of oral mucositis and molecular changes in head and neck cancer patients undergoing radiotherapy: a study protocol for a cost-effectiveness randomized clinical trial. Trials. 2019 Feb 1;20(1):97. doi: 10.1186/s13063-019-3196-8. PMID: 30709370; PMCID: PMC6359861. 4. Lopes Martins AF, Nogueira TE, Morais MO, de Sousa-Neto SS, Oton-Leite AF, Valadares MC, Aires Freitas NM, Leles CR, Mendonça EF. Cost-effectiveness randomized clinical trial on the effect of photobiomodulation therapy for prevention of radiotherapy-induced severe oral mucositis in a Brazilian cancer hospital setting. Support Care Cancer. 2021 Mar;29(3):1245-1256. doi: 10.1007/s00520-020-05607-6. Epub 2020 Jul 3. PMID: 32621262. 5. Dao VA, Bilstein A, Overhagen S, Géczi L, Baráth Z, Mösges R. Effectiveness, Tolerability, and Safety of Ectoine-Containing Mouthwash Versus Those of a Calcium Phosphate Mouthwash for the Treatment of Chemotherapy-Induced Oral Mucositis: A Prospective, Active-Controlled, Non-interventional Study. Oncol Ther. 2018 Jun;6(1):59-72. doi: 10.1007/s40487-018-0060-z. Epub 2018 May 9. PMID: 32700140; PMCID: PMC7360009. 6. Bezerra PMM, Sampaio MEA, Dos Santos FG, Ribeiro ILA, Santiago BM, de Sousa SA, Valença AMG. The effectiveness of an oral health education and prevention program on the incidence and severity of oral mucositis in pediatric cancer patients: a non-randomized controlled study. Support Care Cancer. 2021 Dec;29(12):7877-7885. doi: 10.1007/s00520-021-06387-3. Epub 2021 Jun 29. PMID: 34189606. |
| Weight gain (mucositis, chemotherapy) | 1. Oshvandi K, Vafaei SY, Kamallan SR, Khazaei S, Ranjbar H, Mohammadi F. Effectiveness of zinc chloride mouthwashes on oral mucositis and weight of patients with cancer undergoing chemotherapy. BMC Oral Health. 2021 Jul 22;21(1):364. doi: 10.1186/s12903-021-01706-w. PMID: 34294072; PMCID: PMC8296564. |
| Orthodontic (e.g. prevention of mand. Incisor relapse, malocclusion, tooth movement (time)) | 1. Nagani NI, Ahmed I. Effectiveness of Two Types of Fixed Lingual Retainers in Preventing Mandibular Incisor Relapse. J Coll Physicians Surg Pak. 2020 Mar;30(3):282-286. doi: 10.29271/jcpsp.2020.03.282. PMID: 32169137. 2. Liu S, Silikas N, Ei-Angbawi A. Analysis of the effectiveness of the fiber-reinforced composite lingual retainer: A systematic review and meta-analysis. Am J Orthod Dentofacial Orthop. 2022 Aug 26:S0889-5406(22)00432-2. doi: 10.1016/j.ajodo.2022.07.003. Epub ahead of print. PMID: 36031511. 3. Graf I, Bock NC, Bartzela T, Röper V, Schumann U, Reck K, Christ H, Höfer K, Fritz U, Wiechmann D, Jost-Brinkmann PG, Wolf M, Ruf S, Braumann B. Quality of orthodontic care-A multicenter cohort study in Germany : Part 1: Evaluation of effectiveness of orthodontic treatments and predictive factors. J Orofac Orthop. 2022 Sep;83(5):291-306. doi: 10.1007/s00056-021-00304-3. Epub 2021 Jun 17. PMID: 34142175; PMCID: PMC9395451. 4. Fattori L, Sendyk M, de Paiva JB, Normando D, Neto JR. Micro-osteoperforation effectiveness on tooth movement rate and impact on oral health related quality of life. Angle Orthod. 2020 Sep 1;90(5):640-647. doi: 10.2319/110819-707.1. PMID: 33378487; PMCID: PMC8032262. |
| taste | 1. Michelson M, Veitz-Keenan A. Taste disturbances - are there any effective treatments? Evid Based Dent. 2018 Jun;19(2):60-61. doi: 10.1038/sj.ebd.6401312. PMID: 29930370. |
| Tooth loss / risk for tooth loss / need for extraction | 1. McLister C, Donnelly M, Cardwell C, Moore C, McKenna G. Effectiveness of prosthodontic interventions and survival of remaining teeth in adult patients with shortened dental arches-Protocol for a systematic review and meta-analysis. BDJ Open. 2018 Apr 27;4:17023. doi: 10.1038/bdjopen.2017.23. PMID: 30637120; PMCID: PMC5921535. 2. McLister C, Donnelly M, Cardwell CR, Moore C, O'Neill C, Brocklehurst P, McKenna G. Effectiveness of prosthodontic interventions and survival of remaining teeth in adult patients with shortened dental arches-A systematic review. J Dent. 2018 Nov;78:31-39. doi: 10.1016/j.jdent.2018.02.003. Epub 2018 Feb 21. PMID: 29476794. 3. Effenberger S, Greenwall L, Cebula M, Myburgh N, Simpson K, Smit D, Wicht MJ, Schwendicke F. Cost-effectiveness and efficacy of fluoride varnish for caries prevention in South African children: A cluster-randomized controlled community trial. Community Dent Oral Epidemiol. 2022 Oct;50(5):453-460. doi: 10.1111/cdoe.12702. Epub 2021 Oct 21. PMID: 34676577. |
| Halitosis, bad breath, etc. | 1. Matsumura Y, Hinode D, Fukui M, Yoshioka M, Asakuma H, Takii H. Effectiveness of an oral care tablet containing kiwifruit powder in reducing oral bacteria in tongue coating: A crossover trial. Clin Exp Dent Res. 2020 Apr;6(2):197-206. doi: 10.1002/cre2.262. Epub 2019 Dec 3. PMID: 32250572; PMCID: PMC7133734. 2. Khurana C, Tandon S, Chinmaya BR. A crossover clinical trial to assess the effectiveness of different oral hygiene regimens on the reduction of morning bad breath in healthy young adults. Indian J Dent Res. 2018 Jul-Aug;29(4):434-439. doi: 10.4103/ijdr.IJDR_800_16. PMID: 30127192. |
| Bone healing | 1. Lopes CCA, Limirio JPJO, Zanatta LSA, Simamoto VRN, Dechichi P, Limirio APHJO. Effectiveness of Photobiomodulation Therapy on Human Bone Healing in Dentistry: A Systematic Review. Photobiomodul Photomed Laser Surg. 2022 Jul;40(7):440-453. doi: 10.1089/photob.2021.0092. Epub 2022 May 6. PMID: 35527692. |
| Oral health (in general)  Oral Assessment Guide (voice, swallowing, lips, tongue, saliva, mucous membranes, gingiva and teeth or dentures), Oral Health Assessment Tool (OHAT), oral health status (OHS) | 1. Liao YC, Hsu LF, Hsieh LY, Luo YY. Effectiveness of green tea mouthwash for improving oral health status in oral cancer patients: A single-blind randomized controlled trial. Int J Nurs Stud. 2021 Sep;121:103985. doi: 10.1016/j.ijnurstu.2021.103985. Epub 2021 May 25. PMID: 34186380. 2. Czwikla J, Herzberg A, Kapp S, Kloep S, Rothgang H, Nitschke I, Haffner C, Hoffmann F. Effectiveness of a Dental Intervention to Improve Oral Health among Home Care Recipients: A Randomized Controlled Trial. Int J Environ Res Public Health. 2021 Sep 3;18(17):9339. doi: 10.3390/ijerph18179339. PMID: 34501925; PMCID: PMC8430536. 3. Bramantoro T, Santoso CMA, Hariyani N, Setyowati D, Zulfiana AA, Nor NAM, Nagy A, Pratamawari DNP, Irmalia WR. Effectiveness of the school-based oral health promotion programmes from preschool to high school: A systematic review. PLoS One. 2021 Aug 11;16(8):e0256007. doi: 10.1371/journal.pone.0256007. PMID: 34379685; PMCID: PMC8357156. |
| Xerostomia | 1. Kvalheim SF, Marthinussen MC, Haugen DF, Berg E, Strand GV, Lie SA. Randomized controlled trial of the effectiveness of three different oral moisturizers in palliative care patients. Eur J Oral Sci. 2019 Dec;127(6):523-530. doi: 10.1111/eos.12655. Epub 2019 Jul 20. PMID: 31325345. |
| color of teeth, appearance, psychosocial impact of estethics | 1. Kothari S, Jum'ah AA, Gray AR, M Lyons K, Yap M, Brunton PA. A randomized clinical trial investigating three vital tooth bleaching protocols and associated efficacy, effectiveness and participants' satisfaction. J Dent. 2020 Apr;95:103322. doi: 10.1016/j.jdent.2020.103322. Epub 2020 Mar 19. PMID: 32200009. 2. Fueki K, Inamochi Y, Yoshida-Kohno E, Wakabayashi N. Cost-effectiveness analysis of prosthetic treatment with thermoplastic resin removable partial dentures. J Prosthodont Res. 2021 Feb 24;65(1):52-55. doi: 10.2186/jpr.JPOR_2019_418. Epub 2020 Sep 9. PMID: 32938866. 3. Ferraz NKL, Nogueira LC, Neiva IM, Ferreira RC, Moreira AN, Magalhães CS. Longevity, effectiveness, safety, and impact on quality of life of low-concentration hydrogen peroxides in-office bleaching: a randomized clinical trial. Clin Oral Investig. 2019 May;23(5):2061-2070. doi: 10.1007/s00784-018-2607-7. Epub 2018 Sep 20. PMID: 30238417. 4. Bersezio C, Ledezma P, Mayer C, Rivera O, Junior OBO, Fernández E. Effectiveness and effect of non-vital bleaching on the quality of life of patients up to 6 months post-treatment: a randomized clinical trial. Clin Oral Investig. 2018 Dec;22(9):3013-3019. doi: 10.1007/s00784-018-2389-y. Epub 2018 Feb 17. PMID: 29455372. 5. Water fluoridation safe and effective. Br Dent J. 2018 May 11;224(9):674. doi: 10.1038/sj.bdj.2018.377. PMID: 29747180. |
| Periodontal health (e.g. attachment loss, BoP, probing pocket depth) | 1. Keyong E, Thitasomakul S, Tianviwat S. Effectiveness of an Oral Health Promotion Program for the Elderly in Khiri Mat District, Sukhothai Province: A Randomized Control Trial. J Int Soc Prev Community Dent. 2019 May-Jun;9(3):225-231. doi: 10.4103/jispcd.JISPCD_27_19. Epub 2019 Jun 7. PMID: 31198693; PMCID: PMC6559037. 2. Clarkson JE, Pitts NB, Fee PA, Goulao B, Boyers D, Ramsay CR, Floate R, Braid HJ, Ord FS, Worthington HV, van der Pol M, Young L, Freeman R, Gouick J, Humphris GM, Mitchell FE, McDonald AM, Norrie JDT, Sim K, Douglas G, Ricketts D. Examining the effectiveness of different dental recall strategies on maintenance of optimum oral health: the INTERVAL dental recalls randomised controlled trial. Br Dent J. 2021 Feb;230(4):236-243. doi: 10.1038/s41415-021-2612-0. Epub 2021 Feb 26. PMID: 33637927; PMCID: PMC7908962. 3. Clarkson JE, Pitts NB, Bonetti D, Boyers D, Braid H, Elford R, Fee PA, Floate R, Goulão B, Humphris G, Needleman I, Norrie JDT, Ord F, van der Pol M, Ramsay CR, Ricketts DNJ, Worthington HV, Young L; INTERVAL Trial Collaboration. INTERVAL (investigation of NICE technologies for enabling risk-variable-adjusted-length) dental recalls trial: a multicentre randomised controlled trial investigating the best dental recall interval for optimum, cost-effective maintenance of oral health in dentate adults attending dental primary care. BMC Oral Health. 2018 Aug 7;18(1):135. doi: 10.1186/s12903-018-0587-2. PMID: 30086747; PMCID: PMC6081817. 4. Chatzopoulos GS, Karakostas P, Kavakloglou S, Assimopoulou A, Barmpalexis P, Tsalikis L. Clinical Effectiveness of Herbal Oral Care Products in Periodontitis Patients: A Systematic Review. Int J Environ Res Public Health. 2022 Aug 15;19(16):10061. doi: 10.3390/ijerph191610061. PMID: 36011693; PMCID: PMC9408146. 5. Aguirre PEA, Lotto M, Strieder AP, Cruvinel AFP, Cruvinel T. The Effectiveness of Educational Mobile Messages for Assisting in the Prevention of Early Childhood Caries: Protocol for a Randomized Controlled Trial. JMIR Res Protoc. 2019 Sep 3;8(9):e13656. doi: 10.2196/13656. PMID: 31482856; PMCID: PMC6751091. |
| General health | 1. Ho BV, Weijenberg RAF, van der Maarel-Wierink CD, Visscher CM, van der Putten GJ, Scherder EJA, Lobbezoo F. Effectiveness of the implementation project 'Don't forget the mouth!' of community dwelling older people with dementia: a prospective longitudinal single-blind multicentre study protocol (DFTM!). BMC Oral Health. 2019 May 28;19(1):91. doi: 10.1186/s12903-019-0786-5. PMID: 31138273; PMCID: PMC6537198. |
| Care dependency | 1. Ho BV, Weijenberg RAF, van der Maarel-Wierink CD, Visscher CM, van der Putten GJ, Scherder EJA, Lobbezoo F. Effectiveness of the implementation project 'Don't forget the mouth!' of community dwelling older people with dementia: a prospective longitudinal single-blind multicentre study protocol (DFTM!). BMC Oral Health. 2019 May 28;19(1):91. doi: 10.1186/s12903-019-0786-5. PMID: 31138273; PMCID: PMC6537198. |
| compliance of the health care professionals | 1. Ho BV, Weijenberg RAF, van der Maarel-Wierink CD, Visscher CM, van der Putten GJ, Scherder EJA, Lobbezoo F. Effectiveness of the implementation project 'Don't forget the mouth!' of community dwelling older people with dementia: a prospective longitudinal single-blind multicentre study protocol (DFTM!). BMC Oral Health. 2019 May 28;19(1):91. doi: 10.1186/s12903-019-0786-5. PMID: 31138273; PMCID: PMC6537198. |
| (Dental) referral | 1. Gurgel-Juarez N, Torres-Pereira C, Haddad AE, Sheehy L, Finestone H, Mallet K, Wiseman M, Hour K, Flowers HL. Accuracy and effectiveness of teledentistry: a systematic review of systematic reviews. Evid Based Dent. 2022 Jul 8:1–8. doi: 10.1038/s41432-022-0257-8. Epub ahead of print. PMID: 35804195; PMCID: PMC9264296. 2. Alayadi H, Bernabé E, Sabbah W. Effectiveness of School Dental Screening to Reduce Untreated Caries: A Cluster-Randomized Controlled Trial. Caries Res. 2021;55(6):577-584. doi: 10.1159/000519770. Epub 2021 Sep 22. PMID: 34551412. 3. Alayadi H, Sabbah W, Bernabé E. Effectiveness of school dental screening on dental visits and untreated caries among primary schoolchildren: study protocol for a cluster randomised controlled trial. Trials. 2018 Apr 13;19(1):224. doi: 10.1186/s13063-018-2619-2. PMID: 29653545; PMCID: PMC5899334. |
| Treatment planning | 1. Gurgel-Juarez N, Torres-Pereira C, Haddad AE, Sheehy L, Finestone H, Mallet K, Wiseman M, Hour K, Flowers HL. Accuracy and effectiveness of teledentistry: a systematic review of systematic reviews. Evid Based Dent. 2022 Jul 8:1–8. doi: 10.1038/s41432-022-0257-8. Epub ahead of print. PMID: 35804195; PMCID: PMC9264296. |
| Jaw Functional Limitation Scale 20 (JFLS-20) | 1. Ekici Ö, Dündar Ü, Büyükbosna M. Effectiveness of high-intensity laser therapy in patients with myogenic temporomandibular joint disorder: A double-blind, placebo-controlled study. J Stomatol Oral Maxillofac Surg. 2022 Jun;123(3):e90-e96. doi: 10.1016/j.jormas.2021.06.009. Epub 2021 Jun 24. PMID: 34174507. |
| Quality of dentures, cleaning dentures | 1. de Resende GP, Nogueira TE, Leles CR. Effectiveness of a simplified method for final impression in complete denture treatment: A pragmatic clinical trial. Gerodontology. 2019 Dec;36(4):365-373. doi: 10.1111/ger.12411. Epub 2019 Jul 5. PMID: 31276220. 2. Baba Y, Sato Y, Owada G, Minakuchi S. Effectiveness of a combination denture-cleaning method versus a mechanical method: comparison of denture cleanliness, patient satisfaction, and oral health-related quality of life. J Prosthodont Res. 2018 Jul;62(3):353-358. doi: 10.1016/j.jpor.2018.01.005. Epub 2018 Feb 7. PMID: 29428169. |
| Sleep (quality) | 1. de Resende CMBM, de Oliveira Medeiros FGL, de Figueiredo Rêgo CR, Bispo ASL, Barbosa GAS, de Almeida EO. Short-term effectiveness of conservative therapies in pain, quality of life, and sleep in patients with temporomandibular disorders: A randomized clinical trial. Cranio. 2021 Jul;39(4):335-343. doi: 10.1080/08869634.2019.1627068. Epub 2019 Jun 15. PMID: 31204605. |
| Patient cooperation / compliance | 1. Balian A, Cirio S, Salerno C, Wolf TG, Campus G, Cagetti MG. Is Visual Pedagogy Effective in Improving Cooperation Towards Oral Hygiene and Dental Care in Children with Autism Spectrum Disorder? A Systematic Review and Meta-Analysis. Int J Environ Res Public Health. 2021 Jan 18;18(2):789. doi: 10.3390/ijerph18020789. PMID: 33477719; PMCID: PMC7832292. 2. AlBhaisi IN, Kumar MSTS, Engapuram A, Shafiei Z, Zakaria ASI, Mohd-Said S, McGrath C. Effectiveness of psychological techniques in dental management for children with autism spectrum disorder: a systematic literature review. BMC Oral Health. 2022 May 6;22(1):162. doi: 10.1186/s12903-022-02200-7. PMID: 35524299; PMCID: PMC9074276. |
| Diagnosis (sensitivity, specificity), | 1. AlShaya MS, Assery MK, Pani SC. Reliability of mobile phone teledentistry in dental diagnosis and treatment planning in mixed dentition. J Telemed Telecare. 2020 Jan-Feb;26(1-2):45-52. doi: 10.1177/1357633X18793767. Epub 2018 Aug 22. PMID: 30134778. |
| Screening | 1. Alayadi H, Bernabé E, Sabbah W. Effectiveness of School Dental Screening to Reduce Untreated Caries: A Cluster-Randomized Controlled Trial. Caries Res. 2021;55(6):577-584. doi: 10.1159/000519770. Epub 2021 Sep 22. PMID: 34551412. 2. Alayadi H, Sabbah W, Bernabé E. Effectiveness of school dental screening on dental visits and untreated caries among primary schoolchildren: study protocol for a cluster randomised controlled trial. Trials. 2018 Apr 13;19(1):224. doi: 10.1186/s13063-018-2619-2. PMID: 29653545; PMCID: PMC5899334. |
| Clinical skill of provider | 1. Al-Ansari A, Nazir MA. Dentists' responses about the effectiveness of continuing education activities. Eur J Dent Educ. 2018 Nov;22(4):e737-e744. doi: 10.1111/eje.12388. Epub 2018 Aug 11. PMID: 30098098. |
| Electronic health literacy | 1. Aguirre PEA, Lotto M, Strieder AP, Cruvinel AFP, Cruvinel T. The Effectiveness of Educational Mobile Messages for Assisting in the Prevention of Early Childhood Caries: Protocol for a Randomized Controlled Trial. JMIR Res Protoc. 2019 Sep 3;8(9):e13656. doi: 10.2196/13656. PMID: 31482856; PMCID: PMC6751091. |
| Activity interference | 1. Aggarwal VR, Fu Y, Main CJ, Wu J. The effectiveness of self-management interventions in adults with chronic orofacial pain: A systematic review, meta-analysis and meta-regression. Eur J Pain. 2019 May;23(5):849-865. doi: 10.1002/ejp.1358. Epub 2019 Feb 7. PMID: 30620145. |

# Patient-centeredness

According to “An International Working Definition for Quality of Oral Healthcare” (Righolt et. al, 2019) aims are:

In order to be considered patient-centered, oral healthcare should

• Be respectful of and responsive to individual patient preferences, needs, values, fears, concerns, and/or cultures.

• Follow a shared decision-making model when making clinical decisions. To gain a patient’s trust, the oral healthcare

professional should communicate with and listen to the patient, then informing, educating, and guiding the patient to

ensure that patient values shape all clinical decisions.

Number of titles screened: 103

Number of abstracts screened: 82

Number of full texts screened: 11

| **Possible topics deduced from literature** | **Reference** |
| --- | --- |
| Feedback from patients | Previously covered (old literature screening see Righolt et. al, 2019 |
| Oral health literacy | Previously covered (old literature screening see Righolt et. al, 2019 |
| Routine feedback | Previously covered (old literature screening see Righolt et. al, 2019 |
| Expectations and views of patients | Previously covered (old literature screening see Righolt et. al, 2019 |
| Communication with patients | Previously covered (old literature screening see Righolt et. al, 2019 |
| Ambiance | Previously covered (old literature screening see Righolt et. al, 2019 |
| Patient involvement decision making | Previously covered (old literature screening see Righolt et. al, 2019 |
| Patient satisfaction | Previously covered (old literature screening see Righolt et. al, 2019 |
| Interprofessional care | Previously covered (old literature screening see Righolt et. al, 2019 |
| Motivational interviewing | Previously covered (old literature screening see Righolt et. al, 2019 |
| Respectfulness | Previously covered (old literature screening see Righolt et. al, 2019 |
| Involvement in treatment planning/shared decision making | Previously covered (old literature screening see Righolt et. al, 2019 |
| Patient values | Previously covered (old literature screening see Righolt et. al, 2019 |
| Informing the patient | Previously covered (old literature screening see Righolt et. al, 2019 |
| Patient needs/desires | Previously covered (old literature screening see Righolt et. al, 2019 |
| Tailored care | Previously covered (old literature screening see Righolt et. al, 2019 |
| Patient perception of disease and treatment (1. pressure: physical, psychosocial, financial; 2. coping adaptation; 3.reflection & evaluation) | 1. Yin J, Li Y, Feng M, Li L. Understanding the feelings and experiences of patients with periodontal disease: a qualitative meta-synthesis. Health Qual Life Outcomes. 2022 Aug 26;20(1):126. doi: 10.1186/s12955-022-02042-5. PMID: 36028888; PMCID: PMC9419312. |
| Patient feelings, emotional / psychological support, supportive environment | 1. Yin J, Li Y, Feng M, Li L. Understanding the feelings and experiences of patients with periodontal disease: a qualitative meta-synthesis. Health Qual Life Outcomes. 2022 Aug 26;20(1):126. doi: 10.1186/s12955-022-02042-5. PMID: 36028888; PMCID: PMC9419312. 2. Shrivastava R, Couturier Y, Kadoch N, Girard F, Bedos C, Macdonald ME, Torrie J, Emami E. Patients' perspectives on integrated oral healthcare in a northern Quebec Indigenous primary health care organisation: a qualitative study. BMJ Open. 2019 Jul 30;9(7):e030005. doi: 10.1136/bmjopen-2019-030005. PMID: 31366663; PMCID: PMC6677955. |
| Use of PROMs & PREMs (e.g. OHRQoL, satisfaction) also relevant for research to be more useful, the value of outcomes to patients should be checked in order to effectively drive quality improvement | 1. Wong J, Cheung GSP, Lee AHC, McGrath C, Neelakantan P. PROMs Following Root Canal Treatment and Surgical Endodontic Treatment. Int Dent J. 2022 Jul 21:S0020-6539(22)00127-7. doi: 10.1016/j.identj.2022.06.015. Epub ahead of print. PMID: 35871899. 2. Wąsacz K, Hukowska D, Chomyszyn-Gajewska M. Oral Health Impact Profile in adult Polish population. Folia Med Cracov. 2022;62(1):137-149. doi: 10.24425/fmc.2022.141696. PMID: 36088598. 3. Scott JM, Gadbury-Amyot CC, Hoffman AM, Simmer-Beck ML. Associations of Self-Reported Oral Health Quality of Life with Actual Oral Health Status in Children. J Dent Hyg. 2021 Feb;95(1):57-66. PMID: 33627454. 4. Perazzo MF, Serra-Negra JM, Firmino RT, Pordeus IA, Martins-JÚnior PA, Paiva SM. Patient-centered assessments: how can they be used in dental clinical trials? Braz Oral Res. 2020;34 Suppl 2:e075. doi: 10.1590/1807-3107bor-2020.vol34.0075. Epub 2020 Aug 7. PMID: 32785488. 5. Inglehart MR, Albino J, Feine JS, Okunseri C. Sociodemographic Changes and Oral Health Inequities: Dental Workforce Considerations. JDR Clin Trans Res. 2022 Oct;7(1_suppl):5S-15S. doi: 10.1177/23800844221116832. PMID: 36121138. 6. Hillebrecht AL, Hrasky V, Anten C, Wiegand A. Changes in the oral health-related quality of life in adult patients with intellectual disabilities after dental treatment under general anesthesia. Clin Oral Investig. 2019 Oct;23(10):3895-3903. doi: 10.1007/s00784-019-02820-4. Epub 2019 Feb 1. PMID: 30707300. 7. Hua F. Increasing the Value of Orthodontic Research Through the Use of Dental Patient-Reported Outcomes. J Evid Based Dent Pract. 2019 Jun;19(2):99-105. doi: 10.1016/j.jebdp.2019.04.005. Epub 2019 Apr 19. PMID: 31326063. 8. Barber S, Bekker HL, Meads D, Pavitt S, Khambay B. Identification and appraisal of outcome measures used to evaluate hypodontia care: A systematic review. Am J Orthod Dentofacial Orthop. 2018 Feb;153(2):184-194.e18. doi: 10.1016/j.ajodo.2017.10.010. PMID: 29407495. |
| Patient activation | 1. Vamos CA, Merrell L, Livingston TA, Dias E, Detman L, Louis J, Daley E. "I Didn't Know": Pregnant Women's Oral Health Literacy Experiences and Future Intervention Preferences. Womens Health Issues. 2019 Nov-Dec;29(6):522-528. doi: 10.1016/j.whi.2019.05.005. Epub 2019 Jun 21. PMID: 31235347. |
| Dental workforce – patient relationship, trust | 1. Song Y, Luzzi L, Brennan DS. Trust in dentist-patient relationships: mapping the relevant concepts. Eur J Oral Sci. 2020 Apr;128(2):110-119. doi: 10.1111/eos.12686. Epub 2020 Mar 10. PMID: 32154607. 2. Rosing K, Leggett H, Csikar J, Vinall-Collier K, Christensen LB, Whelton H, Douglas GVA. Barriers and facilitators for prevention in Danish dental care. Acta Odontol Scand. 2019 Aug;77(6):439-451. doi: 10.1080/00016357.2019.1587503. Epub 2019 Mar 25. PMID: 30905244. |
| No blame culture | 1. Yin J, Li Y, Feng M, Li L. Understanding the feelings and experiences of patients with periodontal disease: a qualitative meta-synthesis. Health Qual Life Outcomes. 2022 Aug 26;20(1):126. doi: 10.1186/s12955-022-02042-5. PMID: 36028888; PMCID: PMC9419312. 2. Song Y, Luzzi L, Brennan DS. Trust in dentist-patient relationships: mapping the relevant concepts. Eur J Oral Sci. 2020 Apr;128(2):110-119. doi: 10.1111/eos.12686. Epub 2020 Mar 10. PMID: 32154607. |
| Cultural competence | 1. Shrivastava R, Couturier Y, Kadoch N, Girard F, Bedos C, Macdonald ME, Torrie J, Emami E. Patients' perspectives on integrated oral healthcare in a northern Quebec Indigenous primary health care organisation: a qualitative study. BMJ Open. 2019 Jul 30;9(7):e030005. doi: 10.1136/bmjopen-2019-030005. PMID: 31366663; PMCID: PMC6677955. 2. Reynolds JC, Damiano PC, Herndon JB. Patient centered dental home: Building a framework for dental quality measurement and improvement. J Public Health Dent. 2021 Oct 26. doi: 10.1111/jphd.12482. Epub ahead of print. PMID: 34704254. 3. Inglehart MR, Albino J, Feine JS, Okunseri C. Sociodemographic Changes and Oral Health Inequities: Dental Workforce Considerations. JDR Clin Trans Res. 2022 Oct;7(1_suppl):5S-15S. doi: 10.1177/23800844221116832. PMID: 36121138. 4. Dolce MC, Parker JL, Savageau JA, Da Silva JD. Older Adult Patients' Experience of Care in a Dental School Clinic. J Dent Educ. 2019 Sep;83(9):1039-1046. doi: 10.21815/JDE.019.104. Epub 2019 May 27. PMID: 31133617. |
| Raising awareness, involving family, friends, community | 1. Shrivastava R, Couturier Y, Kadoch N, Girard F, Bedos C, Macdonald ME, Torrie J, Emami E. Patients' perspectives on integrated oral healthcare in a northern Quebec Indigenous primary health care organisation: a qualitative study. BMJ Open. 2019 Jul 30;9(7):e030005. doi: 10.1136/bmjopen-2019-030005. PMID: 31366663; PMCID: PMC6677955. |
| Access to personal health data, patient-health-portals | 1. Shimpi N, Schwei K, Cooper S, Chyou PH, Acharya A. Understanding patients' oral health information needs: Findings of a survey on use of patient portals in dentistry. J Am Dent Assoc. 2018 Mar;149(3):184-190. doi: 10.1016/j.adaj.2017.09.051. Epub 2018 Feb 1. Erratum in: J Am Dent Assoc. 2018 Aug;149(8):670. PMID: 29395012. |
| (recognition of) Oral manifestations of disease and disease related impairments (also psychosocial) | 1. Schmalz G, Patschan S, Patschan D, Ziebolz D. Oral-Health-Related Quality of Life in Adult Patients with Rheumatic Diseases-A Systematic Review. J Clin Med. 2020 Apr 19;9(4):1172. doi: 10.3390/jcm9041172. PMID: 32325846; PMCID: PMC7231140. 2. Protudjer JLP, Billedeau C, Hurst K, Schroth R, Stavropoulou C, Kelekis-Cholakis A, Hitchon CA. Oral Health in Rheumatoid Arthritis: Listening to Patients. JDR Clin Trans Res. 2022 Apr;7(2):127-134. doi: 10.1177/23800844211012678. Epub 2021 May 5. PMID: 33949224. |
| Seeing patient as a whole, person-focused care, (also: special needs: chronic disease, mental health, behavioral, physical, social) | 1. Salkinoja P, Tuononen T, Suominen AL, Lammintakanen J, Raittio E. Oral health care quality as viewed by leading dentists and their superiors - a qualitative study. Acta Odontol Scand. 2022 Jan;80(1):38-43. doi: 10.1080/00016357.2021.1936161. Epub 2021 Jun 21. PMID: 34153210. 2. Lee H, Chalmers NI, Brow A, Boynes S, Monopoli M, Doherty M, Croom O, Engineer L. Person-centered care model in dentistry. BMC Oral Health. 2018 Nov 29;18(1):198. doi: 10.1186/s12903-018-0661-9. PMID: 30497465; PMCID: PMC6267887. 3. Inglehart MR, Albino J, Feine JS, Okunseri C. Sociodemographic Changes and Oral Health Inequities: Dental Workforce Considerations. JDR Clin Trans Res. 2022 Oct;7(1_suppl):5S-15S. doi: 10.1177/23800844221116832. PMID: 36121138. |
| Good availability of services (e.g. opening hours), accommodating | 1. Salkinoja P, Tuononen T, Suominen AL, Lammintakanen J, Raittio E. Oral health care quality as viewed by leading dentists and their superiors - a qualitative study. Acta Odontol Scand. 2022 Jan;80(1):38-43. doi: 10.1080/00016357.2021.1936161. Epub 2021 Jun 21. PMID: 34153210. 2. Reynolds JC, Damiano PC, Herndon JB. Patient centered dental home: Building a framework for dental quality measurement and improvement. J Public Health Dent. 2021 Oct 26. doi: 10.1111/jphd.12482. Epub ahead of print. PMID: 34704254. |
| Dental / health registry (e.g. caries risk assessment) | 1. Ruff JC, Herndon JB, Horton RA, Lynch J, Mathwig DC, Leonard A, Aravamudhan K. Developing a caries risk registry to support caries risk assessment and management for children: A quality improvement initiative. J Public Health Dent. 2018 Mar;78(2):134-143. doi: 10.1111/jphd.12253. Epub 2017 Oct 27. PMID: 29077195. 2. Martin J, Mills S, Foley ME. Innovative Models of Dental Care Delivery and Coverage: Patient-Centric Dental Benefits Based on Digital Oral Health Risk Assessment. Dent Clin North Am. 2018 Apr;62(2):319-325. doi: 10.1016/j.cden.2017.11.009. PMID: 29478460. |
| Community connection (e.g. connects patients with needed community & social services) | 1. Reynolds JC, Damiano PC, Herndon JB. Patient centered dental home: Building a framework for dental quality measurement and improvement. J Public Health Dent. 2021 Oct 26. doi: 10.1111/jphd.12482. Epub ahead of print. PMID: 34704254. |
| Physical & socioeconomic assessment | 1. León S, Giacaman RA. Proposal for a Conceptual Framework for the Development of Geriatric Dentistry. J Dent Res. 2022 Mar;101(3):247-252. doi: 10.1177/00220345211042268. Epub 2021 Sep 28. PMID: 34581227. 2. Lee H, Chalmers NI, Brow A, Boynes S, Monopoli M, Doherty M, Croom O, Engineer L. Person-centered care model in dentistry. BMC Oral Health. 2018 Nov 29;18(1):198. doi: 10.1186/s12903-018-0661-9. PMID: 30497465; PMCID: PMC6267887. 3. Fernando S, Tadakamadla SK, Bakr M, Scuffham PA, Johnson NW. Indicators of Risk for Dental Caries in Children: A Holistic Approach. JDR Clin Trans Res. 2019 Oct;4(4):333-341. doi: 10.1177/2380084419834236. Epub 2019 Apr 30. PMID: 31039050. |
| Person-/ patient-centered health system design (supported by values, payment environments, encouraging personal health ownership, respects patients’ dignity, adaptable to input from person/patient & provider) | 1. Lee H, Chalmers NI, Brow A, Boynes S, Monopoli M, Doherty M, Croom O, Engineer L. Person-centered care model in dentistry. BMC Oral Health. 2018 Nov 29;18(1):198. doi: 10.1186/s12903-018-0661-9. PMID: 30497465; PMCID: PMC6267887. |
| Interoperability of general & dental electronic health records | 1. Lee H, Chalmers NI, Brow A, Boynes S, Monopoli M, Doherty M, Croom O, Engineer L. Person-centered care model in dentistry. BMC Oral Health. 2018 Nov 29;18(1):198. doi: 10.1186/s12903-018-0661-9. PMID: 30497465; PMCID: PMC6267887. |
| Involving key stakeholders for planning of patient-centered research | 1. Chi DL, Milgrom P, Gillette J. Engaging Stakeholders in Patient-Centered Outcomes Research Regarding School-Based Sealant Programs. J Dent Hyg. 2018 Feb;92(1):16-22. PMID: 29500281. |
| Financial hardship screening, cost conversations between dental team & patient | 1. Brown T, Apenteng BA, Opoku ST. Factors associated with cost conversations in oral health care settings. J Am Dent Assoc. 2022 Sep;153(9):829-838. doi: 10.1016/j.adaj.2022.04.003. Epub 2022 May 16. PMID: 35589435. |

# Timeliness

According to “An International Working Definition for Quality of Oral Healthcare” (Righolt et. al, 2019) aims are:

In order to be considered timely, oral healthcare should

• Avoid unnecessary delays in access and utilization of care.

• Prioritize care based on need, not on demand (e.g., employ checkup and recall intervals based on patient’s own risks).

• Implement care coordination between healthcare providers and institutions.

• Prioritize prevention; avoid too early use of restorative and other treatments.

• Implement school-based oral healthcare if appropriate.

Number of titles screened: 75

Number of abstracts screened: 62

Number of full texts screened: 4

| **Possible topics deduced from literature** | **Reference** |
| --- | --- |
| Timely follow up after treatment | Previously covered (old literature screening see Righolt et. al, 2019 |
| Timely dental care | Previously covered (old literature screening see Righolt et. al, 2019 |
| Harmful waiting times/protracted waiting times | Previously covered (old literature screening see Righolt et. al, 2019 |
| Absenteeism from work due to complications of waiting times | Previously covered (old literature screening see Righolt et. al, 2019 |
| Influence affordability of care on timeliness (or combine with access to care) | Previously covered (old literature screening see Righolt et. al, 2019 |
| Availability of dental professionals | Previously covered (old literature screening see Righolt et. al, 2019 |
| Timeliness of guidelines | Previously covered (old literature screening see Righolt et. al, 2019 |
| Timeliness medical prescriptions | Previously covered (old literature screening see Righolt et. al, 2019 |
| Timely advice about oral care | Previously covered (old literature screening see Righolt et. al, 2019 |
| Timeliness in access to care | Previously covered (old literature screening see Righolt et. al, 2019 |
| Timely access to care through health literacy | 1. Zhang X, Liu D, Dong H, Li Y, Zhang Y, Wang X, Zhang B, Bian L. Factors associated with delay in presentation among patients for oral cancer. J Comp Eff Res. 2019 Sep;8(12):1003-1071. doi: 10.2217/cer-2019-0067. Epub 2019 Jul 10. PMID: 31290337. 2. Siddiqi A, Zafar S, Sharma A, Quaranta A. Awareness of Diabetic Patients Regarding the Bidirectional Association between Periodontal Disease and Diabetes Mellitus: A Public Oral Health Concern. J Contemp Dent Pract. 2020 Nov 1;21(11):1270-1274. PMID: 33850074. 3. Adham MM, El Kashlan MK, Abdelaziz WE, Rashad AS. The impact of minimally invasive restorative techniques on perception of dental pain among pregnant women: a randomized controlled clinical trial. BMC Oral Health. 2021 Feb 18;21(1):76. doi: 10.1186/s12903-021-01432-3. PMID: 33602209; PMCID: PMC7891028. |
| Timely access to care through screening | 1. Zhang X, Liu D, Dong H, Li Y, Zhang Y, Wang X, Zhang B, Bian L. Factors associated with delay in presentation among patients for oral cancer. J Comp Eff Res. 2019 Sep;8(12):1003-1071. doi: 10.2217/cer-2019-0067. Epub 2019 Jul 10. PMID: 31290337. |
| Demographic, socioeconomic, environmental determinants of timely access | 1. Tan YR, Tan EH, Jawahir S, Mohd Hanafiah AN, Mohd Yunos MH. Demographic and socioeconomic inequalities in oral healthcare utilisation in Malaysia: evidence from a national survey. BMC Oral Health. 2021 Jan 19;21(1):34. doi: 10.1186/s12903-020-01388-w. PMID: 33468125; PMCID: PMC7816410. 2. Soares ALFH, Ribeiro CCC, Thomaz EBAF, Queiroz RCS, Alves CMC, Ferraro AA, Silva AAM, Bettiol H, Barbieri MA, Saraiva MCP. Socio-environmental determinants of the delay in the first dental visit: results of two population-based cohort studies in Brazil. Braz J Med Biol Res. 2020 Nov 27;54(1):e10161. doi: 10.1590/1414-431X202010161. PMID: 33263609; PMCID: PMC7695448. |
| Interprofessional care, effective & timely referral pathways, knowledge of oral health in other healthcare workers, comprehensive care to reduce unmet health needs, continuity of care | 1. Sorokina AA, Losev FF, Kravchenko IE. Analiz sostoyaniya stomatologicheskoi pomoshchi infektsionnym bol'nym v usloviyakh statsionara [Analysis of the dental care for the contagious patients in the in-patient units]. Stomatologiia (Mosk). 2021;100(6):82-85. Russian. doi: 10.17116/stomat202110006182. PMID: 34953194. 2. Smith M, Stroud C, Low M, Gibson C, Ferguson CA, Broadbent J. Non-traumatic dental presentations at emergency departments in New Zealand. N Z Med J. 2021 Oct 22;134(1544):99-112. PMID: 34695097. 3. Siddiqi A, Zafar S, Sharma A, Quaranta A. Awareness of Diabetic Patients Regarding the Bidirectional Association between Periodontal Disease and Diabetes Mellitus: A Public Oral Health Concern. J Contemp Dent Pract. 2020 Nov 1;21(11):1270-1274. PMID: 33850074. 4. Ran X, Yu M. [Family physicians' knowledge regarding oral disease treatment during pregnancy in Jiading district, Shanghai city]. Shanghai Kou Qiang Yi Xue. 2018 Oct;27(5):504-507. Chinese. PMID: 30680395. 5. Pourat N, Chen X, Lee C, Zhou W, Daniel M, Hoang H, Sharma R, Sim H, Sripipatana A, Nair S. HRSA-funded Health Centers Are an Important Source of Care and Reduce Unmet Needs in Primary Care Services. Med Care. 2019 Dec;57(12):996-1001. doi: 10.1097/MLR.0000000000001206. PMID: 31730569. 6. Pateman KA, Weerakoon AT, Batstone MD, Ford PJ. A culture shock in dental hygiene: Exploring the management of oral health after head and neck cancer. Int J Dent Hyg. 2019 May;17(2):183-191. doi: 10.1111/idh.12376. Epub 2018 Dec 25. PMID: 30506841. 7. Jordão HWT, Coleman HG, Kunzmann AT, McKenna G. The association between erosive toothwear and gastro-oesophageal reflux-related symptoms and disease: A systematic review and meta-analysis. J Dent. 2020 Apr;95:103284. doi: 10.1016/j.jdent.2020.103284. Epub 2020 Jan 30. PMID: 32006670. |
| Covid-19, pandemic considerations of timely access | 1. Sekundo C, Langowski E, Frese C. Influence of COVID-19 Restrictions on Patients' Self-reported Oral Health Care Needs. Oral Health Prev Dent. 2021 Jan 7;19(1):339-343. doi: 10.3290/j.ohpd.b1693919. PMID: 34259426. |
| Timeliness of data (collection) (quality improvement, research, etc.), (implant) registry (data concerning patient, implant, complications, follow-up) to facilitate further treatments | 1. Ruff JC, Herndon JB, Horton RA, Lynch J, Mathwig DC, Leonard A, Aravamudhan K. Developing a caries risk registry to support caries risk assessment and management for children: A quality improvement initiative. J Public Health Dent. 2018 Mar;78(2):134-143. doi: 10.1111/jphd.12253. Epub 2017 Oct 27. PMID: 29077195. 2. Naemi R, Jangi M, Barikani HR, Shahmoradi L. Design and Evaluation of Web-Based Dental Implant Registry (DIR) for Better Clinical Outcomes. Int J Biomater. 2022 Feb 11;2022:7162645. doi: 10.1155/2022/7162645. PMID: 35186089; PMCID: PMC8856821. 3. Chattopadhyay A, Christian B, Gussy M, Masood M, Hegde S, Raichur A, Martin R, Kenny A. Oral health surveillance in Australia: the need for ongoing data to inform public health decision-making. Aust J Prim Health. 2022 Feb;28(1):18-22. doi: 10.1071/PY21001. PMID: 34879900. |
| Legal framework, regulatory barriers to receive timely care | 1. Losev FF, Vagner VD, Sorokina AA. Zakonodatel'naia osnova i normativnoe pravovoe obespechenie okazaniia stomatologicheskoĭ pomoshchi infektsionnym bol'nym [Legislative framework and legal regulation of dental care organization to patients with infectious diseases]. Stomatologiia (Mosk). 2019;98(2):4-7. Russian. doi: 10.17116/stomat2019980214. PMID: 31089111. |
| Willingness of providers to provide care to special needs patients due to perceived lack of knowledge/training | 1. Lim MAWT, Liberali SAC, Calache H, Parashos P, Borromeo GL. Mentoring of oral health professionals is crucial to improving access to care for people with special needs. PLoS One. 2022 Apr 25;17(4):e0266879. doi: 10.1371/journal.pone.0266879. PMID: 35468149; PMCID: PMC9037927. |
| Triaging patients | 1. Lee VHK, Grant CG, Mittermuller BA, Singh S, Weiss B, Edwards JM, Schroth RJ. Association between early childhood oral health impact scale (ECOHIS) scores and pediatric dental surgery wait times. BMC Oral Health. 2020 Oct 17;20(1):285. doi: 10.1186/s12903-020-01263-8. PMID: 33069219; PMCID: PMC7568462. |
| Perceived barriers (special needs patients) -> organization & resources, financial & welfare policies, empathy & accommodation of staff | 1. Kim YJ, Lim YW, Paik HR, Lee JY, Kim SY. What influences use of dental services by the Korean disabled people? The role of perceived barriers in dental care system. Community Dent Health. 2019 May 30;36(2):101-105. doi: 10.1922/CDH_4456Young05. PMID: 31070873. |
| Timely information to patient (regarding treatment) | 1. Kashbour WA, Rousseau NS, Thomason JM, Ellis JS. Provision of information to patients on dental implant treatment: Clinicians' perspectives on the current approaches and future strategies. J Dent. 2018 Sep;76:117-124. doi: 10.1016/j.jdent.2018.07.002. Epub 2018 Jul 9. PMID: 30004003. |

# Efficiency

According to “An International Working Definition for Quality of Oral Healthcare” (Righolt et. al, 2019) aims are:

In order to be considered efficient, oral healthcare should

• Avoid waste of resources, including waste of human resources, time, equipment, dental materials. Such measures will

also minimize the carbon footprint of oral healthcare.

• Prioritize cost-efficient interventions that provide good value-for-money.

• Encourage prevention and discourage expensive restorative care.

• Rely on patients’ oral health needs as the central basis for resource and workforce planning.

• Form an integral part of medicine and discourage the dental-medical divide.

Number of titles screened: 117

Number of abstracts screened: 60

Number of full texts screened: 4

| **Possible topics deduced from literature** | **Reference** |
| --- | --- |
| Waste of time | Previously covered (old literature screening see Righolt et. al, 2019 |
| Waste of resources | Previously covered (old literature screening see Righolt et. al, 2019 |
| Productivity | Previously covered (old literature screening see Righolt et. al, 2019 |
| Waste of materials/supplies/equipment | Previously covered (old literature screening see Righolt et. al, 2019 |
| Technical efficiency | Previously covered (old literature screening see Righolt et. al, 2019 |
| Efficiency of the workforce (role substitution) | Previously covered (old literature screening see Righolt et. al, 2019 |
| Usability | Previously covered (old literature screening see Righolt et. al, 2019 |
| Understanding enablers and barriers to efficiency and acting upon this understanding | 1. Wilson A, Hoang H, Barnett T. Barriers and enablers to skill-mix in the oral health workforce: A systematic review. Community Dent Health. 2021 May 28;38(2):89-99. doi: 10.1922/CDH_00028-2019Wilson11. PMID: 33507647. |
| Integration of primary and oral health care, interprofessional working, sharing information | 1. West JF, King RK. Academic and Community Partnerships: Increasing Access Through Collaborative Care. J Dent Educ. 2019 Feb;83(2 Suppl):S23-S27. doi: 10.21815/JDE.019.039. PMID: 30709936. 2. Robinson FG, Fischbach H, Salisbury J, Stefanik D, Kearney R, Fields HW. Clinical Integration in a Dental School Clinic Through an Enhanced Patient Intake Process. J Dent Educ. 2019 Sep;83(9):1030-1038. doi: 10.21815/JDE.019.105. Epub 2019 May 13. PMID: 31085688. |
| Building connections, partnerships with the community (“promote the sharing of information, facilitate provision of basic diagnostic services, and bring the bidirectional flow of knowledge, training, and skills to one another in a systematic and sustained manner”) | 1. West JF, King RK. Academic and Community Partnerships: Increasing Access Through Collaborative Care. J Dent Educ. 2019 Feb;83(2 Suppl):S23-S27. doi: 10.21815/JDE.019.039. PMID: 30709936. |
| net revenue, no-show rates, total encounters, and number of encounters for with special patient groups (such as young children and pregnant women) | 1. Vander Schaaf EB, Quinonez RB, Cornett AC, Randolph GD, Boggess K, Flower KB. A Pilot Quality Improvement Collaborative to Improve Safety Net Dental Access for Pregnant Women and Young Children. Matern Child Health J. 2018 Feb;22(2):255-263. doi: 10.1007/s10995-017-2397-6. PMID: 29168163. 2. Salkinoja P, Tuononen T, Suominen AL, Lammintakanen J, Raittio E. Oral health care quality as viewed by leading dentists and their superiors - a qualitative study. Acta Odontol Scand. 2022 Jan;80(1):38-43. doi: 10.1080/00016357.2021.1936161. Epub 2021 Jun 21. PMID: 34153210. 3. Kim SJ, Patel N, Xu J, Huot V, Sonani H, Magnuson BE. Exploratory analysis to enhance operational efficiency of new patient screenings. J Dent Educ. 2021 Apr;85(4):555-561. doi: 10.1002/jdd.12489. Epub 2020 Nov 16. PMID: 33197040. |
| Survival time = efficiency (i.e. sustainability, etc.?) | 1. Salkinoja P, Tuononen T, Suominen AL, Lammintakanen J, Raittio E. Oral health care quality as viewed by leading dentists and their superiors - a qualitative study. Acta Odontol Scand. 2022 Jan;80(1):38-43. doi: 10.1080/00016357.2021.1936161. Epub 2021 Jun 21. PMID: 34153210. |
| Efficient data collection, storage & use (e.g. big data) | 1. Nanayakkara S, Zhou X, Spallek H. Impact of big data on oral health outcomes. Oral Dis. 2019 Jul;25(5):1245-1252. doi: 10.1111/odi.13007. Epub 2018 Dec 28. PMID: 30474902. |
| Oral health problems as a reason for absenteeism and presenteeism (-> how does this relate to timely care? -> workers go to work with oral health problems and don’t receive care -> care is not timely?) | 1. Lima RB, Buarque A. Oral health in the context of prevention of absenteeism and presenteeism in the workplace. Rev Bras Med Trab. 2019 Dec 1;17(4):594-604. doi: 10.5327/Z1679443520190397. PMID: 32685760; PMCID: PMC7363255. |
| Education | 1. Kennedy EN, Alex White B, Weintraub JA, Moss ME, Jordan SL, Quick KK, Ticku S, Huang B, Douglass C. Collaborating and teaching a synchronous, multi-university, virtual course: Health policy and access to dental care. J Dent Educ. 2021 Sep;85(9):1536-1542. doi: 10.1002/jdd.12650. Epub 2021 May 31. PMID: 34060648. |

# Equitability

According to “An International Working Definition for Quality of Oral Healthcare” (Righolt et. al, 2019) aims are:

In order to be considered equitable, oral healthcare should

• Not vary in quality and availability because of gender, ethnicity, cultural background, religion and belief, geographic

location, and/or socioeconomic status.

• Provide equitable access to all.

• Not be compromised in terms of either quality or availability on the basis of a patient’s health status.

• Address inequities in oral health service design, planning, and commissioning.

• Incorporate equitability in the design of policy and clinical practice guidelines.

Number of titles screened: 348

Number of abstracts screened: 337

Number of full texts screened: 8

| **Possible topics deduced from literature** | **Reference** |
| --- | --- |
| ethnicity disparities | Previously covered (old literature screening see Righolt et. al, 2019 |
| Access to care: costs | Previously covered (old literature screening see Righolt et. al, 2019 |
| Geographical disparities | Previously covered (old literature screening see Righolt et. al, 2019 |
| Socio-economic inequality | Previously covered (old literature screening see Righolt et. al, 2019 |
| Health literacy | Previously covered (old literature screening see Righolt et. al, 2019 |
| Cultural disparities/cultural competence | Previously covered (old literature screening see Righolt et. al, 2019 |
| Inequality due to education | Previously covered (old literature screening see Righolt et. al, 2019 |
| Barriers to seek oral care | Previously covered (old literature screening see Righolt et. al, 2019 |
| Interprofessional care to reduce disparities | Previously covered (old literature screening see Righolt et. al, 2019 |
| Disability and medical related disparities | Previously covered (old literature screening see Righolt et. al, 2019 |
| Disparity due to age | Previously covered (old literature screening see Righolt et. al, 2019 |
| Insurance  (e.g. Medicaid vs. private,  China: urban vs rural vs no insurance) / safety net | 1. Zivkovic N, Aldossri M, Gomaa N, Farmer JW, Singhal S, Quiñonez C, Ravaghi V. Providing dental insurance can positively impact oral health outcomes in Ontario. BMC Health Serv Res. 2020 Feb 17;20(1):124. doi: 10.1186/s12913-020-4967-3. Erratum in: BMC Health Serv Res. 2021 Mar 12;21(1):224. PMID: 32066434; PMCID: PMC7027064. 2. Wilson FA, Wang Y, Borrell LN, Bae S, Stimpson JP. Disparities in oral health by immigration status in the United States. J Am Dent Assoc. 2018 Jun;149(6):414-421.e3. doi: 10.1016/j.adaj.2018.01.024. Epub 2018 Apr 1. PMID: 29615187. 3. Tranby EP, Frantsve-Hawley J, Minter-Jordan M, Thommes J, Jacob M, Monopoli M, Okunev I, Boynes SG. A cross-sectional analysis of oral health care spending over the life span in commercial- and Medicaid-insured populations. J Am Dent Assoc. 2022 Feb;153(2):101-109.e11. doi: 10.1016/j.adaj.2021.07.028. Epub 2021 Nov 10. PMID: 34772476. 4. Stephens R, White BA. Oral health status of adults in North Carolina assisted living facilities. J Public Health Dent. 2019 Sep;79(3):198-206. doi: 10.1111/jphd.12310. Epub 2019 Feb 11. PMID: 30741490. 5. Serban N, Anderson A, Oberst G, Edupuganti N, Ramachandran R, Solipuram SR, Lu T. Assessment of Dentist Participation in Public Insurance Programs for Children in the US. JAMA Netw Open. 2022 Jul 1;5(7):e2221444. doi: 10.1001/jamanetworkopen.2022.21444. PMID: 35816300; PMCID: PMC9274318. 6. Semprini J, Wehby G. Impact of Medicaid dental coverage expansion on self-reported tooth loss in low-income adults. J Am Dent Assoc. 2022 Sep;153(9):839-847. doi: 10.1016/j.adaj.2022.03.003. Epub 2022 May 3. PMID: 35513904; PMCID: PMC9427673. 7. Piedrasanta FC, Boyd LD, Vineyard J, LaSpina L. Oral Health Knowledge, Acculturation and Utilization of Oral Health Services among a Hispanic and Latino Population. J Dent Hyg. 2021 Oct;95(5):22-31. PMID: 34654712. 8. Patel N, Fils-Aime R, Li CH, Lin M, Robison V. Prevalence of Past-Year Dental Visit Among US Adults Aged 50 Years or Older, With Selected Chronic Diseases, 2018. Prev Chronic Dis. 2021 Apr 29;18:E40. doi: 10.5888/pcd18.200576. PMID: 33914679; PMCID: PMC8091943. 9. Northridge ME, Kumar A, Kaur R. Disparities in Access to Oral Health Care. Annu Rev Public Health. 2020 Apr 2;41:513-535. doi: 10.1146/annurev-publhealth-040119-094318. Epub 2020 Jan 3. PMID: 31900100; PMCID: PMC7125002. 10. Nguyen VH, Lin SC, Cappelli DP, Nair S. The association between dental, general, and mental health status among underserved and vulnerable populations served at health centers in the US. J Public Health Dent. 2018 Dec;78(1):41-48. doi: 10.1111/jphd.12234. Epub 2017 Jul 18. PMID: 28719064. 11. Li C, Yao NA. Socio-Economic Disparities in Dental Health and Dental Care Utilisation Among Older Chinese. Int Dent J. 2021 Feb;71(1):67-75. doi: 10.1111/idj.12600. Epub 2021 Jan 12. PMID: 33616055; PMCID: PMC9275339. 12. Lee H, Tranby E, Shi L. Dental Visits during Pregnancy: Pregnancy Risk Assessment Monitoring System Analysis 2012-2015. JDR Clin Trans Res. 2022 Oct;7(4):379-388. doi: 10.1177/23800844211028541. Epub 2021 Jul 29. PMID: 34323108. 13. Kim PC, Zhou W, McCoy SJ, McDonough IK, Burston B, Ditmyer M, Shen JJ. Factors Associated with Preventable Emergency Department Visits for Nontraumatic Dental Conditions in the U.S. Int J Environ Res Public Health. 2019 Sep 30;16(19):3671. doi: 10.3390/ijerph16193671. PMID: 31574897; PMCID: PMC6801501. 14. Kim NH, Kawachi I. Insurance coverage expansion and inequalities in unmet oral healthcare needs in Korea: Repeated cross-sectional analysis, 2007-2015. Community Dent Oral Epidemiol. 2021 Jun;49(3):232-239. doi: 10.1111/cdoe.12594. Epub 2020 Nov 11. PMID: 33179344. 15. Kim NH, Kawachi I. Did the Expansion of Insurance Coverage for Oral Health Reduce Self-reported Oral Health Inequalities in Korea? Results of Repeated Cross-Sectional Analysis, 2007-2015. J Epidemiol. 2020 Dec 5;30(12):537-541. doi: 10.2188/jea.JE20190119. Epub 2019 Dec 7. PMID: 31813892; PMCID: PMC7661332. 16. Kimia R, Butler PD, Guajardo I, Magee L, Lowe K, Scott M, Wes A, Jackson OA. Sociodemographic Factors That Influence the Choice to Pursue Nasoalveolar Molding: One Pediatric Hospital's Experience. Cleft Palate Craniofac J. 2020 Sep;57(9):1069-1077. doi: 10.1177/1055665620936056. Epub 2020 Jul 3. PMID: 32618203. 17. Kieffer EC, Goold SD, Buchmueller T, Nalliah R, Beathard E, Kirch MA, Solway E, Tipirneni R, Clark SJ, Haggins AN, Patel MR, Ayanian JZ. Beneficiaries' perspectives on improved oral health and its mediators after Medicaid expansion in Michigan: a mixed methods study. J Public Health Dent. 2022 Jan;82(1):11-21. doi: 10.1111/jphd.12447. Epub 2021 Mar 22. PMID: 33754344. 18. Jang YE, Kim CB, Kim NH. Influence of dental insurance coverage on access to preventive periodontal care in middle-aged and elderly populations: analysis of representative Korean Community Health Survey Data (2011-2015). Int Dent J. 2019 Dec;69(6):445-453. doi: 10.1111/idj.12488. Epub 2019 May 22. PMID: 31115042; PMCID: PMC9379082. 19. Jaffe K, Choi J, Hayashi K, Milloy MJ, Richardson L. A paradox of need: Gaps in access to dental care among people who use drugs in Canada's publicly funded healthcare system. Health Soc Care Community. 2021 Nov;29(6):1799-1806. doi: 10.1111/hsc.13289. Epub 2021 Jan 25. PMID: 33491849; PMCID: PMC8310531. 20. Ito K, Cable N, Yamamoto T, Suzuki K, Kondo K, Osaka K, Tsakos G, Watt RG, Aida J. Wider Dental Care Coverage Associated with Lower Oral Health Inequalities: A Comparison Study between Japan and England. Int J Environ Res Public Health. 2020 Jul 31;17(15):5539. doi: 10.3390/ijerph17155539. PMID: 32751843; PMCID: PMC7432332. 21. Hewlett SA, Anto F, Blankson PK, Tormeti D, Ayettey-Adamafio M, Bayitse P, Danso-Appiah T, Amoah AG. Periodontitis prevalence and severity in an African population: A cross-sectional study in the Greater Accra Region of Ghana. J Periodontol. 2022 May;93(5):732-744. doi: 10.1002/JPER.21-0329. Epub 2022 Jan 3. PMID: 34724216. 22. Gupta A, Feldman S, Perkins RB, Stokes A, Sankar V, Villa A. Predictors of dental care use, unmet dental care need, and barriers to unmet need among women: results from NHANES, 2011 to 2016. J Public Health Dent. 2019 Dec;79(4):324-333. doi: 10.1111/jphd.12334. Epub 2019 Aug 13. PMID: 31407356. 23. Guarnizo-Herreño CC, Watt RG, Garzón-Orjuela N, Suárez-Zúñiga E, Tsakos G. Health insurance and education: major contributors to oral health inequalities in Colombia. J Epidemiol Community Health. 2019 Aug;73(8):737-744. doi: 10.1136/jech-2018-212049. Epub 2019 May 16. PMID: 31097482. 24. Griffin SO, Thornton-Evans G, Wei L, Griffin PM. Disparities in Dental Use and Untreated Caries Prevalence by Income. JDR Clin Trans Res. 2021 Apr;6(2):234-241. doi: 10.1177/2380084420934746. Epub 2020 Jun 25. PMID: 32585114., 25. Freire DEWG, Freire AR, Lucena EHG, Cavalcanti YW. Oral health access in Brazil: analysis of inequities and non-access from the service user's perspective, according to the National Primary Care Access and Quality Improvement Program, 2014 and 2018. Epidemiol Serv Saude. 2021 Sep 3;30(3):e2020444. English, Portuguese. doi: 10.1590/S1679-49742021000300016. PMID: 34495041. 26. Cheng J, Gregorich SE, Gansky SA, Fisher-Owens SA, Kottek AM, White JM, Mertz EA. Constructing Matched Groups in Dental Observational Health Disparity Studies for Causal Effects. JDR Clin Trans Res. 2020 Jan;5(1):82-91. doi: 10.1177/2380084419830655. Epub 2019 Mar 11. PMID: 30931723; PMCID: PMC6918038. |
| “Migration vs. native”, also migration history  (Also barriers due to lacking provider training for migrant patients, language barriers) | 1. Zhang H, Zeng X, Jiang Y, Xu W, Wang X, Li C, Zhang Y, Liu Y, Wang Y. The disparity in caries and sealants between migrant and native children in Shanghai: A cross-sectional study. Int J Dent Hyg. 2020 Feb;18(1):84-91. doi: 10.1111/idh.12411. Epub 2019 Aug 5. PMID: 31380599; PMCID: PMC7004011. 2. Xi S, Song Y, Li X, Li M, Lu Z, Yang Y, Wang Y. Local-Migrant Gaps in Healthcare Utilization Between Older Migrants and Local Residents in China. J Am Geriatr Soc. 2020 Jul;68(7):1560-1567. doi: 10.1111/jgs.16421. Epub 2020 Mar 28. PMID: 32222105. 3. Wilson FA, Wang Y, Borrell LN, Bae S, Stimpson JP. Disparities in oral health by immigration status in the United States. J Am Dent Assoc. 2018 Jun;149(6):414-421.e3. doi: 10.1016/j.adaj.2018.01.024. Epub 2018 Apr 1. PMID: 29615187. 4. Wiener RC, Findley PA, Shen C, Dwibedi N, Sambamoorthi U. Acculturation and dental sealant use among US children. Community Dent Oral Epidemiol. 2022 Oct;50(5):367-374. doi: 10.1111/cdoe.12678. Epub 2021 Jul 12. PMID: 34251694; PMCID: PMC8752649. 5. Sano Y, Antabe R. Regular Dental Care Utilization: The Case of Immigrants in Ontario, Canada. J Immigr Minor Health. 2022 Feb;24(1):162-169. doi: 10.1007/s10903-021-01265-w. Epub 2021 Aug 28. PMID: 34453263. 6. Rodriguez-Alvarez E, Borrell LN, Marañon E, Lanborena N. Immigrant Status and Ethnic Inequities in Dental Caries in Children: Bilbao, Spain. Int J Environ Res Public Health. 2022 Apr 8;19(8):4487. doi: 10.3390/ijerph19084487. PMID: 35457355; PMCID: PMC9032871. 7. Raskin SE, Rasnick R, Kohlmann T, Zanin M, Bilodeau J, Akinkugbe A. Oral Health, Health Service Utilization, and Age at Arrival to the U.S. among Safety Net Patients. Int J Environ Res Public Health. 2022 Jan 28;19(3):1477. doi: 10.3390/ijerph19031477. PMID: 35162501; PMCID: PMC8835091. 8. Pichemin C, Boyer E, Jarno P, Bertaud V, Meuric V, Couatarmanach A. Oral Care Needs Amongst Disadvantaged Migrants in France. Int Dent J. 2022 Aug;72(4):559-564. doi: 10.1016/j.identj.2021.12.002. Epub 2022 Mar 9. PMID: 35279328; PMCID: PMC9381369. 9. Nishioka D, Ueno K, Kino S, Aida J, Kondo N. Sociodemographic inequities in dental care utilisation among governmental welfare recipients in Japan: a retrospective cohort study. Int J Equity Health. 2021 Jun 16;20(1):141. doi: 10.1186/s12939-021-01473-8. PMID: 34134717; PMCID: PMC8207738. 10. Hollederer A, Wildner M. Ungedeckter medizinischer Versorgungsbedarf in Deutschland: Ergebnisse im EU-SILC-Survey 2005 bis 2014 [Unmet Medical Need in Germany: Analyses of EU-SILC-Survey from 2005 to 2014]. Dtsch Med Wochenschr. 2019 Jan;144(1):e1-e11. German. doi: 10.1055/a-0671-4605. Epub 2019 Jan 2. PMID: 30602179. 11. Diamanti I, Berdouses ED, Kavvadia K, Arapostathis KN, Polychronopoulou A, Oulis CJ. Dental Caries Prevalence and Experience (ICDAS II Criteria) of 5-, 12- and 15-Year-Old Children and Adolescents with an Immigrant Background in Greece, Compared with the Host Population: A Cross-Sectional Study. Int J Environ Res Public Health. 2021 Dec 21;19(1):14. doi: 10.3390/ijerph19010014. PMID: 35010274; PMCID: PMC8751141. 12. Carrasco-Sanz A, Leiva-Gea I, Martin-Alvarez L, Del Torso S, van Esso D, Hadjipanayis A, Kadir A, Ruiz-Canela J, Perez-Gonzalez O, Grossman Z. Migrant children's health problems, care needs, and inequalities: European primary care paediatricians' perspective. Child Care Health Dev. 2018 Mar;44(2):183-187. doi: 10.1111/cch.12538. Epub 2017 Nov 21. PMID: 29159977. 13. Campus G, Cocco F, Strohmenger L, Wolf TG, Balian A, Arghittu A, Cagetti MG. Inequalities in caries among pre-school Italian children with different background. BMC Pediatr. 2022 Jul 23;22(1):443. doi: 10.1186/s12887-022-03470-4. PMID: 35869462; PMCID: PMC9308358. 14. Alhozgi A, Feine JS, Tanwir F, Shrivastava R, Galarneau C, Emami E. Rural-urban disparities in patient satisfaction with oral health care: a provincial survey. BMC Oral Health. 2021 May 15;21(1):261. doi: 10.1186/s12903-021-01613-0. PMID: 33992110; PMCID: PMC8122552. |
| Socioeconomic status of children / their family, children living conditions (e.g. maternal / parental education, care giver: parents, grandparents, etc., presence of caregiver)  Values and attitudes of parents  Caregiver involvement in brushing,  Parents oral health practices, parents alcohol consumption,  Childhood obesity | 1. Yun Q, Liu M, Zhao M, Chen W, Zhang H, Hou W, Chang C. Ten-year changes in children's oral health disparities: Findings from the 3rd and 4th oral health surveys in Beijing, China. Community Dent Oral Epidemiol. 2021 Dec;49(6):513-521. doi: 10.1111/cdoe.12682. Epub 2021 Jul 24. PMID: 34302379; PMCID: PMC8597119. 2. Williams SE, Rainchuso L, Boyd LD, Vineyard J. Oral Health Considerations of Children: Grandparent Caregiver Perceptions, Behaviors, and Knowledge. Matern Child Health J. 2021 May;25(5):759-768. doi: 10.1007/s10995-020-03087-y. Epub 2020 Nov 21. PMID: 33222106. 3. Watt RG, Mathur MR, Aida J, Bönecker M, Venturelli R, Gansky SA. Oral Health Disparities in Children: A Canary in the Coalmine? Pediatr Clin North Am. 2018 Oct;65(5):965-979. doi: 10.1016/j.pcl.2018.05.006. PMID: 30213357. 4. Su H, Zhang Y, Qian W, Shi H. Impact of Behavioural Factors and Living Conditions on Dental Caries Among Pupils from Shanghai and Jiangxi Province in China: A School-based Cross-sectional Study. Oral Health Prev Dent. 2019;17(6):557-565. doi: 10.3290/j.ohpd.a42742. PMID: 31268050. 5. Silva ELMSD, Góes PSA, Vasconcelos MMVB, Jamelli SR, Eickmann SH, Melo MMDC, Lima MC. Oral health care for children and adolescents with cerebral palsy: perceptions of parents and caregivers. Cien Saude Colet. 2020 Oct;25(10):3773-3784. Portuguese, English. doi: 10.1590/1413-812320202510.27972018. Epub 2019 Feb 13. PMID: 32997011. 6. Nelson CI, Wright CD, Brumbaugh JT, Neiswanger K, Crout RJ, Lilly CL, Marazita ML, McNeil DW. Predictors of use of dental care by children in north-central Appalachia in the USA. PLoS One. 2021 Jul 22;16(7):e0250488. doi: 10.1371/journal.pone.0250488. PMID: 34292949; PMCID: PMC8297786. 7. Najjar S, Nasim M, Al-Nasser L, Masuadi E. The Impact of Socio-Economic and Home Environmental Factors on Oral Health-Related Quality of Life Among Children Aged 11-14. Medicina (Kaunas). 2019 Oct 31;55(11):722. doi: 10.3390/medicina55110722. PMID: 31683702; PMCID: PMC6915500. 8. Martin MA, Avenetti D, Lee HH, Nordgren R, Berbaum ML, Edomwande Y, Cui L, Sandoval A. Community health worker intervention to improve tooth brushing in young children: Results from a cluster randomized controlled trial. Community Dent Oral Epidemiol. 2022 Jun 29. doi: 10.1111/cdoe.12768. Epub ahead of print. PMID: 35766288. 9. Marquillier T, Lombrail P, Azogui-Lévy S. Inégalités sociales de santé orale et caries précoces de l’enfant : comment prévenir efficacement ? Une revue de portée des prédicteurs de la maladie [Social inequalities in oral health and early childhood caries: How can they be effectively prevented? A scoping review of disease predictors]. Rev Epidemiol Sante Publique. 2020 Aug;68(4):201-214. French. doi: 10.1016/j.respe.2020.06.004. Epub 2020 Jul 4. PMID: 32631663. 10. Hong CL, Broadbent JM, Thomson WM, Poulton R. The Dunedin Multidisciplinary Health and Development Study: Oral health findings and their implications. J R Soc N Z. 2020;50(1):35-46. doi: 10.1080/03036758.2020.1716816. Epub 2020 Feb 4. PMID: 32226196; PMCID: PMC7100605. 11. Diamanti I, Berdouses ED, Kavvadia K, Arapostathis KN, Reppa C, Sifakaki M, Panagopoulou O, Polychronopoulou A, Oulis CJ. Caries prevalence and caries experience (ICDAS II criteria) of 5-, 12- and 15-year-old Greek children in relation to socio-demographic risk indicators. Trends at the national level in a period of a decade. Eur Arch Paediatr Dent. 2021 Aug;22(4):619-631. doi: 10.1007/s40368-020-00599-7. Epub 2021 Jan 10. PMID: 33423209. 12. Dahlan R, Ghazal E, Saltaji H, Salami B, Amin M. Impact of social support on oral health among immigrants and ethnic minorities: A systematic review. PLoS One. 2019 Jun 20;14(6):e0218678. doi: 10.1371/journal.pone.0218678. PMID: 31220165; PMCID: PMC6586326. 13. Crocombe LA, Allen P, Bettiol S, Babo Soares LF. Parental Education Level and Dental Caries in School Children Living in Dili, Timor-Leste. Asia Pac J Public Health. 2018 Mar;30(2):128-136. doi: 10.1177/1010539517753875. Epub 2018 Jan 23. PMID: 29359603. 14. Como DH, Stein Duker LI, Polido JC, Cermak SA. The Persistence of Oral Health Disparities for African American Children: A Scoping Review. Int J Environ Res Public Health. 2019 Feb 27;16(5):710. doi: 10.3390/ijerph16050710. PMID: 30818846; PMCID: PMC6427601. 15. Berdahl T, Biener A, McCormick MC, Guevara JP, Simpson L. Annual Report on Children's Healthcare: Healthcare Access and Utilization by Obesity Status in the United States. Acad Pediatr. 2020 Mar;20(2):175-187. doi: 10.1016/j.acap.2019.11.020. Epub 2019 Dec 13. PMID: 31843708. |
| Disparities in community connection, community connection to overcome disparities, community health programs | 1. West JF, King RK. Academic and Community Partnerships: Increasing Access Through Collaborative Care. J Dent Educ. 2019 Feb;83(2 Suppl):S23-S27. doi: 10.21815/JDE.019.039. PMID: 30709936. 2. Tian N, Kobau R, Zack MM, Greenlund KJ. Barriers to and Disparities in Access to Health Care Among Adults Aged ≥18 Years with Epilepsy - United States, 2015 and 2017. MMWR Morb Mortal Wkly Rep. 2022 May 27;71(21):697-702. doi: 10.15585/mmwr.mm7121a1. PMID: 35617131; PMCID: PMC9153461. 3. Monopoli M, Johnson IB. Undermining racism: A road to promoting equity in oral health. J Public Health Dent. 2022 Mar;82 Suppl 1(Suppl 1):123-127. doi: 10.1111/jphd.12511. PMID: 35726464; PMCID: PMC9545495. 4. Chalmers NI, Wislar JS, Hall M, Thurm C, Ng MW. Trends in Pediatric Dental Care Use. Dent Clin North Am. 2018 Apr;62(2):295-317.e12. doi: 10.1016/j.cden.2017.11.008. PMID: 29478459. |
| Vulnerable groups (e.g. exposure to intimate partner violence) | 1. Weijs C, Lang R, Lorenzetti DL, Milaney K, Figueiredo R, Smith LB, McLaren L. The Relation Between Exposure to Intimate Partner Violence and Childhood Dental Decay: A Scoping Review to Identify Novel Public Health Approaches to Early Intervention. J Can Dent Assoc. 2019 May;84:j5. PMID: 31199729. |
| Importance of research regarding inequalities / inequity, research design (i.e. what measures are used for example to define SES) | 1. van Meijeren-van Lunteren AW, You Y, Raat H, Wolvius EB, Kragt L. Caries Preventive Interventions and Oral Health Inequalities: A Scoping Review. JDR Clin Trans Res. 2022 Jul 31:23800844221109116. doi: 10.1177/23800844221109116. Epub ahead of print. PMID: 35912710. 2. Singh A, Peres MA, Watt RG. The Relationship between Income and Oral Health: A Critical Review. J Dent Res. 2019 Jul;98(8):853-860. doi: 10.1177/0022034519849557. Epub 2019 May 15. PMID: 31091113. |
| Equitable policies, funding | 1. Uguru N, Onwujekwe O, Ogu UU, Uguru C. Access to Oral health care: a focus on dental caries treatment provision in Enugu Nigeria. BMC Oral Health. 2020 May 19;20(1):145. doi: 10.1186/s12903-020-01135-1. PMID: 32429976; PMCID: PMC7236454. 2. Kum SS, Northridge ME, Metcalf SS. Using focus groups to design systems science models that promote oral health equity. BMC Oral Health. 2018 Jun 4;18(1):99. doi: 10.1186/s12903-018-0560-0. PMID: 29866084; PMCID: PMC5987593. |
| Gender / sex | 1. Su S, Lipsky MS, Licari FW, Hung M. Comparing oral health behaviours of men and women in the United States. J Dent. 2022 Jul;122:104157. doi: 10.1016/j.jdent.2022.104157. Epub 2022 May 8. PMID: 35545161. 2. Reisine S, Schensul JJ, Li J, Grady J, Ha T, Lalla R. Gingivitis and plaque scores among residents of low-income senior housing. Gerodontology. 2020 Mar;37(1):2-10. doi: 10.1111/ger.12451. Epub 2019 Nov 27. Erratum in: Gerodontology. 2020 Sep;37(3):312-313. PMID: 31774201; PMCID: PMC7012672. 3. Olofsson H, Ulander EL, Gustafson Y, Hörnsten C. Association between socioeconomic and health factors and edentulism in people aged 65 and older - a population-based survey. Scand J Public Health. 2018 Nov;46(7):690-698. doi: 10.1177/1403494817717406. Epub 2017 Aug 3. PMID: 28768459. 4. Nishioka D, Ueno K, Kino S, Aida J, Kondo N. Sociodemographic inequities in dental care utilisation among governmental welfare recipients in Japan: a retrospective cohort study. Int J Equity Health. 2021 Jun 16;20(1):141. doi: 10.1186/s12939-021-01473-8. PMID: 34134717; PMCID: PMC8207738. 5. Najjar S, Nasim M, Al-Nasser L, Masuadi E. The Impact of Socio-Economic and Home Environmental Factors on Oral Health-Related Quality of Life Among Children Aged 11-14. Medicina (Kaunas). 2019 Oct 31;55(11):722. doi: 10.3390/medicina55110722. PMID: 31683702; PMCID: PMC6915500. 6. Meisha DE, Alsolami AM, Alharbi GM. Social determinants of seeking emergency and routine dental care in Saudi Arabia during the COVID-19 pandemic. BMC Oral Health. 2021 Apr 26;21(1):212. doi: 10.1186/s12903-021-01577-1. PMID: 33902535; PMCID: PMC8074701. 7. Martin MA, Collazo GR, Frese WA, Glassgow AE. Oral Health Problems and Solutions in High-Risk Children and Young Adults. J Dent Child (Chic). 2018 Sep 15;85(3):125-132. PMID: 30869589. 8. Liu Y, Yu Y, Nickel JC, Iwasaki LR, Duan P, Simmer-Beck M, Brown L. Gender differences in the association of periodontitis and type 2 diabetes. Int Dent J. 2018 Dec;68(6):433-440. doi: 10.1111/idj.12399. Epub 2018 May 22. PMID: 29786140; PMCID: PMC9379021. 9. Lipsky MS, Su S, Crespo CJ, Hung M. Men and Oral Health: A Review of Sex and Gender Differences. Am J Mens Health. 2021 May-Jun;15(3):15579883211016361. doi: 10.1177/15579883211016361. PMID: 33993787; PMCID: PMC8127762. 10. Limpuangthip N, Purnaveja S, Somkotra T. Predisposing and enabling factors associated with public denture service utilization among older Thai people: a cross-sectional population-based study. BMC Oral Health. 2019 Oct 15;19(1):220. doi: 10.1186/s12903-019-0923-1. PMID: 31615485; PMCID: PMC6794786. 11. Gupta N, Vujicic M, Yarbrough C, Harrison B. Disparities in untreated caries among children and adults in the U.S., 2011-2014. BMC Oral Health. 2018 Mar 6;18(1):30. doi: 10.1186/s12903-018-0493-7. PMID: 29510696; PMCID: PMC5840689. 12. Freire DEWG, Freire AR, Lucena EHG, Cavalcanti YW. Oral health access in Brazil: analysis of inequities and non-access from the service user's perspective, according to the National Primary Care Access and Quality Improvement Program, 2014 and 2018. Epidemiol Serv Saude. 2021 Sep 3;30(3):e2020444. English, Portuguese. doi: 10.1590/S1679-49742021000300016. PMID: 34495041. 13. Elias-Boneta AR, Ramirez K, Rivas-Tumanyan S, Murillo M, Toro MJ. Prevalence of gingivitis and calculus in 12-year-old Puerto Ricans: a cross-sectional study. BMC Oral Health. 2018 Jan 19;18(1):13. doi: 10.1186/s12903-017-0471-5. PMID: 29351752; PMCID: PMC5775617. 14. Celeste RK, Fritzell J. Do socioeconomic inequalities in pain, psychological distress and oral health increase or decrease over the life course? Evidence from Sweden over 43 years of follow-up. J Epidemiol Community Health. 2018 Feb;72(2):160-167. doi: 10.1136/jech-2017-209123. Epub 2017 Nov 24. PMID: 29175868; PMCID: PMC5800356. |
| Institutionalized living (e.g. size of facility as a factor influencing oral health) | 1. Stephens R, White BA. Oral health status of adults in North Carolina assisted living facilities. J Public Health Dent. 2019 Sep;79(3):198-206. doi: 10.1111/jphd.12310. Epub 2019 Feb 11. PMID: 30741490. 2. Sifuentes AMF, Lapane KL. ORAL HEALTH IN NURSING HOMES: WHAT WE KNOW AND WHAT WE NEED TO KNOW. J Nurs Home Res Sci. 2020;6:1-5. PMID: 32524062; PMCID: PMC7286629. 3. Breneol S, Rose H, Brown M, Andreou P, Bishop A, Mitchell C. Exploring the health care utilization of children and youth in the care of child welfare: a retrospective matched cohort study. Fam Pract. 2022 May 28;39(3):360-366. doi: 10.1093/fampra/cmab160. PMID: 34849731. |
| Diet (e.g. as a part of a systems approach to socioeconomic oral health inequities i.e. socioeconomic conditions and nutrition) | 1. Stein C, Cunha-Cruz J, Hugo FN. Is dietary pattern a mediator of the relationship between socioeconomic status and dental caries? Clin Oral Investig. 2021 Sep;25(9):5441-5447. doi: 10.1007/s00784-021-03852-5. Epub 2021 Apr 7. PMID: 33825968. 2. Kyoon-Achan G, Schroth RJ, DeMaré D, Sturym M, Edwards JM, Sanguins J, Campbell R, Chartrand F, Bertone M, Moffatt MEK. First Nations and Metis peoples' access and equity challenges with early childhood oral health: a qualitative study. Int J Equity Health. 2021 Jun 7;20(1):134. doi: 10.1186/s12939-021-01476-5. PMID: 34098968; PMCID: PMC8183050. 3. Ballo L, Arheiam A, Marhazlinda J. Determinants of caries experience and the impact on the OHRQOL of 6-year-old Libyan children: a cross-sectional survey. BMC Oral Health. 2021 Jun 25;21(1):320. doi: 10.1186/s12903-021-01681-2. PMID: 34172041; PMCID: PMC8234736. |
| Screening reduces disparities | 1. Shin BM, Heo JS, Ryu JI. An Investigation of the Association between Health Screening and Dental Scaling in Korea. Int J Environ Res Public Health. 2021 Apr 18;18(8):4294. doi: 10.3390/ijerph18084294. PMID: 33919535; PMCID: PMC8073085. |
| Disparities in safe water / fluoridated water / public health measures  Disparity reduction through water fluoridation, sealant prevalence | 1. Roberto LL, Silveira MF, de Paula AMB, Ferreira E Ferreira E, Martins AMEBL, Haikal DS. Contextual and individual determinants of tooth loss in adults: a multilevel study. BMC Oral Health. 2020 Mar 17;20(1):73. doi: 10.1186/s12903-020-1057-1. PMID: 32183780; PMCID: PMC7076961. 2. Meyer J, Margaritis V, Mendelsohn A. Consequences of community water fluoridation cessation for Medicaid-eligible children and adolescents in Juneau, Alaska. BMC Oral Health. 2018 Dec 13;18(1):215. doi: 10.1186/s12903-018-0684-2. PMID: 30545358; PMCID: PMC6293551. 3. Matsuo G, Aida J, Osaka K, Rozier RG. Effects of Community Water Fluoridation on Dental Caries Disparities in Adolescents. Int J Environ Res Public Health. 2020 Mar 19;17(6):2020. doi: 10.3390/ijerph17062020. PMID: 32204333; PMCID: PMC7175225. 4. Lin M, Griffin SO, Park S, Li C, Robison V, Espinoza L. Associations between Household Water Fluoridation Status and Plain Tap or Bottled Water Consumption. JDR Clin Trans Res. 2021 Oct;6(4):440-447. doi: 10.1177/2380084420960419. Epub 2020 Sep 17. PMID: 32940115; PMCID: PMC8938999. 5. Khalid I, Chandrupatla SG, Kaye E, Scott T, Sohn W. Dental Sealant Prevalence Among Children with Special Health Care Needs: National Health and Nutrition Examination Survey (NHANES) 2013 to 2014. Pediatr Dent. 2019 May 15;41(3):186-190. PMID: 31171069. 6. Jeon JE, Lim AR, Park HA, Ryu JI. Does the Registered Dentists' Program Alleviate the Socioeconomic Gap in the Use of Dental Sealants? Int J Environ Res Public Health. 2020 Oct 26;17(21):7828. doi: 10.3390/ijerph17217828. PMID: 33114677; PMCID: PMC7662614. 7. Hobbs M, Wade A, Jones P, Marek L, Tomintz M, Sharma K, McCarthy J, Mattingley B, Campbell M, Kingham S. Area-level deprivation, childhood dental ambulatory sensitive hospitalizations and community water fluoridation: evidence from New Zealand. Int J Epidemiol. 2020 Jun 1;49(3):908-916. doi: 10.1093/ije/dyaa043. PMID: 32347945. 8. Goldfeld S, Francis KL, Hoq M, Do L, O'Connor E, Mensah F. The Impact of Policy Modifiable Factors on Inequalities in Rates of Child Dental Caries in Australia. Int J Environ Res Public Health. 2019 Jun 3;16(11):1970. doi: 10.3390/ijerph16111970. PMID: 31163687; PMCID: PMC6604007. 9. Do LG, Ha DH, Roberts-Thomson KF, Jamieson L, Peres MA, Spencer AJ. Race- and Income-Related Inequalities in Oral Health in Australian Children by Fluoridation Status. JDR Clin Trans Res. 2018 Apr;3(2):170-179. doi: 10.1177/2380084417751350. Epub 2018 Jan 3. PMID: 30931771. 10. Choi JS, Ma DS. Changes in oral health indicators due to implementation of the National Health Insurance Services coverage for first molar dental sealant for children and adolescents in South Korea. BMC Oral Health. 2020 Jul 29;20(1):214. doi: 10.1186/s12903-020-01201-8. PMID: 32727427; PMCID: PMC7392644. 11. Arheiam A, Aloshiby A, Gaber A, Fakron S. Dental Fluorosis and Its Associated Factors Amongst Libyan Schoolchildren. Int Dent J. 2022 Aug 3:S0020-6539(22)00080-6. doi: 10.1016/j.identj.2022.04.010. Epub ahead of print. PMID: 35933225. |
| Social isolation  Social support  Also help for people formerly living institutionalized (transition, health care access afterwards) | 1. Olofsson H, Ulander EL, Gustafson Y, Hörnsten C. Association between socioeconomic and health factors and edentulism in people aged 65 and older - a population-based survey. Scand J Public Health. 2018 Nov;46(7):690-698. doi: 10.1177/1403494817717406. Epub 2017 Aug 3. PMID: 28768459. 2. Laniado N, Sanders AE, Fazzari MJ, Badner VM, Singer RH, Finlayson TL, Hua S, Isasi CR. Social support and dental caries experience: Findings from the Hispanic Community Health Study/Study of Latinos Sociocultural Ancillary Study. Community Dent Oral Epidemiol. 2021 Oct;49(5):494-502. doi: 10.1111/cdoe.12626. Epub 2021 Feb 27. PMID: 33638557; PMCID: PMC8390586. 3. Haag DG, Santiago PR, Schuch HS, Brennan DS, Jamieson LM. Is the association between social support and oral health modified by household income? Findings from a national study of adults in Australia. Community Dent Oral Epidemiol. 2022 Jan 6. doi: 10.1111/cdoe.12693. Epub ahead of print. PMID: 34989422. 4. Dahlan R, Ghazal E, Saltaji H, Salami B, Amin M. Impact of social support on oral health among immigrants and ethnic minorities: A systematic review. PLoS One. 2019 Jun 20;14(6):e0218678. doi: 10.1371/journal.pone.0218678. PMID: 31220165; PMCID: PMC6586326. 5. Carrellas A, Day A, Cadet T. Oral Health Care Needs of Young Adults Transitioning from Foster Care. Health Soc Work. 2018 Feb 1;43(1):22-29. doi: 10.1093/hsw/hlx044. PMID: 29190341. |
| Health care provision models (e.g. private / public providers) | 1. Nair R, Luzzi L, Jamieson L, Spencer AJ, Hanna KMB, Do LG. Private Dental Care Benefits Non-Indigenous Children More Than Indigenous Children. JDR Clin Trans Res. 2020 Jul;5(3):244-253. doi: 10.1177/2380084419886869. Epub 2019 Oct 29. PMID: 31661646. |
| Marital status | 1. Murakami K, Ohkubo T, Hashimoto H. Socioeconomic Inequalities in Oral Health Among Unmarried and Married Women: Evidence From a Population-Based Study in Japan. J Epidemiol. 2018 Aug 5;28(8):341-346. doi: 10.2188/jea.JE20170088. Epub 2018 Mar 24. PMID: 29576603; PMCID: PMC6048301. 2. Inoue Y, Zaitsu T, Oshiro A, Ishimaru M, Taira K, Takahashi H, Aida J, Tamiya N. Association of marital status and access to dental care among the Japanese population: a cross-sectional study. BMC Oral Health. 2022 Jul 7;22(1):278. doi: 10.1186/s12903-022-02311-1. PMID: 35799162; PMCID: PMC9264690. |
| Having been to a care provider before (lifetime perspective) | 1. Meisha DE, Alsolami AM, Alharbi GM. Social determinants of seeking emergency and routine dental care in Saudi Arabia during the COVID-19 pandemic. BMC Oral Health. 2021 Apr 26;21(1):212. doi: 10.1186/s12903-021-01577-1. PMID: 33902535; PMCID: PMC8074701. |
| Provider variation (individual variation, workforce type variation) | 1. McKernan SC, Momany ET, Sukalski JMC, Jones MP, Damiano PC, Kuthy RA. Variation in General Dentists' Patterns of Sealant Use in Children with Elevated Caries Risk. JDR Clin Trans Res. 2022 Jun 16:23800844221102836. doi: 10.1177/23800844221102836. Epub ahead of print. PMID: 35708460. 2. Amorim LP, Senna MIB, Paula JS, Rodrigues LG, Chiari APG, Ferreira RC. Oral health work process: disparity between teams in Brazil, 2014. Epidemiol Serv Saude. 2021 Feb 5;30(1):e2019533. English, Portuguese. doi: 10.1590/S1679-49742021000100013. PMID: 33566895. |
| Discrimination (as a barrier to receive preventive care; reasons for discrimination: lack of money, social class and type of working occupation) | 1. Junior OLDA, Menegazzo GR, Fagundes MLB, de Sousa JL, Tôrres LHDN, Giordani JMDA. Perceived discrimination in health services and preventive dental attendance in Brazilian adults. Community Dent Oral Epidemiol. 2020 Dec;48(6):533-539. doi: 10.1111/cdoe.12565. Epub 2020 Jul 23. PMID: 32701203. |
| Equity in covid-19 restrictions (or absence of it) | 1. Hopcraft M, Farmer G. Impact of COVID-19 on the provision of paediatric dental care: Analysis of the Australian Child Dental Benefits Schedule. Community Dent Oral Epidemiol. 2021 Aug;49(4):369-376. doi: 10.1111/cdoe.12611. Epub 2020 Dec 11. PMID: 33314322. |
| Employment (status) | 1. Choi JW, Choi Y, Lee TH, Lee HJ, Ju YJ, Park EC. Employment status and unmet dental care needs in South Korea: a population-based panel study. BMJ Open. 2019 Mar 30;9(3):e022436. doi: 10.1136/bmjopen-2018-022436. PMID: 30928921; PMCID: PMC6475345. |
| Patient-provider relation (barriers) | 1. Chavis SE, Roth E, Dababnah S, Tepper V, Badawi DG, Mullins CD. Qualitative inquiry from multiple perspectives of barriers and facilitators of oral health care for adults with disabilities. Spec Care Dentist. 2022 May 30. doi: 10.1111/scd.12734. Epub ahead of print. PMID: 35636426. |
| Health system barriers for special needs patients (e.g. provider training, logistics, equipment) | 1. Chavis SE, Roth E, Dababnah S, Tepper V, Badawi DG, Mullins CD. Qualitative inquiry from multiple perspectives of barriers and facilitators of oral health care for adults with disabilities. Spec Care Dentist. 2022 May 30. doi: 10.1111/scd.12734. Epub ahead of print. PMID: 35636426. |
| Social inequity due to oral health problems | 1. Baldo Moraes R, Knorst JK, Brondani B, Baldo Marques B, de Sousa Reis M, Henriqson D, Ardenghi TM. Relationship between gingival bleeding and associated factors with reports of verbal bullying in adolescents. J Periodontol. 2021 Feb;92(2):225-233. doi: 10.1002/JPER.19-0745. Epub 2020 Aug 11. PMID: 32716571. |
| Substance use (alcohol, nicotine) | 1. Akinkugbe AA, Garcia DT, Brickhouse TH, Mosavel M. Lifestyle risk factor related disparities in oral cancer examination in the U.S: a population-based cross-sectional study. BMC Public Health. 2020 Jan 31;20(1):153. doi: 10.1186/s12889-020-8247-2. PMID: 32005213; PMCID: PMC6995129. |
| Not receiving health advice due to socioeconomic factors | 1. Ahmadi O, Machuca C, Sabbah W. Socioeconomic inequality in the provision of health advice in dental setting in England, Wales and Northern Ireland. Patient Educ Couns. 2019 Nov;102(11):2068-2072. doi: 10.1016/j.pec.2019.05.012. Epub 2019 May 15. PMID: 31122817. |

# Accessibility

According to “An International Working Definition for Quality of Oral Healthcare” (Righolt et. al, 2019) aims are:

In order to be considered accessible, oral healthcare should

• Be accessible and available, in a timely fashion, to everyone seeking care.

• Be independent of personal characteristics, such as gender, ethnicity, cultural background, socioeconomic status,

religion and belief, geographic location, and/or the patient’s health status.

• Be determined by appointment times and recall intervals that are based on patient needs and preferences.

Number of titles screened: 249

Number of abstracts screened: 243

Number of full texts screened:

| **Possible topics deduced from literature** | **Reference** |
| --- | --- |
| Limitations in supply-side resources and availability of dentists | 1. Zuev MV, Butova VG, Smirnova LE, Kireev VV. Analiz ob''emov stomatologicheskikh profilakticheskikh uslug, predostavliaemykh naseleniiu po programme obiazatel'nogo meditsinskogo strakhovaniia [Analysis of the amount of preventive dental care provided in terms of Compulsory Medical Insurance program]. Stomatologiia (Mosk). 2020;99(1):82-85. Russian. doi: 10.17116/stomat20209901182. PMID: 32125308. 2. Viana IB, Moreira RDS, Martelli PJL, Oliveira ALS, Monteiro IDS. Evaluation of the quality of oral health care in Primary Health Care in Pernambuco, Brazil, 2014. Epidemiol Serv Saude. 2019 Jul 4;28(2):e2018060. English, Portuguese. doi: 10.5123/S1679-49742019000200015. PMID: 31291436. 3. Uguru N, Onwujekwe O, Ogu UU, Uguru C. Access to Oral health care: a focus on dental caries treatment provision in Enugu Nigeria. BMC Oral Health. 2020 May 19;20(1):145. doi: 10.1186/s12903-020-01135-1. PMID: 32429976; PMCID: PMC7236454. 4. Sousa ANA, Shimizu HE. Integrality and comprehensiveness of service provision in Primary Health Care in Brazil (2012-2018). Rev Bras Enferm. 2021 May 28;74(2):e20200500. English, Portuguese. doi: 10.1590/0034-7167-2020-0500. PMID: 34076219. 5. Scalzo MTA, Matta-Machado ATG, Abreu MHNG, Martins RC. Structural characteristics of oral health services in Brazilian Primary Health Care. Braz Oral Res. 2021 Feb 12;35:e023. doi: 10.1590/1807-3107bor-2021.vol35.0023. PMID: 33605354. 6. Satokangas M, Lumme S, Arffman M, Keskimäki I. Trajectory modelling of ambulatory care sensitive conditions in Finland in 1996-2013: assessing the development of equity in primary health care through clustering of geographic areas - an observational retrospective study. BMC Health Serv Res. 2019 Sep 4;19(1):629. doi: 10.1186/s12913-019-4449-7. PMID: 31484530; PMCID: PMC6727548. 7. Ribeiro AGA, Martins RFM, Vissoci JRN, da Silva NC, Rocha TAH, Queiroz RCS, Tonello AS, Staton CA, Facchini LA, Thomaz EBAF. Progress and challenges in potential access to oral health primary care services in Brazil: A population-based panel study with latent transition analysis. PLoS One. 2021 Mar 16;16(3):e0247101. doi: 10.1371/journal.pone.0247101. PMID: 33725008; PMCID: PMC7963056. 8. Pourat N, Chen X, Lee C, Zhou W, Daniel M, Hoang H, Sharma R, Sim H, Sripipatana A, Nair S. HRSA-funded Health Centers Are an Important Source of Care and Reduce Unmet Needs in Primary Care Services. Med Care. 2019 Dec;57(12):996-1001. doi: 10.1097/MLR.0000000000001206. PMID: 31730569. 9. Nguyen V, Daniel M, Joskow R, Lu C, Chen X, Zhou W, Lin S, Sripipatana A, Nair S, Pourat N. Impact of oral health service expansion funding at health centers in the United States. J Public Health Dent. 2020 Sep;80(4):304-312. doi: 10.1111/jphd.12385. Epub 2020 Jul 27. PMID: 32715495. 10. Ghorbani Z, Pakkhesal M, Arshi S, Eghbal MJ, Deghatipour M, Tennant M, Ardakani HM. Challenges impeding integration of oral health into primary health care. East Mediterr Health J. 2018 Mar 5;23(12):802-808. doi: 10.26719/2017.23.12.802. PMID: 29528089. 11. Galvão MHR, Roncalli AG. Desempenho dos municípios brasileiros quanto à oferta de serviços especializados em saúde bucal [Performance of Brazilian municipalities in the supply of specialized oral health services]. Cad Saude Publica. 2021 Jan 11;37(1):e00184119. Portuguese. doi: 10.1590/0102-311X00184119. PMID: 33440412. 12. Folayan MO, Haire BG, Adeniyi AA, Adeyemo WL. Screening Children for Caries: An Ethical Dilemma in Nigeria. New Bioeth. 2018 Jul;24(2):135-149. doi: 10.1080/20502877.2018.1448251. Epub 2018 Mar 13. PMID: 29533155. 13. Chung PC, Chan TC. Association between local spatial accessibility of dental care services and dental care quality. BMC Oral Health. 2021 Nov 17;21(1):582. doi: 10.1186/s12903-021-01943-z. PMID: 34789214; PMCID: PMC8600821. 14. Chou CF, Vallin T, Rogers S, Streeter RA, Worede I, Washko MM, Zangaro GA, Ungard J. Impact of the Health Resources and Services Administration's state oral health workforce grant program on dental workforce density and access to oral health care. Eval Program Plann. 2019 Aug;75:43-53. doi: 10.1016/j.evalprogplan.2019.04.004. Epub 2019 May 3. PMID: 31082654. 15. Cascaes AM, Dotto L, Bomfim RA. Trends in the dental surgeon workforce in Brazil 2007-2014: a time series study using data from the National Registry of Health Services. Epidemiol Serv Saude. 2018 Mar 5;27(1):e201723615. English, Portuguese. doi: 10.5123/S1679-49742018000100015. PMID: 29513857. 16. Bastos LF, Hugo FN, Hilgert JB, Cardozo DD, Bulgarelli AF, Santos CMD. Access to dental services and oral health-related quality of life in the context of primary health care. Braz Oral Res. 2019 Aug 15;33:e018. doi: 10.1590/1807-3107bor-2019.vol33.0018. PMID: 31432923. 17. Baliga SM. Child oral health-care literacy in India: Can access to services be improved? J Indian Soc Pedod Prev Dent. 2019 Jan-Mar;37(1):1-2. doi: 10.4103/JISPPD.JISPPD_18_19. PMID: 30804299. |
| Inequalities based on resident status (e.g. refugees, migrants) as well as language barriers and lack of knowledge among these groups | 1. Zinah E, Al-Ibrahim HM. Oral health problems facing refugees in Europe: a scoping review. BMC Public Health. 2021 Jun 24;21(1):1207. doi: 10.1186/s12889-021-11272-z. PMID: 34162351; PMCID: PMC8223339. 2. Xu K, Watanabe-Galloway S, Qu M, Grimm B, Kim J. Common Diagnoses among Refugee Populations: Linked Results with Statewide Hospital Discharge Database. Ann Glob Health. 2018 Oct 10;84(3):541-550. doi: 10.29024/aogh.2354. PMID: 30835394; PMCID: PMC6748192. 3. Xi S, Song Y, Li X, Li M, Lu Z, Yang Y, Wang Y. Local-Migrant Gaps in Healthcare Utilization Between Older Migrants and Local Residents in China. J Am Geriatr Soc. 2020 Jul;68(7):1560-1567. doi: 10.1111/jgs.16421. Epub 2020 Mar 28. PMID: 32222105. 4. Pullen E, Perry BL, Maupome G. "Does this Look Infected to You?" Social Network Predictors of Dental Help-Seeking Among Mexican Immigrants. J Immigr Minor Health. 2018 Apr;20(2):399-409. doi: 10.1007/s10903-017-0572-x. PMID: 28361383; PMCID: PMC5623174. 5. Pichemin C, Boyer E, Jarno P, Bertaud V, Meuric V, Couatarmanach A. Oral Care Needs Amongst Disadvantaged Migrants in France. Int Dent J. 2022 Aug;72(4):559-564. doi: 10.1016/j.identj.2021.12.002. Epub 2022 Mar 9. PMID: 35279328; PMCID: PMC9381369. 6. Northridge ME, Kumar A, Kaur R. Disparities in Access to Oral Health Care. Annu Rev Public Health. 2020 Apr 2;41:513-535. doi: 10.1146/annurev-publhealth-040119-094318. Epub 2020 Jan 3. PMID: 31900100; PMCID: PMC7125002. 7. Murillo-Pedrozo AM, Martínez-Herrera E, Ronda-Pérez E, Agudelo-Suárez AA. A Qualitative Study of the Health Perceptions in the Venezuelan Immigrant Population in Medellín (Colombia) and Its Conditioning Factors. Int J Environ Res Public Health. 2021 Apr 8;18(8):3897. doi: 10.3390/ijerph18083897. PMID: 33917688; PMCID: PMC8068052. 8. Macdonald ME, Keboa MT, Nurelhuda NM, Lawrence HP, Carnevale F, McNally M, Singhal S, Ka K, Nicolau B. The Oral Health of Refugees and Asylum Seekers in Canada: A Mixed Methods Study Protocol. Int J Environ Res Public Health. 2019 Feb 13;16(4):542. doi: 10.3390/ijerph16040542. PMID: 30781882; PMCID: PMC6406538. 9. Linton JM, Green A; COUNCIL ON COMMUNITY PEDIATRICS. Providing Care for Children in Immigrant Families. Pediatrics. 2019 Sep;144(3):e20192077. doi: 10.1542/peds.2019-2077. Epub 2019 Aug 19. PMID: 31427460. 10. Kohlenberger J, Buber-Ennser I, Rengs B, Leitner S, Landesmann M. Barriers to health care access and service utilization of refugees in Austria: Evidence from a cross-sectional survey. Health Policy. 2019 Sep;123(9):833-839. doi: 10.1016/j.healthpol.2019.01.014. Epub 2019 Mar 6. PMID: 30878171. 11. Klein J, von dem Knesebeck O. Inequalities in health care utilization among migrants and non-migrants in Germany: a systematic review. Int J Equity Health. 2018 Nov 1;17(1):160. doi: 10.1186/s12939-018-0876-z. PMID: 30382861; PMCID: PMC6211605. 12. Keboa MT, Hovey R, Nicolau B, Esfandiari S, Carnevale F, Macdonald ME. Oral healthcare experiences of humanitarian migrants in Montreal, Canada. Can J Public Health. 2019 Aug;110(4):453-461. doi: 10.17269/s41997-019-00193-5. Epub 2019 Mar 8. PMID: 30850954; PMCID: PMC6964583. 13. Kang C, Tomkow L, Farrington R. Access to primary health care for asylum seekers and refugees: a qualitative study of service user experiences in the UK. Br J Gen Pract. 2019 Aug;69(685):e537-e545. doi: 10.3399/bjgp19X701309. Epub 2019 Feb 11. PMID: 30745354; PMCID: PMC6617541. 14. Jervelund SS, Maltesen T, Wimmelmann CL, Petersen JH, Krasnik A. Know where to go: evidence from a controlled trial of a healthcare system information intervention among immigrants. BMC Public Health. 2018 Jul 11;18(1):863. doi: 10.1186/s12889-018-5741-x. PMID: 29996799; PMCID: PMC6042399. 15. Jang SH. Here or There: Recent U.S. Immigrants' Medical and Dental Tourism and Associated Factors. Int J Health Serv. 2018 Jan;48(1):148-165. doi: 10.1177/0020731417732780. Epub 2017 Sep 27. PMID: 28950763. 16. Hunter-Adams J, Cochran J, Laird LD, Paasche-Orlow MK, Geltman PL. Acculturation and a Potential Relationship with Oral Health Outcomes Among Somali Refugees in Massachusetts. J Immigr Minor Health. 2018 Apr;20(2):351-359. doi: 10.1007/s10903-017-0650-0. PMID: 28861739. 17. Gonzales G, Dedania R, Driscoll R. Health Insurance Coverage and Access to Care Among US-Born and Foreign-Born Sexual Minorities. J Immigr Minor Health. 2019 Jun;21(3):540-548. doi: 10.1007/s10903-018-0774-x. PMID: 29946865. 18. Carrasco-Sanz A, Leiva-Gea I, Martin-Alvarez L, Del Torso S, van Esso D, Hadjipanayis A, Kadir A, Ruiz-Canela J, Perez-Gonzalez O, Grossman Z. Migrant children's health problems, care needs, and inequalities: European primary care paediatricians' perspective. Child Care Health Dev. 2018 Mar;44(2):183-187. doi: 10.1111/cch.12538. Epub 2017 Nov 21. PMID: 29159977. |
| Disparities/challenges for patients with special needs, including disabilities, chronic diseases (also elderly), difficulties in accessing care due to disease | 1. Wiener RC, Bhandari R. Association between adverse childhood experiences among children with special healthcare needs and dental care utilization. J Public Health Dent. 2022 Mar;82(2):194-203. doi: 10.1111/jphd.12450. Epub 2021 Mar 9. PMID: 33751582; PMCID: PMC8426408. 2. Tian N, Kobau R, Zack MM, Greenlund KJ. Barriers to and Disparities in Access to Health Care Among Adults Aged ≥18 Years with Epilepsy - United States, 2015 and 2017. MMWR Morb Mortal Wkly Rep. 2022 May 27;71(21):697-702. doi: 10.15585/mmwr.mm7121a1. PMID: 35617131; PMCID: PMC9153461. 3. Thomas C. Dental care in older adults. Br J Community Nurs. 2019 May 2;24(5):233-235. doi: 10.12968/bjcn.2019.24.5.233. PMID: 31059299. 4. Teo CHK, Mahesh MN, Lim GXD. Oral health status and barriers to care in a multiethnic mixed disability center: Rethinking disability community dental services. Spec Care Dentist. 2020 Jul;40(4):344-355. doi: 10.1111/scd.12483. Epub 2020 Jun 5. PMID: 32501578. 5. Sória GS, Nunes BP, Bavaresco CS, Vieira LS, Facchini LA. Acesso e utilização dos serviços de saúde bucal por idosos de Pelotas, Rio Grande do Sul, Brasil [Access to and use of oral health services among the elderly in Pelotas, Rio Grande do Sul State, Brazil]. Cad Saude Publica. 2019 May 2;35(4):e00191718. Portuguese. doi: 10.1590/0102-311X00191718. PMID: 31066780. 6. Schniedewind E, Lindsay RP, Snow S. Comparison of Access to Primary Care Medical and Dental Appointments Between Simulated Patients Who Were Deaf and Patients Who Could Hear. JAMA Netw Open. 2021 Jan 4;4(1):e2032207. doi: 10.1001/jamanetworkopen.2020.32207. PMID: 33475755; PMCID: PMC7821033. 7. Riggs S, Blue C, Golden J. The oral health quality of life for seniors in residential facilities who have direct access to care as compared to those without access. Geriatr Nurs. 2020 Jul-Aug;41(4):406-410. doi: 10.1016/j.gerinurse.2019.12.004. Epub 2020 Mar 18. PMID: 32199738. 8. Prasad R, Edwards J. Removing Barriers to Dental Care for Individuals With Disability. Prim Dent J. 2020 Jun;9(2):62-73. doi: 10.1177/2050168420923872. PMID: 32643569. 9. Parasuraman SR, Anglin TM, McLellan SE, Riley C, Mann MY. Health Care Utilization and Unmet Need Among Youth With Special Health Care Needs. J Adolesc Health. 2018 Oct;63(4):435-444. doi: 10.1016/j.jadohealth.2018.03.020. Epub 2018 Aug 2. PMID: 30078509. 10. Obeidat R, Noureldin A, Bitouni A, Abdellatif H, Lewis-Miranda S, Liu S, Badner V, Timothé P. Oral health needs of U.S. children with developmental disorders: a population-based study. BMC Public Health. 2022 Apr 29;22(1):861. doi: 10.1186/s12889-022-13237-2. PMID: 35488224; PMCID: PMC9055681. 11. Mielnik-Błaszczak M, Skawińska-Bednarczyk A, Michałowski A, Błaszczak J. Problems with access to dental treatment for children with epilepsy. Epilepsy Behav. 2018 Apr;81:107-110. doi: 10.1016/j.yebeh.2017.12.014. Epub 2018 Feb 12. PMID: 29449139. 12. Meyerhoefer CD, Zuvekas SH, Farkhad BF, Moeller JF, Manski RJ. The demand for preventive and restorative dental services among older adults. Health Econ. 2019 Sep;28(9):1151-1158. doi: 10.1002/hec.3921. Epub 2019 Jul 2. PMID: 31264323; PMCID: PMC6706303. 13. Martin MA, Collazo GR, Frese WA, Glassgow AE. Oral Health Problems and Solutions in High-Risk Children and Young Adults. J Dent Child (Chic). 2018 Sep 15;85(3):125-132. PMID: 30869589. 14. Marchini L, Reynolds JC, Caplan DJ, Sasser S, Russell C. Predictors of having a dentist among older adults in Iowa. Community Dent Oral Epidemiol. 2020 Jun;48(3):240-247. doi: 10.1111/cdoe.12521. Epub 2020 Feb 10. PMID: 32043281. 15. Mansoor D, Al Halabi M, Khamis AH, Kowash M. Oral health challenges facing Dubai children with Autism Spectrum Disorder at home and in accessing oral health care. Eur J Paediatr Dent. 2018 Jun;19(2):127-133. doi: 10.23804/ejpd.2018.19.02.06. PMID: 29790776. 16. Machado Luz FW, Silva AER, Perroni AP, Goettems ML, Boscato N. Impact of Seniors Centers on oral health-related quality of life of older adults. Rev Saude Publica. 2020 Jan 20;54:07. doi: 10.11606/s1518-8787.2020054001648. PMID: 31967276; PMCID: PMC6961966. 17. Lim MAWT, Liberali SAC, Calache H, Parashos P, Borromeo GL. Mentoring of oral health professionals is crucial to improving access to care for people with special needs. PLoS One. 2022 Apr 25;17(4):e0266879. doi: 10.1371/journal.pone.0266879. PMID: 35468149; PMCID: PMC9037927. 18. Lim MAWT, Liberali SAC, Borromeo GL. Utilisation of dental services for people with special health care needs in Australia. BMC Oral Health. 2020 Dec 11;20(1):360. doi: 10.1186/s12903-020-01354-6. PMID: 33308211; PMCID: PMC7733299. 19. Kim YJ, Lim YW, Paik HR, Lee JY, Kim SY. What influences use of dental services by the Korean disabled people? The role of perceived barriers in dental care system. Community Dent Health. 2019 May 30;36(2):101-105. doi: 10.1922/CDH_4456Young05. PMID: 31070873. 20. Kim G, Carrico C, Ivey C, Wunsch PB. Impact of sensory adapted dental environment on children with developmental disabilities. Spec Care Dentist. 2019 Mar;39(2):180-187. doi: 10.1111/scd.12360. Epub 2019 Feb 6. PMID: 30729554. 21. Ishimaru M, Ono S, Morita K, Matsui H, Yasunaga H. Domiciliary dental care among homebound older adults: A nested case-control study in Japan. Geriatr Gerontol Int. 2019 Jul;19(7):679-683. doi: 10.1111/ggi.13676. Epub 2019 Apr 29. PMID: 31037823. 22. Gondlach C, Catteau C, Hennequin M, Faulks D. Evaluation of a Care Coordination Initiative in Improving Access to Dental Care for Persons with Disability. Int J Environ Res Public Health. 2019 Aug 1;16(15):2753. doi: 10.3390/ijerph16152753. PMID: 31374964; PMCID: PMC6696062. 23. Fenning RM, Steinberg-Epstein R, Butter EM, Chan J, McKinnon-Bermingham K, Hammersmith KJ, Moffitt J, Shui AM, Parker RA, Coury DL, Wang PP, Kuhlthau KA. Access to Dental Visits and Correlates of Preventive Dental Care in Children with Autism Spectrum Disorder. J Autism Dev Disord. 2020 Oct;50(10):3739-3747. doi: 10.1007/s10803-020-04420-8. PMID: 32112232. 24. Estrada I, Kunzel C, Schrimshaw EW, Greenblatt AP, Metcalf SS, Northridge ME. "Seniors only want respect": designing an oral health program for older adults. Spec Care Dentist. 2018 Jan;38(1):3-12. doi: 10.1111/scd.12265. Epub 2018 Jan 4. PMID: 29314188; PMCID: PMC5785560. 25. Croze J, Le S M, Feugueur G, Blanchard J, Azoulay T. Difficultés de prise en charge de la santé bucco-dentaire des personnes en situation de handicap [Difficulties in dental health care for the disabled]. Sante Publique. 2018 November-December;30(6):821-827. French. doi: 10.3917/spub.187.0821. PMID: 30990270. 26. Chadwick D, Chapman M, Davies G. Factors affecting access to daily oral and dental care among adults with intellectual disabilities. J Appl Res Intellect Disabil. 2018 May;31(3):379-394. doi: 10.1111/jar.12415. Epub 2017 Oct 17. PMID: 29044754. 27. Byrappagari D, Jung Y, Chen K. Oral health care for patients with developmental disabilities: A survey of Michigan general dentists. Spec Care Dentist. 2018 Sep;38(5):281-290. doi: 10.1111/scd.12303. Epub 2018 Jun 26. PMID: 29944189. 28. Blaizot A, Catteau C, Delfosse C, Hamel O, Trentesaux T. Obstacles to comprehensive dental care in patients with sustained limitations of their decision-making abilities: findings from a Delphi study. Eur J Oral Sci. 2018 Jun;126(3):222-233. doi: 10.1111/eos.12413. Epub 2018 Apr 20. PMID: 29676806. 29. Berdahl T, Biener A, McCormick MC, Guevara JP, Simpson L. Annual Report on Children's Healthcare: Healthcare Access and Utilization by Obesity Status in the United States. Acad Pediatr. 2020 Mar;20(2):175-187. doi: 10.1016/j.acap.2019.11.020. Epub 2019 Dec 13. PMID: 31843708. 30. Bas AC, Azogui-Lévy S. L’effet du non-recours aux soins médicaux et dentaires sur les dépenses de santé des personnes ayant une maladie chronique [Lack of medical or dental care: Impact on health expenditures for people with chronic disease]. Rev Epidemiol Sante Publique. 2020 Apr;68(2):91-98. French. doi: 10.1016/j.respe.2019.12.002. Epub 2020 Feb 20. PMID: 32089349. 31. Baliga SM. Child oral health-care literacy in India: Can access to services be improved? J Indian Soc Pedod Prev Dent. 2019 Jan-Mar;37(1):1-2. doi: 10.4103/JISPPD.JISPPD_18_19. PMID: 30804299. 32. Baccaglini L, Kusi Appiah A, Ray M, Yu F. US adults with diabetes mellitus: Variability in oral healthcare utilization. PLoS One. 2021 May 5;16(5):e0251120. doi: 10.1371/journal.pone.0251120. PMID: 33951111; PMCID: PMC8099075. 33. Aquilanti L, Santarelli A, Mascitti M, Procaccini M, Rappelli G. Dental Care Access and the Elderly: What Is the Role of Teledentistry? A Systematic Review. Int J Environ Res Public Health. 2020 Dec 4;17(23):9053. doi: 10.3390/ijerph17239053. PMID: 33291719; PMCID: PMC7729836. 34. Alshihri AA, Al-Askar MH, Aldossary MS. Barriers to Professional Dental Care among Children with Autism Spectrum Disorder. J Autism Dev Disord. 2021 Aug;51(8):2988-2994. doi: 10.1007/s10803-020-04759-y. Epub 2020 Oct 21. Erratum in: J Autism Dev Disord. 2020 Nov 27;: PMID: 33089446. |
| Integrated oral health care (e.g. integration between primary and oral care, between oral care and long term care faciliites), care coordination | 1. West JF, King RK. Academic and Community Partnerships: Increasing Access Through Collaborative Care. J Dent Educ. 2019 Feb;83(2 Suppl):S23-S27. doi: 10.21815/JDE.019.039. PMID: 30709936. 2. Sousa ANA, Shimizu HE. Integrality and comprehensiveness of service provision in Primary Health Care in Brazil (2012-2018). Rev Bras Enferm. 2021 May 28;74(2):e20200500. English, Portuguese. doi: 10.1590/0034-7167-2020-0500. PMID: 34076219. 3. Shrivastava R, Couturier Y, Kadoch N, Girard F, Bedos C, Macdonald ME, Torrie J, Emami E. Patients' perspectives on integrated oral healthcare in a northern Quebec Indigenous primary health care organisation: a qualitative study. BMJ Open. 2019 Jul 30;9(7):e030005. doi: 10.1136/bmjopen-2019-030005. PMID: 31366663; PMCID: PMC6677955. 4. Robinson FG, Fischbach H, Salisbury J, Stefanik D, Kearney R, Fields HW. Clinical Integration in a Dental School Clinic Through an Enhanced Patient Intake Process. J Dent Educ. 2019 Sep;83(9):1030-1038. doi: 10.21815/JDE.019.105. Epub 2019 May 13. PMID: 31085688. 5. Riggs S, Blue C, Golden J. The oral health quality of life for seniors in residential facilities who have direct access to care as compared to those without access. Geriatr Nurs. 2020 Jul-Aug;41(4):406-410. doi: 10.1016/j.gerinurse.2019.12.004. Epub 2020 Mar 18. PMID: 32199738. 6. Pourat N, Martinez AE, Haley LA, Crall JJ. Colocation Does Not Equal Integration: Identifying and Measuring Best Practices in Primary Care Integration of Children's Oral Health Services in Health Centers. J Evid Based Dent Pract. 2020 Dec;20(4):101469. doi: 10.1016/j.jebdp.2020.101469. Epub 2020 Jul 14. PMID: 33303098. 7. Northridge ME, Kumar A, Kaur R. Disparities in Access to Oral Health Care. Annu Rev Public Health. 2020 Apr 2;41:513-535. doi: 10.1146/annurev-publhealth-040119-094318. Epub 2020 Jan 3. PMID: 31900100; PMCID: PMC7125002. 8. Nguyen VH, Lin SC, Cappelli DP, Nair S. The association between dental, general, and mental health status among underserved and vulnerable populations served at health centers in the US. J Public Health Dent. 2018 Dec;78(1):41-48. doi: 10.1111/jphd.12234. Epub 2017 Jul 18. PMID: 28719064. 9. Moss ME, Grodner A, Dasanayake AP, Beasley CM. County-level correlates of dental service utilization for low income pregnant women. Ecologic study of the North Carolina Medicaid for Pregnant Women (MPW) program. BMC Health Serv Res. 2021 Jan 13;21(1):61. doi: 10.1186/s12913-021-06060-9. PMID: 33435967; PMCID: PMC7805091. 10. Lutfiyya MN, Gross AJ, Soffe B, Lipsky MS. Dental care utilization: examining the associations between health services deficits and not having a dental visit in past 12 months. BMC Public Health. 2019 Mar 5;19(1):265. doi: 10.1186/s12889-019-6590-y. PMID: 30836954; PMCID: PMC6402128. 11. Linabarger M, Brown M, Patel N. A Pilot Study of Integration of Medical and Dental Care in 6 States. Prev Chronic Dis. 2021 Jul 22;18:E72. doi: 10.5888/pcd18.210027. PMID: 34296994; PMCID: PMC8300541. 12. Kranz AM, Rozier RG, Stein BD, Dick AW. Do Oral Health Services in Medical Offices Replace Pediatric Dental Visits? J Dent Res. 2020 Jul;99(8):891-897. doi: 10.1177/0022034520916161. Epub 2020 Apr 23. PMID: 32325007; PMCID: PMC7346745. 13. Jackson DN, Passmore S, Fryer CS, Chen J, Kleinman DV, Horowitz AM, Butler J 3rd, Garza MA, Quinn SC, Thomas SB. Mission of Mercy emergency dental clinics: an opportunity to promote general and oral health. BMC Public Health. 2018 Jul 13;18(1):878. doi: 10.1186/s12889-018-5792-z. PMID: 30005660; PMCID: PMC6045818. 14. Gondlach C, Catteau C, Hennequin M, Faulks D. Evaluation of a Care Coordination Initiative in Improving Access to Dental Care for Persons with Disability. Int J Environ Res Public Health. 2019 Aug 1;16(15):2753. doi: 10.3390/ijerph16152753. PMID: 31374964; PMCID: PMC6696062. 15. Cunha CRHD, Harzheim E, Medeiros OL, D'Avila OP, Martins C, Wollmann L, Faller LA. Primary Health Care Portfolio: Assuring of integrality in the Family Health and Oral Health Teams in Brazil. Cien Saude Colet. 2020 Mar;25(4):1313-1326. Portuguese, English. doi: 10.1590/1413-81232020254.31862019. Epub 2019 Dec 19. PMID: 32267434. 16. Baccaglini L, Kusi Appiah A, Ray M, Yu F. US adults with diabetes mellitus: Variability in oral healthcare utilization. PLoS One. 2021 May 5;16(5):e0251120. doi: 10.1371/journal.pone.0251120. PMID: 33951111; PMCID: PMC8099075. |
| Affordability of care and out of pocket spending, population (or insurance) coverage to improve financial access | 1. Wehby GL, Lyu W, Shane DM. The Impact of the ACA Medicaid Expansions on Dental Visits by Dental Coverage Generosity and Dentist Supply. Med Care. 2019 Oct;57(10):781-787. doi: 10.1097/MLR.0000000000001181. Erratum in: Med Care. 2019 Dec;57(12):1008. PMID: 31433313; PMCID: PMC6742569. 2. Uguru N, Onwujekwe O, Ogu UU, Uguru C. Access to Oral health care: a focus on dental caries treatment provision in Enugu Nigeria. BMC Oral Health. 2020 May 19;20(1):145. doi: 10.1186/s12903-020-01135-1. PMID: 32429976; PMCID: PMC7236454. 3. Tynan A, Walker D, Tucker T, Fisher B, Fisher T. Factors influencing the perceived importance of oral health within a rural Aboriginal and Torres Strait Islander community in Australia. BMC Public Health. 2020 Apr 17;20(1):514. doi: 10.1186/s12889-020-08673-x. PMID: 32303214; PMCID: PMC7164228. 4. Tiwari T, Rai N. Oral Health of African Immigrants: A participatory approach to needs assessment. Community Dent Health. 2021 Feb 25;38(1):48-52. doi: 10.1922/CDH_00087Tiwari05. PMID: 33507651. 5. Taylor H, Holmes AM, Blackburn J. Prevalence of and factors associated with unmet dental need among the US adult population in 2016. Community Dent Oral Epidemiol. 2021 Aug;49(4):346-353. doi: 10.1111/cdoe.12607. Epub 2020 Dec 4. PMID: 33274505. 6. Sousa ANA, Shimizu HE. Integrality and comprehensiveness of service provision in Primary Health Care in Brazil (2012-2018). Rev Bras Enferm. 2021 May 28;74(2):e20200500. English, Portuguese. doi: 10.1590/0034-7167-2020-0500. PMID: 34076219. 7. Song J, Kim JN, Tomar S, Wong LN. The Impact of the Affordable Care Act on Dental Care: An Integrative Literature Review. Int J Environ Res Public Health. 2021 Jul 25;18(15):7865. doi: 10.3390/ijerph18157865. PMID: 34360160; PMCID: PMC8345350. 8. Shin BM, Jung SH, Kim MH, Ryu JI. Did the extended coverage policy contribute to alleviating socioeconomic inequality in untreated dental caries of both children and adolescents in South Korea? BMC Oral Health. 2020 Apr 22;20(1):124. doi: 10.1186/s12903-020-01112-8. PMID: 32321493; PMCID: PMC7178997. 9. Serban N, Anderson A, Oberst G, Edupuganti N, Ramachandran R, Solipuram SR, Lu T. Assessment of Dentist Participation in Public Insurance Programs for Children in the US. JAMA Netw Open. 2022 Jul 1;5(7):e2221444. doi: 10.1001/jamanetworkopen.2022.21444. PMID: 35816300; PMCID: PMC9274318. 10. Semprini J, Wehby G. Impact of Medicaid dental coverage expansion on self-reported tooth loss in low-income adults. J Am Dent Assoc. 2022 Sep;153(9):839-847. doi: 10.1016/j.adaj.2022.03.003. Epub 2022 May 3. PMID: 35513904; PMCID: PMC9427673. 11. Sanchez P, Salamonson Y, Everett B, George A. Barriers and Predictors Associated With Accessing Oral Healthcare Among Patients With Cardiovascular Disease in Australia. J Cardiovasc Nurs. 2019 May/Jun;34(3):208-214. doi: 10.1097/JCN.0000000000000559. PMID: 30589656. 12. Rossi TRA, Lorena Sobrinho JE, Chaves SCL, Martelli PJL. Economic crisis, austerity and its effects on the financing of oral health and access to public and private services. Cien Saude Colet. 2019 Dec;24(12):4427-4436. Portuguese, English. doi: 10.1590/1413-812320182412.25582019. Epub 2019 Aug 30. PMID: 31778493. 13. Rosenberg J, Shabanova V, McCollum S, Sharifi M. Insurance and Health Care Outcomes in Regions Where Undocumented Children Are Medicaid-Eligible. Pediatrics. 2022 Sep 1;150(3):e2022057034. doi: 10.1542/peds.2022-057034. PMID: 36004541. 14. Reynolds JC, Sukalski JMC, McKernan SC, Damiano PC. Member and dentist narratives about a dental programme for the Medicaid expansion population: A content analysis. Community Dent Oral Epidemiol. 2019 Dec;47(6):485-493. doi: 10.1111/cdoe.12490. Epub 2019 Aug 22. PMID: 31441086. 15. Reid J, Koopu P, Burkhardt N, Stewart T, Anderson A, Harwood M. Oral and dental health and health care for Māori with type 2 diabetes: A qualitative study. Community Dent Oral Epidemiol. 2020 Apr;48(2):101-108. doi: 10.1111/cdoe.12501. Epub 2019 Oct 27. PMID: 31657040. 16. Probst LF, Pucca Junior GA, Pereira AC, Carli AD. Impact of financial crises on oral health indicators: an integrative review of the literature. Cien Saude Colet. 2019 Dec;24(12):4437-4448. Portuguese, English. doi: 10.1590/1413-812320182412.23132019. Epub 2019 Aug 8. PMID: 31778494. 17. Pourat N, Chen X, Lee C, Zhou W, Daniel M, Hoang H, Sharma R, Sim H, Sripipatana A, Nair S. HRSA-funded Health Centers Are an Important Source of Care and Reduce Unmet Needs in Primary Care Services. Med Care. 2019 Dec;57(12):996-1001. doi: 10.1097/MLR.0000000000001206. PMID: 31730569. 18. Piedrasanta FC, Boyd LD, Vineyard J, LaSpina L. Oral Health Knowledge, Acculturation and Utilization of Oral Health Services among a Hispanic and Latino Population. J Dent Hyg. 2021 Oct;95(5):22-31. PMID: 34654712. 19. Peck J, Sedgley CM, Schwarz E, Replogle KJ. The impact of the Affordable Care Act on provision of endodontic services within a dental school setting in Oregon. J Public Health Dent. 2019 Mar;79(2):175-180. doi: 10.1111/jphd.12314. Epub 2019 Apr 24. PMID: 31020668. 20. Palm W, Webb E, Hernández-Quevedo C, Scarpetti G, Lessof S, Siciliani L, van Ginneken E. Gaps in coverage and access in the European Union. Health Policy. 2021 Mar;125(3):341-350. doi: 10.1016/j.healthpol.2020.12.011. Epub 2020 Dec 25. PMID: 33431257. 21. Northridge ME, Kumar A, Kaur R. Disparities in Access to Oral Health Care. Annu Rev Public Health. 2020 Apr 2;41:513-535. doi: 10.1146/annurev-publhealth-040119-094318. Epub 2020 Jan 3. PMID: 31900100; PMCID: PMC7125002. 22. Nieto V, Arnett M, Furgeson D. Attitudes and Access Patterns of Michigan Veterans Ineligible for Oral Healthcare Benefits: A cross-sectional study. J Dent Hyg. 2019 Aug;93(4):6-13. PMID: 31409719. 23. Nguyen VH, Lin SC, Cappelli DP, Nair S. The association between dental, general, and mental health status among underserved and vulnerable populations served at health centers in the US. J Public Health Dent. 2018 Dec;78(1):41-48. doi: 10.1111/jphd.12234. Epub 2017 Jul 18. PMID: 28719064. 24. Negro KS, Scott JM, Marcenko M, Chi DL. Assessing the Feasibility of Oral Health Interventions Delivered by Social Workers to Children and Families in the Foster Care System. Pediatr Dent. 2019 Jan 15;41(1):48-51. PMID: 30803477; PMCID: PMC6392446. 25. Naavaal S, Griffin SO, Jones JA. Impact of Making Dental Care Affordable on Quality of Life in Adults Aged 45 Years and Older. J Aging Health. 2020 Aug-Sep;32(7-8):861-870. doi: 10.1177/0898264319857967. Epub 2019 Jul 1. PMID: 31258028; PMCID: PMC7978487. 26. Moran V, Suhrcke M, Ruiz-Castell M, Barré J, Huiart L. Investigating unmet need for healthcare using the European Health Interview Survey: a cross-sectional survey study of Luxembourg. BMJ Open. 2021 Aug 3;11(8):e048860. doi: 10.1136/bmjopen-2021-048860. PMID: 34344682; PMCID: PMC8336210. 27. Meyerhoefer CD, Zuvekas SH, Farkhad BF, Moeller JF, Manski RJ. The demand for preventive and restorative dental services among older adults. Health Econ. 2019 Sep;28(9):1151-1158. doi: 10.1002/hec.3921. Epub 2019 Jul 2. PMID: 31264323; PMCID: PMC6706303. 28. Mariño R, Glenister K, Bourke L, Morgan M, Atala-Acevedo C, Simmons D. Patterns of use of oral health care services in Australian rural adults: the Crossroads-II Dental sub-study. Aust Dent J. 2021 Dec;66(4):397-405. doi: 10.1111/adj.12865. Epub 2021 Jul 10. PMID: 34152019. 29. Marchini L, Reynolds JC, Caplan DJ, Sasser S, Russell C. Predictors of having a dentist among older adults in Iowa. Community Dent Oral Epidemiol. 2020 Jun;48(3):240-247. doi: 10.1111/cdoe.12521. Epub 2020 Feb 10. PMID: 32043281. 30. Lyu W, Shane DM, Wehby GL. Effects of the Recent Medicaid Expansions on Dental Preventive Services and Treatments. Med Care. 2020 Aug;58(8):749-755. doi: 10.1097/MLR.0000000000001344. PMID: 32692142; PMCID: PMC8211012. 31. Listl S, Quiñonez C, Vujicic M. Including oral diseases and conditions in universal health coverage. Bull World Health Organ. 2021 Jun 1;99(6):407. doi: 10.2471/BLT.21.285530. PMID: 34108747; PMCID: PMC8164177. 32. Lipton BJ, Finlayson TL, Decker SL, Manski RJ, Yang M. The Association Between Medicaid Adult Dental Coverage And Children's Oral Health. Health Aff (Millwood). 2021 Nov;40(11):1731-1739. doi: 10.1377/hlthaff.2021.01135. PMID: 34724426; PMCID: PMC8609949. 33. Kieffer EC, Goold SD, Buchmueller T, Nalliah R, Beathard E, Kirch MA, Solway E, Tipirneni R, Clark SJ, Haggins AN, Patel MR, Ayanian JZ. Beneficiaries' perspectives on improved oral health and its mediators after Medicaid expansion in Michigan: a mixed methods study. J Public Health Dent. 2022 Jan;82(1):11-21. doi: 10.1111/jphd.12447. Epub 2021 Mar 22. PMID: 33754344. 34. Johansen ME, Richardson CR. The Ecology of Medical Care Before and After the Affordable Care Act: Trends From 2002 to 2016. Ann Fam Med. 2019 Nov;17(6):526-537. doi: 10.1370/afm.2462. PMID: 31712291; PMCID: PMC6846273. 35. Jatrana S, Crampton P. Do financial barriers to access to primary health care increase the risk of poor health? Longitudinal evidence from New Zealand. Soc Sci Med. 2021 Nov;288:113255. doi: 10.1016/j.socscimed.2020.113255. Epub 2020 Jul 28. PMID: 32819742. 36. Jang YE, Kim CB, Kim NH. Influence of dental insurance coverage on access to preventive periodontal care in middle-aged and elderly populations: analysis of representative Korean Community Health Survey Data (2011-2015). Int Dent J. 2019 Dec;69(6):445-453. doi: 10.1111/idj.12488. Epub 2019 May 22. PMID: 31115042; PMCID: PMC9379082. 37. Idon PI, Sotunde OA, Ogundare TO, Yusuf J, Makanjuola JO, Mohammed A, Igweagu CE, Alalade O. Access to root canal treatment in a Nigerian sub-population: assessment of the effect of dental health insurance. Afr Health Sci. 2021 Mar;21(1):470-477. doi: 10.4314/ahs.v21i1.57. PMID: 34394329; PMCID: PMC8356604. 38. Hollederer A, Wildner M. Ungedeckter medizinischer Versorgungsbedarf in Deutschland: Ergebnisse im EU-SILC-Survey 2005 bis 2014 [Unmet Medical Need in Germany: Analyses of EU-SILC-Survey from 2005 to 2014]. Dtsch Med Wochenschr. 2019 Jan;144(1):e1-e11. German. doi: 10.1055/a-0671-4605. Epub 2019 Jan 2. PMID: 30602179. 39. Hill CM, Jones MP, Chi DL. Effects of Adult Medicaid Dental Benefits Elimination on Child Dental Care Use. Med Care. 2022 Aug 1;60(8):579-587. doi: 10.1097/MLR.0000000000001739. Epub 2022 May 26. PMID: 35616495. 40. Gupta A, Feldman S, Perkins RB, Stokes A, Sankar V, Villa A. Predictors of dental care use, unmet dental care need, and barriers to unmet need among women: results from NHANES, 2011 to 2016. J Public Health Dent. 2019 Dec;79(4):324-333. doi: 10.1111/jphd.12334. Epub 2019 Aug 13. PMID: 31407356. 41. Gray L, McNeill L, Yi W, Zvonereva A, Brunton P, Mei L. The "business" of dentistry: Consumers' (patients') criteria in the selection and evaluation of dental services. PLoS One. 2021 Aug 6;16(8):e0253517. doi: 10.1371/journal.pone.0253517. PMID: 34358252; PMCID: PMC8345823. 42. Gnanamanickam ES, Teusner DN, Arrow PG, Brennan DS. Dental insurance, service use and health outcomes in Australia: a systematic review. Aust Dent J. 2018 Mar;63(1):4-13. doi: 10.1111/adj.12534. Epub 2017 Sep 11. PMID: 28543627. 43. França MASA, Freire MDCM, Pereira EM, Marcelo VC. Indicators proposed by the Brazilian Ministry of Health for monitoring and evaluating oral health actions in the National Health System: documentary research, 2000-2017. Epidemiol Serv Saude. 2020 Feb 17;29(1):e2018406. English, Portuguese. doi: 10.5123/S1679-49742020000100002. PMID: 32074194. 44. Finlayson TL, Chuang E, Baek JD, Seidman R. Dental Service Utilization Among Children in the Child Welfare System. Matern Child Health J. 2018 May;22(5):753-761. doi: 10.1007/s10995-018-2444-y. PMID: 29423585; PMCID: PMC5895518. 45. Finlayson TL, Asgari P, Dougherty E, Tadese BK, Stamm N, Nunez-Alvarez A. Child, caregiver, and family factors associated with child dental utilization among Mexican migrant families in California. Community Dent Health. 2018 May 30;35(2):89-94. doi: 10.1922/CDH_4201Finlayson06. PMID: 29645407. 46. Farietta TP, Lu B, Tumin R. Ohio's Medicaid Expansion and Unmet Health Needs Among Low-Income Women of Reproductive Age. Matern Child Health J. 2018 Dec;22(12):1771-1779. doi: 10.1007/s10995-018-2575-1. PMID: 30006730. 47. Elani HW, Kawachi I, Sommers BD. Medicaid healthy behavior incentives and use of dental services. Health Serv Res. 2021 Aug;56(4):702-708. doi: 10.1111/1475-6773.13672. Epub 2021 May 18. PMID: 34008193; PMCID: PMC8313965. 48. Dalstrom M, Weinzimmer LG, Foulger R, Klein CJ. Medicaid expansion and accessibility to healthcare: The Illinois experience. Public Health Nurs. 2021 Sep;38(5):720-729. doi: 10.1111/phn.12899. Epub 2021 Mar 29. PMID: 33778982. 49. Che X, Sohn M, Park HJ. Unmet dental care needs in South Korea: how do they differ by insurance system? J Health Serv Res Policy. 2019 Jul;24(3):164-171. doi: 10.1177/1355819619835260. PMID: 31291763. 50. Chaiyachati KH, Hom JK, Wong C, Nasseh K, Chen X, Beggin A, Zygmunt E, Vujicic M, Grande D. Access to primary and dental care among adults newly enrolled in Medicaid. Am J Manag Care. 2019 Mar;25(3):135-139. PMID: 30875182. 51. Burgette JM, Weyant RJ, Ettinger AK, Miller E, Ray KN. What is the association between income loss during the COVID-19 pandemic and children's dental care? J Am Dent Assoc. 2021 May;152(5):369-376. doi: 10.1016/j.adaj.2021.02.001. Epub 2021 Feb 6. PMID: 33810832; PMCID: PMC7867385. 52. Bas AC, Dourgnon P, Azogui-Levy S, Wittwer J. Impact of fees on access to dental care: evidence from France. Eur J Public Health. 2020 Dec 11;30(6):1066-1071. doi: 10.1093/eurpub/ckaa143. PMID: 32789438. 53. Baliga SM. Child oral health-care literacy in India: Can access to services be improved? J Indian Soc Pedod Prev Dent. 2019 Jan-Mar;37(1):1-2. doi: 10.4103/JISPPD.JISPPD_18_19. PMID: 30804299. 54. Baicker K, Allen HL, Wright BJ, Taubman SL, Finkelstein AN. The Effect of Medicaid on Dental Care of Poor Adults: Evidence from the Oregon Health Insurance Experiment. Health Serv Res. 2018 Aug;53(4):2147-2164. doi: 10.1111/1475-6773.12757. Epub 2017 Sep 8. PMID: 28884818; PMCID: PMC6052006. |
| Socioeconomic status and resulting disparities | 1. Watt RG, Mathur MR, Aida J, Bönecker M, Venturelli R, Gansky SA. Oral Health Disparities in Children: A Canary in the Coalmine? Pediatr Clin North Am. 2018 Oct;65(5):965-979. doi: 10.1016/j.pcl.2018.05.006. PMID: 30213357. 2. Viana IB, Moreira RDS, Martelli PJL, Oliveira ALS, Monteiro IDS. Evaluation of the quality of oral health care in Primary Health Care in Pernambuco, Brazil, 2014. Epidemiol Serv Saude. 2019 Jul 4;28(2):e2018060. English, Portuguese. doi: 10.5123/S1679-49742019000200015. PMID: 31291436. 3. Verheire F, De Visschere L, Fernandez C, Lambert M, Marks L. Accessibility to oral health care for people on social assistance: a survey of social service providers from Public Welfare Centers in Flanders. Int Dent J. 2018 Dec;68(6):393-404. doi: 10.1111/idj.12395. Epub 2018 May 9. PMID: 29744868; PMCID: PMC9379086. 4. Uguru N, Onwujekwe O, Ogu UU, Uguru C. Access to Oral health care: a focus on dental caries treatment provision in Enugu Nigeria. BMC Oral Health. 2020 May 19;20(1):145. doi: 10.1186/s12903-020-01135-1. PMID: 32429976; PMCID: PMC7236454. 5. Tynan A, Walker D, Tucker T, Fisher B, Fisher T. Factors influencing the perceived importance of oral health within a rural Aboriginal and Torres Strait Islander community in Australia. BMC Public Health. 2020 Apr 17;20(1):514. doi: 10.1186/s12889-020-08673-x. PMID: 32303214; PMCID: PMC7164228. 6. Trudnak Fowler T, Matthews G, Black C, Crosby Kowal H, Vodicka P, Edgerton E. Evaluation of a Comprehensive Oral Health Services Program in School-Based Health Centers. Matern Child Health J. 2018 Jul;22(7):998-1007. doi: 10.1007/s10995-018-2478-1. PMID: 29453520. 7. Torppa-Saarinen E, Tolvanen M, Lahti S, Suominen AL. Changes and determinants of unmet oral health treatment need. Community Dent Oral Epidemiol. 2021 Apr;49(2):158-165. doi: 10.1111/cdoe.12587. Epub 2020 Oct 26. PMID: 33104256. 8. Tian N, Kobau R, Zack MM, Greenlund KJ. Barriers to and Disparities in Access to Health Care Among Adults Aged ≥18 Years with Epilepsy - United States, 2015 and 2017. MMWR Morb Mortal Wkly Rep. 2022 May 27;71(21):697-702. doi: 10.15585/mmwr.mm7121a1. PMID: 35617131; PMCID: PMC9153461. 9. Testa A, Ganson KT, Jackson DB, Bojorquez-Ramirez P, Weiser SD, Nagata JM. Food insecurity and oral health care experiences during pregnancy: Findings from the Pregnancy Risk Assessment Monitoring System. J Am Dent Assoc. 2022 Jun;153(6):503-510. doi: 10.1016/j.adaj.2021.12.010. Epub 2022 Mar 15. PMID: 35303979. 10. Taylor H, Holmes AM, Blackburn J. Prevalence of and factors associated with unmet dental need among the US adult population in 2016. Community Dent Oral Epidemiol. 2021 Aug;49(4):346-353. doi: 10.1111/cdoe.12607. Epub 2020 Dec 4. PMID: 33274505. 11. Stormon N, Pradhan A, McAuliffe A, Ford PJ. Does a facilitated pathway improve access to dental services for homeless and disadvantaged adults? Eval Program Plann. 2018 Dec;71:46-50. doi: 10.1016/j.evalprogplan.2018.08.002. Epub 2018 Aug 8. PMID: 30114615. 12. Silva CCD, Savian CM, Prevedello BP, Zamberlan C, Dalpian DM, Santos BZD. Access and use of dental services by pregnant women: an integrative literature review. Cien Saude Colet. 2020 Mar;25(3):827-835. Portuguese, English. doi: 10.1590/1413-81232020253.01192018. Epub 2018 Jul 7. PMID: 32159653. 13. Shin BM, Jung SH, Kim MH, Ryu JI. Did the extended coverage policy contribute to alleviating socioeconomic inequality in untreated dental caries of both children and adolescents in South Korea? BMC Oral Health. 2020 Apr 22;20(1):124. doi: 10.1186/s12903-020-01112-8. PMID: 32321493; PMCID: PMC7178997. 14. Shahar S, Lau H, Puteh SEW, Amara S, Razak NA. Health, access and nutritional issues among low-income population in Malaysia: introductory note. BMC Public Health. 2019 Jun 13;19(Suppl 4):552. doi: 10.1186/s12889-019-6852-8. PMID: 31196021; PMCID: PMC6565540. 15. Semprini J, Wehby G. Impact of Medicaid dental coverage expansion on self-reported tooth loss in low-income adults. J Am Dent Assoc. 2022 Sep;153(9):839-847. doi: 10.1016/j.adaj.2022.03.003. Epub 2022 May 3. PMID: 35513904; PMCID: PMC9427673. 16. Rungreangkulkij S, Silarat M, Kotnara I. Prisoners' perceptions of the healthcare service: A qualitative study. Nurs Health Sci. 2021 Jun;23(2):304-311. doi: 10.1111/nhs.12813. Epub 2021 Feb 13. PMID: 33438827. 17. Ruas BM, Castilho LS, Carneiro NCR, Cardoso NMM, Reis AB, Silva MESE, Borges-Oliveira AC. Integrality of care for hemodialysis patient in Brazil: an analysis of access to dental care. Cien Saude Colet. 2020 Feb;25(2):533-540. doi: 10.1590/1413-81232020252.06362018. Epub 2018 Jun 20. PMID: 32022193. 18. Rosing K, Leggett H, Csikar J, Vinall-Collier K, Christensen LB, Whelton H, Douglas GVA. Barriers and facilitators for prevention in Danish dental care. Acta Odontol Scand. 2019 Aug;77(6):439-451. doi: 10.1080/00016357.2019.1587503. Epub 2019 Mar 25. PMID: 30905244. 19. Reich SM, Ochoa W, Gaona A, Salcedo Y, Espino Bardales G, Newhart V, Lin J, Díaz G. Disparities in Caregivers' Experiences at the Dentist With Their Young Child. Acad Pediatr. 2019 Nov-Dec;19(8):969-977. doi: 10.1016/j.acap.2019.03.006. Epub 2019 Mar 21. PMID: 30904582; PMCID: PMC6828576. 20. Reda SF, Reda SM, Thomson WM, Schwendicke F. Inequality in Utilization of Dental Services: A Systematic Review and Meta-analysis. Am J Public Health. 2018 Feb;108(2):e1-e7. doi: 10.2105/AJPH.2017.304180. Epub 2017 Dec 21. PMID: 29267052; PMCID: PMC5846590. 21. Probst LF, Pucca Junior GA, Pereira AC, Carli AD. Impact of financial crises on oral health indicators: an integrative review of the literature. Cien Saude Colet. 2019 Dec;24(12):4437-4448. Portuguese, English. doi: 10.1590/1413-812320182412.23132019. Epub 2019 Aug 8. PMID: 31778494. 22. Pledger M, Dunn P, McDonald J, Cumming J, Saville-Smith K. Is the use of health services by older adults in New Zealand associated with their housing tenure? N Z Med J. 2020 Feb 21;133(1510):23-34. PMID: 32078598. 23. Palaia G, Semprini F, De-Giorgio F, Ralli M, Bossù M, Arcangeli A, Ercoli L, Polimeni A. Evaluation of dental demands and needs of people experiencing homelessness in the city of Rome, Italy. Eur Rev Med Pharmacol Sci. 2022 Aug;26(15):5460-5465. doi: 10.26355/eurrev_202208_29414. PMID: 35993641. 24. Northridge ME, Kumar A, Kaur R. Disparities in Access to Oral Health Care. Annu Rev Public Health. 2020 Apr 2;41:513-535. doi: 10.1146/annurev-publhealth-040119-094318. Epub 2020 Jan 3. PMID: 31900100; PMCID: PMC7125002. 25. Neves M, Giordani JMDA, Hugo FN. Atenção primária à saúde bucal no Brasil: processo de trabalho das equipes de saúde bucal [Primary dental healthcare in Brazil: the work process of oral health teams]. Cien Saude Colet. 2019 May 30;24(5):1809-1820. Portuguese. doi: 10.1590/1413-81232018245.08892017. PMID: 31166514. 26. Listl S, Quiñonez C, Vujicic M. Including oral diseases and conditions in universal health coverage. Bull World Health Organ. 2021 Jun 1;99(6):407. doi: 10.2471/BLT.21.285530. PMID: 34108747; PMCID: PMC8164177. 27. Leonhardt KA, Hirsch C. Sozioökonomische Gradienten bei der Inanspruchnahme zahnärztlicher Leistungen in Deutschland [Socioeconomic Gradients in Dental Care Accessibility in Germany]. Gesundheitswesen. 2019 Apr;81(4):347-350. German. doi: 10.1055/s-0042-120268. Epub 2017 Mar 17. PMID: 28315288. 28. Kyoon-Achan G, Schroth RJ, DeMaré D, Sturym M, Edwards JM, Sanguins J, Campbell R, Chartrand F, Bertone M, Moffatt MEK. First Nations and Metis peoples' access and equity challenges with early childhood oral health: a qualitative study. Int J Equity Health. 2021 Jun 7;20(1):134. doi: 10.1186/s12939-021-01476-5. PMID: 34098968; PMCID: PMC8183050. 29. Hernández-Vásquez A, Bendezu-Quispe G, Azañedo D, Santero M. Use of oral health care services in Peru: trends of socio-economic inequalities before and after the implementation of Universal Health Assurance. BMC Oral Health. 2019 Mar 7;19(1):39. doi: 10.1186/s12903-019-0731-7. PMID: 30845948; PMCID: PMC6407179. 30. Hachey S, Clovis J, Lamarche K. Children's Oral Health and Barriers to Seeking Care: Perspectives of Caregivers Seeking Pediatric Hospital Dental Treatment. Healthc Policy. 2019 Aug;15(1):29-39. doi: 10.12927/hcpol.2019.25940. PMID: 31629454; PMCID: PMC7008694. 31. Goode J, Hoang H, Crocombe L. Strategies to improve access to and uptake of dental care by people experiencing homelessness in Australia: a grey literature review. Aust Health Rev. 2020 Apr;44(2):297-303. doi: 10.1071/AH18187. PMID: 31262395. 32. Glenister KM, Bourke L, Bolitho L, Wright S, Roberts S, Kemp W, Rhode L, Bhat R, Tremper S, Magliano DJ, Morgan M, Mariño R, Adam W, Simmons D. Longitudinal study of health, disease and access to care in rural Victoria: the Crossroads-II study: methods. BMC Public Health. 2018 May 30;18(1):670. doi: 10.1186/s12889-018-5511-9. PMID: 29843659; PMCID: PMC5975688. 33. Gao X, Ding M, Xu M, Wu H, Zhang C, Wang X, Feng X, Tai B, Hu D, Lin H, Wang B, Zheng S, Liu X, Rong W, Wang W, Wang C, Si Y. Utilization of dental services and associated factors among preschool children in China. BMC Oral Health. 2020 Jan 8;20(1):9. doi: 10.1186/s12903-019-0996-x. PMID: 31914978; PMCID: PMC6950915. 34. Fleming E, Frantsve-Hawley J, Minter-Jordan M. Health Equity Needs Teeth. AMA J Ethics. 2022 Jan 1;24(1):E48-56. doi: 10.1001/amajethics.2022.48. PMID: 35133728. 35. Filipponi T, Richards W, Coll AM. Oral health knowledge, perceptions and practices among parents, guardians and teachers in South Wales, UK: A qualitative study. Br Dent J. 2018 Apr 13;224(7):517-522. doi: 10.1038/sj.bdj.2018.223. Epub 2018 Mar 23. PMID: 29576611. 36. Echeverria MS, Silva AER, Agostini BA, Schuch HS, Demarco FF. Regular use of dental services among university students in southern Brazil. Rev Saude Publica. 2020;54:85. doi: 10.11606/s1518-8787.2020054001935. Epub 2020 Aug 21. PMID: 32844983; PMCID: PMC7438030. 37. Csikar J, Vinall-Collier K, Richemond JM, Talbot J, Serban ST, Douglas GVA. Identifying the barriers and facilitators for homeless people to achieve good oral health. Community Dent Health. 2019 May 30;36(2):137-142. doi: 10.1922/CDH_4488Csikar06. PMID: 31070874. 38. Che X, Sohn M, Moon S, Park HJ. Unmet dental care needs of homeless adults in South Korea: Findings from a national survey. Health Soc Care Community. 2022 Jul;30(4):e1134-e1142. doi: 10.1111/hsc.13521. Epub 2021 Jul 21. PMID: 34291516. 39. Carreiro DL, Souza JGS, Coutinho WLM, Haikal DS, Martins AMEBL. Acesso aos serviços odontológicos e fatores associados: estudo populacional domiciliar [Access to dental services and related factors: a home-based population study]. Cien Saude Colet. 2019 Mar;24(3):1021-1032. Portuguese. doi: 10.1590/1413-81232018243.04272017. PMID: 30892522. 40. Bauman JM, Souza JGS, Bauman CD, Flório FM. Epidemiological pattern of malocclusion in Brazilian preschoolers. Cien Saude Colet. 2018 Nov;23(11):3861-3868. Portuguese, English. doi: 10.1590/1413-812320182311.24722016. PMID: 30427456. 41. Bastos TF, Medina LPB, Sousa NFDS, Lima MG, Malta DC, Barros MBA. Income inequalities in oral health and access to dental services in the Brazilian population: National Health Survey, 2013. Rev Bras Epidemiol. 2019 Oct 7;22Suppl 02(Suppl 02):E190015.SUPL.2. doi: 10.1590/1980-549720190015.supl.2. PMID: 31596386. 42. Barasuol JC, Garcia LP, Freitas RC, Dalpian DM, Menezes JVNB, Santos BZ. Dental care utilization among children in Brazil: an exploratory study based on data from national household surveys. Cien Saude Colet. 2019 Feb;24(2):649-657. doi: 10.1590/1413-81232018242.03232017. PMID: 30726397. 43. Baliga SM. Child oral health-care literacy in India: Can access to services be improved? J Indian Soc Pedod Prev Dent. 2019 Jan-Mar;37(1):1-2. doi: 10.4103/JISPPD.JISPPD_18_19. PMID: 30804299. |
| Disparities based on geographical location, distance to facilities, transportation problems (geographical access) | 1. Uguru N, Onwujekwe O, Ogu UU, Uguru C. Access to Oral health care: a focus on dental caries treatment provision in Enugu Nigeria. BMC Oral Health. 2020 May 19;20(1):145. doi: 10.1186/s12903-020-01135-1. PMID: 32429976; PMCID: PMC7236454. 2. Tynan A, Deeth L, McKenzie D. An integrated oral health program for rural residential aged care facilities: a mixed methods comparative study. BMC Health Serv Res. 2018 Jul 3;18(1):515. doi: 10.1186/s12913-018-3321-5. PMID: 29970073; PMCID: PMC6029389. 3. Trudnak Fowler T, Matthews G, Black C, Crosby Kowal H, Vodicka P, Edgerton E. Evaluation of a Comprehensive Oral Health Services Program in School-Based Health Centers. Matern Child Health J. 2018 Jul;22(7):998-1007. doi: 10.1007/s10995-018-2478-1. PMID: 29453520. 4. Tiwari T, Rai N. Oral Health of African Immigrants: A participatory approach to needs assessment. Community Dent Health. 2021 Feb 25;38(1):48-52. doi: 10.1922/CDH_00087Tiwari05. PMID: 33507651. 5. Simon L, Ahern J, Fox K, Barrow J, Palmer N. Variation in dental services by rurality among privately insured adults in the United States. J Public Health Dent. 2021 Dec;81(1):50-56. doi: 10.1111/jphd.12398. Epub 2020 Sep 12. PMID: 32918758. 6. Shin H, Cho HA. Spatial barriers and the bypassing of nearby dental clinics for dental services: a secondary data analysis in Korea. BMJ Open. 2019 Jan 25;9(1):e024116. doi: 10.1136/bmjopen-2018-024116. PMID: 30782727; PMCID: PMC6347908. 7. Serban N, Anderson A, Oberst G, Edupuganti N, Ramachandran R, Solipuram SR, Lu T. Assessment of Dentist Participation in Public Insurance Programs for Children in the US. JAMA Netw Open. 2022 Jul 1;5(7):e2221444. doi: 10.1001/jamanetworkopen.2022.21444. PMID: 35816300; PMCID: PMC9274318. 8. Scalzo MTA, Matta-Machado ATG, Abreu MHNG, Martins RC. Structural characteristics of oral health services in Brazilian Primary Health Care. Braz Oral Res. 2021 Feb 12;35:e023. doi: 10.1590/1807-3107bor-2021.vol35.0023. PMID: 33605354. 9. Satokangas M, Lumme S, Arffman M, Keskimäki I. Trajectory modelling of ambulatory care sensitive conditions in Finland in 1996-2013: assessing the development of equity in primary health care through clustering of geographic areas - an observational retrospective study. BMC Health Serv Res. 2019 Sep 4;19(1):629. doi: 10.1186/s12913-019-4449-7. PMID: 31484530; PMCID: PMC6727548. 10. Pourat N, Lu C, Chen X, Zhou W, Hair B, Bolton J, Sripipatana A. Trends in access to care among rural patients served at HRSA-funded health centers. J Rural Health. 2022 Sep;38(4):970-979. doi: 10.1111/jrh.12626. Epub 2021 Oct 7. PMID: 34617337. 11. Nascimento AC, Moysés ST, Werneck RI, Gabardo MCL, Moysés SJ. Assessment of public oral healthcare services in Curitiba, Brazil: a cross-sectional study using the Primary Care Assessment Tool (PCATool). BMJ Open. 2019 Jan 17;9(1):e023283. doi: 10.1136/bmjopen-2018-023283. PMID: 30659038; PMCID: PMC6340483. 12. Moss ME, Grodner A, Dasanayake AP, Beasley CM. County-level correlates of dental service utilization for low income pregnant women. Ecologic study of the North Carolina Medicaid for Pregnant Women (MPW) program. BMC Health Serv Res. 2021 Jan 13;21(1):61. doi: 10.1186/s12913-021-06060-9. PMID: 33435967; PMCID: PMC7805091. 13. Moran V, Suhrcke M, Ruiz-Castell M, Barré J, Huiart L. Investigating unmet need for healthcare using the European Health Interview Survey: a cross-sectional survey study of Luxembourg. BMJ Open. 2021 Aug 3;11(8):e048860. doi: 10.1136/bmjopen-2021-048860. PMID: 34344682; PMCID: PMC8336210. 14. Mielnik-Błaszczak M, Skawińska-Bednarczyk A, Michałowski A, Błaszczak J. Problems with access to dental treatment for children with epilepsy. Epilepsy Behav. 2018 Apr;81:107-110. doi: 10.1016/j.yebeh.2017.12.014. Epub 2018 Feb 12. PMID: 29449139. 15. McKernan SC, Reynolds JC, Ingleshwar A, Pooley M, Kuthy RA, Damiano PC. Transportation Barriers and Use of Dental Services among Medicaid-Insured Adults. JDR Clin Trans Res. 2018 Jan;3(1):101-108. doi: 10.1177/2380084417714795. Epub 2017 Jun 9. PMID: 30938652. 16. Lopez N, Sager J, Gonzaga A. Dental and Dental Therapy Students' Perspectives on How to Build Interest in and Commitment to Rural Dentistry. J Dent Educ. 2019 Aug;83(8):946-952. doi: 10.21815/JDE.019.094. Epub 2019 May 13. PMID: 31085687. 17. Larson JE, Leider JP, Knudson A. A Practice Report From the Northern Dental Access Center: Offering Wraparound Services at a Community Dental Access Clinic to Improve Treatment Outcomes Among Rural Populations in Poverty. J Public Health Manag Pract. 2019 Sep/Oct;25(5):E22-E29. doi: 10.1097/PHH.0000000000000767. PMID: 31348173. 18. Kyoon-Achan G, Schroth RJ, DeMaré D, Sturym M, Edwards JM, Sanguins J, Campbell R, Chartrand F, Bertone M, Moffatt MEK. First Nations and Metis peoples' access and equity challenges with early childhood oral health: a qualitative study. Int J Equity Health. 2021 Jun 7;20(1):134. doi: 10.1186/s12939-021-01476-5. PMID: 34098968; PMCID: PMC8183050. 19. Kiuchi S, Aida J, Kusama T, Yamamoto T, Hoshi M, Yamamoto T, Kondo K, Osaka K. Does public transportation reduce inequalities in access to dental care among older adults? Japan Gerontological Evaluation Study. Community Dent Oral Epidemiol. 2020 Apr;48(2):109-118. doi: 10.1111/cdoe.12508. Epub 2019 Nov 15. PMID: 31729067. 20. Glenister KM, Bourke L, Bolitho L, Wright S, Roberts S, Kemp W, Rhode L, Bhat R, Tremper S, Magliano DJ, Morgan M, Mariño R, Adam W, Simmons D. Longitudinal study of health, disease and access to care in rural Victoria: the Crossroads-II study: methods. BMC Public Health. 2018 May 30;18(1):670. doi: 10.1186/s12889-018-5511-9. PMID: 29843659; PMCID: PMC5975688. 21. Choi SE, Kalenderian E, Normand SL. Measuring the quality of dental care among privately insured children in the United States. Health Serv Res. 2022 Feb;57(1):137-144. doi: 10.1111/1475-6773.13713. Epub 2021 Jul 29. PMID: 34327703; PMCID: PMC8763286. 22. Baliga SM. Child oral health-care literacy in India: Can access to services be improved? J Indian Soc Pedod Prev Dent. 2019 Jan-Mar;37(1):1-2. doi: 10.4103/JISPPD.JISPPD_18_19. PMID: 30804299. 23. Alhozgi A, Feine JS, Tanwir F, Shrivastava R, Galarneau C, Emami E. Rural-urban disparities in patient satisfaction with oral health care: a provincial survey. BMC Oral Health. 2021 May 15;21(1):261. doi: 10.1186/s12903-021-01613-0. PMID: 33992110; PMCID: PMC8122552. |
| Disparities based on ethnicities/cultural background and lack of awareness/knowledge among certain populations | 1. Tynan A, Walker D, Tucker T, Fisher B, Fisher T. Factors influencing the perceived importance of oral health within a rural Aboriginal and Torres Strait Islander community in Australia. BMC Public Health. 2020 Apr 17;20(1):514. doi: 10.1186/s12889-020-08673-x. PMID: 32303214; PMCID: PMC7164228. 2. Tiwari T, Rai N. Oral Health of African Immigrants: A participatory approach to needs assessment. Community Dent Health. 2021 Feb 25;38(1):48-52. doi: 10.1922/CDH_00087Tiwari05. PMID: 33507651. 3. Taylor H, Holmes AM, Blackburn J. Prevalence of and factors associated with unmet dental need among the US adult population in 2016. Community Dent Oral Epidemiol. 2021 Aug;49(4):346-353. doi: 10.1111/cdoe.12607. Epub 2020 Dec 4. PMID: 33274505. 4. Taiwo OO, Panas R. Roles of community pharmacists in improving oral health awareness in Plateau State, Northern Nigeria. Int Dent J. 2018 Aug;68(4):287-294. English. doi: 10.1111/idj.12383. Epub 2018 Feb 15. PMID: 29446064; PMCID: PMC9378883. 5. Silva CCD, Savian CM, Prevedello BP, Zamberlan C, Dalpian DM, Santos BZD. Access and use of dental services by pregnant women: an integrative literature review. Cien Saude Colet. 2020 Mar;25(3):827-835. Portuguese, English. doi: 10.1590/1413-81232020253.01192018. Epub 2018 Jul 7. PMID: 32159653. 6. Sanchez P, Salamonson Y, Everett B, George A. Barriers and Predictors Associated With Accessing Oral Healthcare Among Patients With Cardiovascular Disease in Australia. J Cardiovasc Nurs. 2019 May/Jun;34(3):208-214. doi: 10.1097/JCN.0000000000000559. PMID: 30589656. 7. Ruas BM, Castilho LS, Carneiro NCR, Cardoso NMM, Reis AB, Silva MESE, Borges-Oliveira AC. Integrality of care for hemodialysis patient in Brazil: an analysis of access to dental care. Cien Saude Colet. 2020 Feb;25(2):533-540. doi: 10.1590/1413-81232020252.06362018. Epub 2018 Jun 20. PMID: 32022193. 8. Rosing K, Leggett H, Csikar J, Vinall-Collier K, Christensen LB, Whelton H, Douglas GVA. Barriers and facilitators for prevention in Danish dental care. Acta Odontol Scand. 2019 Aug;77(6):439-451. doi: 10.1080/00016357.2019.1587503. Epub 2019 Mar 25. PMID: 30905244. 9. Reynolds JC, Sukalski JMC, McKernan SC, Damiano PC. Member and dentist narratives about a dental programme for the Medicaid expansion population: A content analysis. Community Dent Oral Epidemiol. 2019 Dec;47(6):485-493. doi: 10.1111/cdoe.12490. Epub 2019 Aug 22. PMID: 31441086. 10. Reid J, Koopu P, Burkhardt N, Stewart T, Anderson A, Harwood M. Oral and dental health and health care for Māori with type 2 diabetes: A qualitative study. Community Dent Oral Epidemiol. 2020 Apr;48(2):101-108. doi: 10.1111/cdoe.12501. Epub 2019 Oct 27. PMID: 31657040. 11. Reich SM, Ochoa W, Gaona A, Salcedo Y, Espino Bardales G, Newhart V, Lin J, Díaz G. Disparities in Caregivers' Experiences at the Dentist With Their Young Child. Acad Pediatr. 2019 Nov-Dec;19(8):969-977. doi: 10.1016/j.acap.2019.03.006. Epub 2019 Mar 21. PMID: 30904582; PMCID: PMC6828576. 12. Reda SF, Reda SM, Thomson WM, Schwendicke F. Inequality in Utilization of Dental Services: A Systematic Review and Meta-analysis. Am J Public Health. 2018 Feb;108(2):e1-e7. doi: 10.2105/AJPH.2017.304180. Epub 2017 Dec 21. PMID: 29267052; PMCID: PMC5846590. 13. Pourat N, Martinez AE, Haley LA, Crall JJ. Colocation Does Not Equal Integration: Identifying and Measuring Best Practices in Primary Care Integration of Children's Oral Health Services in Health Centers. J Evid Based Dent Pract. 2020 Dec;20(4):101469. doi: 10.1016/j.jebdp.2020.101469. Epub 2020 Jul 14. PMID: 33303098. 14. Piedrasanta FC, Boyd LD, Vineyard J, LaSpina L. Oral Health Knowledge, Acculturation and Utilization of Oral Health Services among a Hispanic and Latino Population. J Dent Hyg. 2021 Oct;95(5):22-31. PMID: 34654712. 15. Northridge ME, Kumar A, Kaur R. Disparities in Access to Oral Health Care. Annu Rev Public Health. 2020 Apr 2;41:513-535. doi: 10.1146/annurev-publhealth-040119-094318. Epub 2020 Jan 3. PMID: 31900100; PMCID: PMC7125002. 16. Nascimento AC, Moysés ST, Werneck RI, Gabardo MCL, Moysés SJ. Assessment of public oral healthcare services in Curitiba, Brazil: a cross-sectional study using the Primary Care Assessment Tool (PCATool). BMJ Open. 2019 Jan 17;9(1):e023283. doi: 10.1136/bmjopen-2018-023283. PMID: 30659038; PMCID: PMC6340483. 17. Naavaal S, Carrico CK, Williams TL, Brickhouse TH, Raskin SE. Oral Health Knowledge, Barriers to Dental Care, and Awareness of a Medicaid Pregnancy Dental Coverage Among Reproductive-Age Women. J Womens Health (Larchmt). 2022 Mar;31(3):401-407. doi: 10.1089/jwh.2021.0252. Epub 2021 Oct 19. PMID: 34665671. 18. Muthra S, Hamilton R, Leopold K, Dodson E, Mooney D, Wallington SF, Dash C, Adams-Campbell LL. A qualitative study of oral health knowledge among African Americans. PLoS One. 2019 Jul 10;14(7):e0219426. doi: 10.1371/journal.pone.0219426. PMID: 31291338; PMCID: PMC6619789. 19. Kyoon-Achan G, Schroth RJ, DeMaré D, Sturym M, Edwards JM, Sanguins J, Campbell R, Chartrand F, Bertone M, Moffatt MEK. First Nations and Metis peoples' access and equity challenges with early childhood oral health: a qualitative study. Int J Equity Health. 2021 Jun 7;20(1):134. doi: 10.1186/s12939-021-01476-5. PMID: 34098968; PMCID: PMC8183050. 20. Kum SS, Northridge ME, Metcalf SS. Using focus groups to design systems science models that promote oral health equity. BMC Oral Health. 2018 Jun 4;18(1):99. doi: 10.1186/s12903-018-0560-0. PMID: 29866084; PMCID: PMC5987593. 21. Gray L, McNeill L, Yi W, Zvonereva A, Brunton P, Mei L. The "business" of dentistry: Consumers' (patients') criteria in the selection and evaluation of dental services. PLoS One. 2021 Aug 6;16(8):e0253517. doi: 10.1371/journal.pone.0253517. PMID: 34358252; PMCID: PMC8345823. 22. Gao X, Ding M, Xu M, Wu H, Zhang C, Wang X, Feng X, Tai B, Hu D, Lin H, Wang B, Zheng S, Liu X, Rong W, Wang W, Wang C, Si Y. Utilization of dental services and associated factors among preschool children in China. BMC Oral Health. 2020 Jan 8;20(1):9. doi: 10.1186/s12903-019-0996-x. PMID: 31914978; PMCID: PMC6950915. 23. Fleming E, Frantsve-Hawley J, Minter-Jordan M. Health Equity Needs Teeth. AMA J Ethics. 2022 Jan 1;24(1):E48-56. doi: 10.1001/amajethics.2022.48. PMID: 35133728. 24. Davis J, Liu M, Kao D, Gu X, Cherry-Peppers G. Using GIS to Analyze Inequality in Access to Dental Care in the District of Columbia. AMA J Ethics. 2022 Jan 1;24(1):E41-47. doi: 10.1001/amajethics.2022.41. PMID: 35133727; PMCID: PMC9210465. 25. Como DH, Stein Duker LI, Polido JC, Cermak SA. The Persistence of Oral Health Disparities for African American Children: A Scoping Review. Int J Environ Res Public Health. 2019 Feb 27;16(5):710. doi: 10.3390/ijerph16050710. PMID: 30818846; PMCID: PMC6427601. 26. Comassetto MO, Baumgarten A, Kindlein KA, Hilgert JB, Figueiredo MC, Faustino-Silva DD. Access to oral health in early childhood in the city of Porto Alegre, Brazil. Cien Saude Colet. 2019 Mar;24(3):953-961. Portuguese, English. doi: 10.1590/1413-81232018243.29082016. PMID: 30892516. 27. Carreiro DL, Souza JGS, Coutinho WLM, Haikal DS, Martins AMEBL. Acesso aos serviços odontológicos e fatores associados: estudo populacional domiciliar [Access to dental services and related factors: a home-based population study]. Cien Saude Colet. 2019 Mar;24(3):1021-1032. Portuguese. doi: 10.1590/1413-81232018243.04272017. PMID: 30892522. 28. Baliga SM. Child oral health-care literacy in India: Can access to services be improved? J Indian Soc Pedod Prev Dent. 2019 Jan-Mar;37(1):1-2. doi: 10.4103/JISPPD.JISPPD_18_19. PMID: 30804299. 29. Bahramian H, Mohebbi SZ, Khami MR, Quinonez RB. Qualitative exploration of barriers and facilitators of dental service utilization of pregnant women: A triangulation approach. BMC Pregnancy Childbirth. 2018 May 10;18(1):153. doi: 10.1186/s12884-018-1773-6. PMID: 29747592; PMCID: PMC5946421. |
| Discrimination (LGBTQ+, racism) | 1. Tharp G, Wohlford M, Shukla A. Reviewing challenges in access to oral health services among the LGBTQ+ community in Indiana and Michigan: A cross-sectional, exploratory study. PLoS One. 2022 Feb 25;17(2):e0264271. doi: 10.1371/journal.pone.0264271. PMID: 35213637; PMCID: PMC8880834. 2. Samuel SR, Muragaboopathy V, Patil S. Transgender HIV status, self-perceived dental care barriers, and residents' stigma, willingness to treat them in a community dental outreach program: Cross-sectional study. Spec Care Dentist. 2018 Sep;38(5):307-312. doi: 10.1111/scd.12315. Epub 2018 Jul 18. PMID: 30019539. 3. Monopoli M, Johnson IB. Undermining racism: A road to promoting equity in oral health. J Public Health Dent. 2022 Mar;82 Suppl 1(Suppl 1):123-127. doi: 10.1111/jphd.12511. PMID: 35726464; PMCID: PMC9545495. 4. Demopoulos CA, Kohli R, Dhar S, Raju K. Racial and oral health equity in dental school curricula. J Public Health Dent. 2022 Mar;82 Suppl 1(Suppl 1):114-122. doi: 10.1111/jphd.12516. PMID: 35726460; PMCID: PMC9544994. 5. Borrell LN, Williams DR. Racism and oral health equity in the United States: Identifying its effects and providing future directions. J Public Health Dent. 2022 Mar;82 Suppl 1(Suppl 1):8-11. doi: 10.1111/jphd.12501. Epub 2022 Jan 27. PMID: 35088413; PMCID: PMC9541355. |
| Fear | 1. Stormon N, Pradhan A, McAuliffe A, Ford PJ. Does a facilitated pathway improve access to dental services for homeless and disadvantaged adults? Eval Program Plann. 2018 Dec;71:46-50. doi: 10.1016/j.evalprogplan.2018.08.002. Epub 2018 Aug 8. PMID: 30114615. 2. Mariño R, Glenister K, Bourke L, Morgan M, Atala-Acevedo C, Simmons D. Patterns of use of oral health care services in Australian rural adults: the Crossroads-II Dental sub-study. Aust Dent J. 2021 Dec;66(4):397-405. doi: 10.1111/adj.12865. Epub 2021 Jul 10. PMID: 34152019. 3. Bahramian H, Mohebbi SZ, Khami MR, Quinonez RB. Qualitative exploration of barriers and facilitators of dental service utilization of pregnant women: A triangulation approach. BMC Pregnancy Childbirth. 2018 May 10;18(1):153. doi: 10.1186/s12884-018-1773-6. PMID: 29747592; PMCID: PMC5946421. |
| Ability to have paid leave vs. unpaid leave | 1. Stoddard-Dare P, DeRigne L, Mallett C, Quinn L. How does paid sick leave relate to health care affordability and poverty among US workers? Soc Work Health Care. 2018 May-Jun;57(5):376-392. doi: 10.1080/00981389.2018.1447532. Epub 2018 Mar 12. PMID: 29528789. |
| Big data and technological innovations as part of oral health care transformation | 1. Shetty V, Yamamoto J, Yale K. Re-architecting oral healthcare for the 21st century. J Dent. 2018 Jul;74 Suppl 1(Suppl 1):S10-S14. doi: 10.1016/j.jdent.2018.04.017. PMID: 29929582; PMCID: PMC6020157. |
| Perceptions of need for oral health care | 1. Robinson PG, Douglas GVA, Gibson BJ, Godson J, Vinall-Collier K, Pavitt S, Hulme C. Remuneration of primary dental care in England: a qualitative framework analysis of perspectives of a new service delivery model incorporating incentives for improved access, quality and health outcomes. BMJ Open. 2019 Oct 3;9(10):e031886. doi: 10.1136/bmjopen-2019-031886. PMID: 31585977; PMCID: PMC6797242. |
| Suboptimal patient experiences and satisfaction with dentists, trust | 1. Reich SM, Ochoa W, Gaona A, Salcedo Y, Espino Bardales G, Newhart V, Lin J, Díaz G. Disparities in Caregivers' Experiences at the Dentist With Their Young Child. Acad Pediatr. 2019 Nov-Dec;19(8):969-977. doi: 10.1016/j.acap.2019.03.006. Epub 2019 Mar 21. PMID: 30904582; PMCID: PMC6828576. 2. Guivarc'h M, Saliba-Serre B, Le Coz P, Bukiet F. A cross-sectional analysis of patient care pathways and profiles in a dental emergency department. Int Dent J. 2020 Feb;70(1):21-28. doi: 10.1111/idj.12516. Epub 2019 Aug 29. PMID: 31468531; PMCID: PMC9379167. |
| Network ties of citizens, social participation | 1. Pullen E, Perry BL, Maupome G. "Does this Look Infected to You?" Social Network Predictors of Dental Help-Seeking Among Mexican Immigrants. J Immigr Minor Health. 2018 Apr;20(2):399-409. doi: 10.1007/s10903-017-0572-x. PMID: 28361383; PMCID: PMC5623174. 2. Kim JR, Jeong B, Park KS, Kang YS. Associations of Generalized Trust and Social Participation at the Individual Level with Unmet Healthcare Needs in Communities with High Mortality. J Korean Med Sci. 2018 Mar 12;33(11):e84. doi: 10.3346/jkms.2018.33.e84. PMID: 29495134; PMCID: PMC5835583. |
| Remote oral health care, teledentistry, domiciliary care | 1. Pourat N, Chen X, Spetz J. The relationship between portable school-based oral health prevention services and subsequent oral health treatment. J Public Health Dent. 2020 Sep;80(4):278-287. doi: 10.1111/jphd.12382. Epub 2020 Aug 20. PMID: 32815555. 2. McFarland KK, Nayar P, Chandak A, Gupta N. Formative evaluation of a teledentistry training programme for oral health professionals. Eur J Dent Educ. 2018 May;22(2):109-114. doi: 10.1111/eje.12265. Epub 2017 Mar 15. PMID: 28294493. 3. Kintzle S, Rivas WA, Castro CA. Satisfaction of the Use of Telehealth and Access to Care for Veterans During the COVID-19 Pandemic. Telemed J E Health. 2022 May;28(5):706-711. doi: 10.1089/tmj.2021.0262. Epub 2021 Sep 22. PMID: 34551276. 4. Ishimaru M, Ono S, Morita K, Matsui H, Yasunaga H. Domiciliary dental care among homebound older adults: A nested case-control study in Japan. Geriatr Gerontol Int. 2019 Jul;19(7):679-683. doi: 10.1111/ggi.13676. Epub 2019 Apr 29. PMID: 31037823. 5. Aquilanti L, Santarelli A, Mascitti M, Procaccini M, Rappelli G. Dental Care Access and the Elderly: What Is the Role of Teledentistry? A Systematic Review. Int J Environ Res Public Health. 2020 Dec 4;17(23):9053. doi: 10.3390/ijerph17239053. PMID: 33291719; PMCID: PMC7729836. |
| Unmet need among patients with mental illness | 1. Nguyen VH, Lin SC, Cappelli DP, Nair S. The association between dental, general, and mental health status among underserved and vulnerable populations served at health centers in the US. J Public Health Dent. 2018 Dec;78(1):41-48. doi: 10.1111/jphd.12234. Epub 2017 Jul 18. PMID: 28719064. 2. Lam PC, John DA, Galfalvy H, Kunzel C, Lewis-Fernández R. Oral Health-Related Quality of Life Among Publicly Insured Mental Health Service Outpatients With Serious Mental Illness. Psychiatr Serv. 2019 Dec 1;70(12):1101-1109. doi: 10.1176/appi.ps.201900111. Epub 2019 Sep 16. PMID: 31522632. 3. Choi KH, Shin S, Lee E, Lee SW. Access to Dental Care and Depressive Illness: Results from the Korea National Health Nutrition Examination Survey. Medicina (Kaunas). 2020 Apr 19;56(4):189. doi: 10.3390/medicina56040189. PMID: 32325902; PMCID: PMC7230332. 4. Aljabri MK, Gadibalban IZ, Kalboush AM, Sadek HS, Abed HH. Barriers to special care patients with mental illness receiving oral healthcare. A cross sectional study in the Holy City of Makkah, Saudi Arabia. Saudi Med J. 2018 Apr;39(4):419-423. doi: 10.15537/smj.2018.4.21560. PMID: 29619496; PMCID: PMC5938658. |
| Task shifting in oral health care, broadening oral health care workforce, redistribution of tasks in workforce, workforce inoovations, dental therapists, community health workers, health support workers | 1. Negro KS, Scott JM, Marcenko M, Chi DL. Assessing the Feasibility of Oral Health Interventions Delivered by Social Workers to Children and Families in the Foster Care System. Pediatr Dent. 2019 Jan 15;41(1):48-51. PMID: 30803477; PMCID: PMC6392446. 2. Mertz E, Kottek A, Werts M, Langelier M, Surdu S, Moore J. Dental Therapists in the United States: Health Equity, Advancing. Med Care. 2021 Oct 1;59(Suppl 5):S441-S448. doi: 10.1097/MLR.0000000000001608. PMID: 34524241; PMCID: PMC8428854. 3. Mehta M, Erwin PC. Mid-level Practitioners in Oral Health: Tennessee Dental Professional's Attitudes and Perceptions of the Dental Therapist Workforce Model. J Health Care Poor Underserved. 2018;29(3):997-1010. doi: 10.1353/hpu.2018.0075. PMID: 30122679. 4. Mathu-Muju KR, Kong X, Brancato C, McLeod J, Bush HM. Utilization of community health workers in Canada's Children's Oral Health Initiative for indigenous communities. Community Dent Oral Epidemiol. 2018 Apr;46(2):185-193. doi: 10.1111/cdoe.12352. Epub 2017 Nov 23. PMID: 29168578. 5. Luo YL, Simon L, Leiviska K, Seyffer D, Friedland B. A survey of dental therapists' practice patterns and training in Minnesota. J Am Dent Assoc. 2021 Oct;152(10):813-821. doi: 10.1016/j.adaj.2021.05.004. Epub 2021 Aug 13. PMID: 34392938. 6. Hodgins F, Sherriff A, Gnich W, Ross AJ, Macpherson LMD. The effectiveness of Dental Health Support Workers at linking families with primary care dental practices: a population-wide data linkage cohort study. BMC Oral Health. 2018 Nov 21;18(1):191. doi: 10.1186/s12903-018-0650-z. PMID: 30463549; PMCID: PMC6249895. 7. Farmer J, Peressini S, Lawrence HP. Exploring the role of the dental hygienist in reducing oral health disparities in Canada: A qualitative study. Int J Dent Hyg. 2018 May;16(2):e1-e9. doi: 10.1111/idh.12276. Epub 2017 Mar 24. PMID: 28337843. 8. Crete P, Boyd LD, Fitzgerald JK, LaSpina LM. Access to Preventive Oral Health Services for Homebound Populations: A pilot program. J Dent Hyg. 2018 Dec;92(6):24-32. PMID: 30643001. 9. Catalanotto F, Hill LF. Dental Therapists' Impact on Access to Care and Oral Health Equity. Compend Contin Educ Dent. 2021 May;42(5):256-257. PMID: 33980024. 10. Catalanotto F. In Defense of Dental Therapy: An Evidence-Based Workforce Approach to Improving Access to Care. J Dent Educ. 2019 Feb;83(2 Suppl):S7-S15. doi: 10.21815/JDE.019.036. PMID: 30709933. 11. Amorim LP, Senna MIB, Paula JS, Rodrigues LG, Chiari APG, Ferreira RC. Oral health work process: disparity between teams in Brazil, 2014. Epidemiol Serv Saude. 2021 Feb 5;30(1):e2019533. English, Portuguese. doi: 10.1590/S1679-49742021000100013. PMID: 33566895. 12. Ahmad B, Landes D, Moffatt S. Dental Public Health In Action: Barriers to oral healthcare provision for older people in residential and nursing care homes: A mixed method evaluation and strategy development in County Durham, North East England. Community Dent Health. 2018 Aug 30;35(3):136-139. doi: 10.1922/CDH_4143Ahmad04. PMID: 30102021. |
| Wait times, timeliness, dental trauma | 1. Moran V, Suhrcke M, Ruiz-Castell M, Barré J, Huiart L. Investigating unmet need for healthcare using the European Health Interview Survey: a cross-sectional survey study of Luxembourg. BMJ Open. 2021 Aug 3;11(8):e048860. doi: 10.1136/bmjopen-2021-048860. PMID: 34344682; PMCID: PMC8336210. 2. Lima AMC, Garbin CAS, Corrente JE, Saliba NA, Moimaz SAS. Case-resolving capacity of dental care of the Unified Health System: the perception of users in a city in the state of São Paulo, Brazil. Cien Saude Colet. 2018 May;23(5):1657-1666. doi: 10.1590/1413-81232018235.17902016. PMID: 29768618. 3. Kayıllıoğlu Zencircioğlu Ö, Eden E, Öcek ZA. Access to health care after dental trauma in children: A quantitative and qualitative evaluation. Dent Traumatol. 2019 Jun;35(3):163-170. doi: 10.1111/edt.12467. Epub 2019 Mar 18. PMID: 30758139. 4. Cavalcanti RP, da Silva RO, Martelli PJL, Sobrinho JEL, Pucca Júnior GA, da Silveira Gaspar G, de Lucena EHG. Factors associated with the waiting time for access to specialized oral healthcare services in Brazil. Community Dent Oral Epidemiol. 2022 Feb;50(1):58-66. doi: 10.1111/cdoe.12720. PMID: 34967971. 5. Alonaizan FA, Almas K, Nazir MA, Almazrou D, Alzamil M, AlOlyani MA. Medical Conditions, Oral Health Practices, and Barriers to Treatment among Patients Visiting a Teaching Dental Hospital in Eastern Saudi Arabia. ScientificWorldJournal. 2022 Feb 4;2022:4495757. doi: 10.1155/2022/4495757. PMID: 35153627; PMCID: PMC8837447. |
| Age and/or gender differences, demographic disparities | 1. Massoni ACLT, Porto É, Ferreira LRBO, Silva HP, Gomes MDNC, Perazzo MF, D'avila S, Granville-Garcia AF. Access to oral healthcare services of adolescents of a large-size municipality in northeastern Brazil. Braz Oral Res. 2020 Mar 27;34:e029. doi: 10.1590/1807-3107bor-2020.vol34.0029. PMID: 32236318. 2. Jang JH, Kim JL, Kim JH. Associations between Dental Checkups and Unmet Dental Care Needs: An Examination of Cross-Sectional Data from the Seventh Korea National Health and Nutrition Examination Survey (2016-2018). Int J Environ Res Public Health. 2021 Apr 3;18(7):3750. doi: 10.3390/ijerph18073750. PMID: 33916791; PMCID: PMC8038362. 3. Gupta A, Feldman S, Perkins RB, Stokes A, Sankar V, Villa A. Predictors of dental care use, unmet dental care need, and barriers to unmet need among women: results from NHANES, 2011 to 2016. J Public Health Dent. 2019 Dec;79(4):324-333. doi: 10.1111/jphd.12334. Epub 2019 Aug 13. PMID: 31407356. 4. Gonzales G, Dedania R, Driscoll R. Health Insurance Coverage and Access to Care Among US-Born and Foreign-Born Sexual Minorities. J Immigr Minor Health. 2019 Jun;21(3):540-548. doi: 10.1007/s10903-018-0774-x. PMID: 29946865. 5. Durey A, Lee SK, Adebayo B, Slack-Smith L. Building oral health capacity in a women's health service. Aust J Prim Health. 2018 Nov;24(5):417-421. doi: 10.1071/PY17102. PMID: 30012241. 6. Choi SE, Kalenderian E, Normand SL. Measuring the quality of dental care among privately insured children in the United States. Health Serv Res. 2022 Feb;57(1):137-144. doi: 10.1111/1475-6773.13713. Epub 2021 Jul 29. PMID: 34327703; PMCID: PMC8763286. 7. Reda SF, Reda SM, Thomson WM, Schwendicke F. Inequality in Utilization of Dental Services: A Systematic Review and Meta-analysis. Am J Public Health. 2018 Feb;108(2):e1-e7. doi: 10.2105/AJPH.2017.304180. Epub 2017 Dec 21. PMID: 29267052; PMCID: PMC5846590. |
| Lack of time of citizens | 1. Mariño R, Glenister K, Bourke L, Morgan M, Atala-Acevedo C, Simmons D. Patterns of use of oral health care services in Australian rural adults: the Crossroads-II Dental sub-study. Aust Dent J. 2021 Dec;66(4):397-405. doi: 10.1111/adj.12865. Epub 2021 Jul 10. PMID: 34152019. 2. Bahramian H, Mohebbi SZ, Khami MR, Quinonez RB. Qualitative exploration of barriers and facilitators of dental service utilization of pregnant women: A triangulation approach. BMC Pregnancy Childbirth. 2018 May 10;18(1):153. doi: 10.1186/s12884-018-1773-6. PMID: 29747592; PMCID: PMC5946421. |
| Appointment setting, unscheduled care, emergency care | 1. Guivarc'h M, Saliba-Serre B, Le Coz P, Bukiet F. A cross-sectional analysis of patient care pathways and profiles in a dental emergency department. Int Dent J. 2020 Feb;70(1):21-28. doi: 10.1111/idj.12516. Epub 2019 Aug 29. PMID: 31468531; PMCID: PMC9379167. 2. Grossman A, Kim NY, Stagnell S, Allen Y, Shah S. Assessment of public and professional perceptions of access to unscheduled dental care. Br Dent J. 2018 May 25;224(10):815-820. doi: 10.1038/sj.bdj.2018.357. Erratum in: Br Dent J. 2018 Jun 22;224(12):975. PMID: 29795485. |
| Dental care education and pipeline programs | 1. Greenway RA, Scott JM, Loveless EC, Bigham RR, Simmer-Beck ML. Evaluation of a pipeline program at strengthening applications, increasing diversity, and increasing access to care. J Dent Educ. 2021 May;85(5):642-651. doi: 10.1002/jdd.12508. Epub 2020 Dec 17. PMID: 33332594. 2. Byrappagari D, Jung Y, Chen K. Oral health care for patients with developmental disabilities: A survey of Michigan general dentists. Spec Care Dentist. 2018 Sep;38(5):281-290. doi: 10.1111/scd.12303. Epub 2018 Jun 26. PMID: 29944189. |
| Access defined as a quantification of number of appointments | 1. França MASA, Freire MDCM, Pereira EM, Marcelo VC. Indicators proposed by the Brazilian Ministry of Health for monitoring and evaluating oral health actions in the National Health System: documentary research, 2000-2017. Epidemiol Serv Saude. 2020 Feb 17;29(1):e2018406. English, Portuguese. doi: 10.5123/S1679-49742020000100002. PMID: 32074194. |
| Health literacy | 1. Firmino RT, Fraiz FC, Montes GR, Paiva SM, Granville-Garcia AF, Ferreira FM. Impact of oral health literacy on self-reported missing data in epidemiological research. Community Dent Oral Epidemiol. 2018 Dec;46(6):624-630. doi: 10.1111/cdoe.12415. Epub 2018 Aug 24. PMID: 30144146. 2. Baliga SM. Child oral health-care literacy in India: Can access to services be improved? J Indian Soc Pedod Prev Dent. 2019 Jan-Mar;37(1):1-2. doi: 10.4103/JISPPD.JISPPD_18_19. PMID: 30804299. 3. Bahramian H, Mohebbi SZ, Khami MR, Quinonez RB. Qualitative exploration of barriers and facilitators of dental service utilization of pregnant women: A triangulation approach. BMC Pregnancy Childbirth. 2018 May 10;18(1):153. doi: 10.1186/s12884-018-1773-6. PMID: 29747592; PMCID: PMC5946421. |
| Caregiver status, intergenerational effects, family setting | 1. Finlayson TL, Chuang E, Baek JD, Seidman R. Dental Service Utilization Among Children in the Child Welfare System. Matern Child Health J. 2018 May;22(5):753-761. doi: 10.1007/s10995-018-2444-y. PMID: 29423585; PMCID: PMC5895518. 2. Finlayson TL, Asgari P, Dougherty E, Tadese BK, Stamm N, Nunez-Alvarez A. Child, caregiver, and family factors associated with child dental utilization among Mexican migrant families in California. Community Dent Health. 2018 May 30;35(2):89-94. doi: 10.1922/CDH_4201Finlayson06. PMID: 29645407. 3. Edelstein BL, Rubin MS, Clouston SAP, Reusch C. Children's dental service use reflects their parents' dental service experience and insurance. J Am Dent Assoc. 2020 Dec;151(12):935-943. doi: 10.1016/j.adaj.2020.08.018. PMID: 33228886. 4. Como DH, Stein Duker LI, Polido JC, Cermak SA. The Persistence of Oral Health Disparities for African American Children: A Scoping Review. Int J Environ Res Public Health. 2019 Feb 27;16(5):710. doi: 10.3390/ijerph16050710. PMID: 30818846; PMCID: PMC6427601. 5. Comassetto MO, Baumgarten A, Kindlein KA, Hilgert JB, Figueiredo MC, Faustino-Silva DD. Access to oral health in early childhood in the city of Porto Alegre, Brazil. Cien Saude Colet. 2019 Mar;24(3):953-961. Portuguese, English. doi: 10.1590/1413-81232018243.29082016. PMID: 30892516. 6. Carrellas A, Day A, Cadet T. Oral Health Care Needs of Young Adults Transitioning from Foster Care. Health Soc Work. 2018 Feb 1;43(1):22-29. doi: 10.1093/hsw/hlx044. PMID: 29190341. 7. Blackburn J, Bennett A, Fifolt M, Rucks AC, Taylor H, Wolff P, Sen B. Pediatric dental care use and parent- or caregiver-rated oral health among Alabama children enrolled in Medicaid. J Am Dent Assoc. 2020 Jun;151(6):416-426. doi: 10.1016/j.adaj.2020.02.016. PMID: 32450980. |
| Pediatric oral health care | 1. Edelstein BL. Pediatric Oral Health Policy: Its Genesis, Domains, and Impacts. Pediatr Clin North Am. 2018 Oct;65(5):1085-1096. doi: 10.1016/j.pcl.2018.05.012. PMID: 30213351. 2. Curry S, Serban N. Accounting for uncertainty in policy decision making: Improving access to pediatric dental care. Health Serv Res. 2021 Apr;56(2):214-224. doi: 10.1111/1475-6773.13618. Epub 2021 Jan 22. PMID: 33481258; PMCID: PMC8186474. 3. Crall JJ, Vujicic M. Children's Oral Health: Progress, Policy Development, And Priorities For Continued Improvement. Health Aff (Millwood). 2020 Oct;39(10):1762-1769. doi: 10.1377/hlthaff.2020.00799. PMID: 33017249. 4. Como DH, Stein Duker LI, Polido JC, Cermak SA. The Persistence of Oral Health Disparities for African American Children: A Scoping Review. Int J Environ Res Public Health. 2019 Feb 27;16(5):710. doi: 10.3390/ijerph16050710. PMID: 30818846; PMCID: PMC6427601. 5. Chalmers NI, Wislar JS, Hall M, Thurm C, Ng MW. Trends in Pediatric Dental Care Use. Dent Clin North Am. 2018 Apr;62(2):295-317.e12. doi: 10.1016/j.cden.2017.11.008. PMID: 29478459. 6. Blackburn J, Bennett A, Fifolt M, Rucks AC, Taylor H, Wolff P, Sen B. Pediatric dental care use and parent- or caregiver-rated oral health among Alabama children enrolled in Medicaid. J Am Dent Assoc. 2020 Jun;151(6):416-426. doi: 10.1016/j.adaj.2020.02.016. PMID: 32450980. 7. Adjaye-Gbewonyo D, Black LI. Dental Care Utilization Among Children Aged 1-17 Years: United States, 2019 and 2020. NCHS Data Brief. 2021 Dec;(424):1-8. PMID: 34860646. |
| Worse oral health/ perceived oral health | 1. Echeverria MS, Silva AER, Agostini BA, Schuch HS, Demarco FF. Regular use of dental services among university students in southern Brazil. Rev Saude Publica. 2020;54:85. doi: 10.11606/s1518-8787.2020054001935. Epub 2020 Aug 21. PMID: 32844983; PMCID: PMC7438030. 2. Carreiro DL, Souza JGS, Coutinho WLM, Haikal DS, Martins AMEBL. Acesso aos serviços odontológicos e fatores associados: estudo populacional domiciliar [Access to dental services and related factors: a home-based population study]. Cien Saude Colet. 2019 Mar;24(3):1021-1032. Portuguese. doi: 10.1590/1413-81232018243.04272017. PMID: 30892522. |
| Women’s health and pregnancy | 1. de Fátima Vasques Monteiro M, Barbosa CP, Vertamatti MAF, Tavares MNA, de Oliveira Carvalho AC, Alencar APA. Access to public health services and integral care for women during the puerperal gravid period period in Ceará, Brazil. BMC Health Serv Res. 2019 Nov 20;19(1):851. doi: 10.1186/s12913-019-4566-3. PMID: 31747914; PMCID: PMC6868722. 2. Chenwi HF, Savitz DA. Distribution of Preventive Dental Care during Pregnancy in Rhode Island, 2012 to 2015. R I Med J (2013). 2018 Nov 1;101(9):19-22. PMID: 30384514. 3. Bahramian H, Mohebbi SZ, Khami MR, Quinonez RB. Qualitative exploration of barriers and facilitators of dental service utilization of pregnant women: A triangulation approach. BMC Pregnancy Childbirth. 2018 May 10;18(1):153. doi: 10.1186/s12884-018-1773-6. PMID: 29747592; PMCID: PMC5946421. |
| HIV and oral care | 1. Dandachi D, May SB, Davila JA, Cully J, Amico KR, Kallen MA, Giordano TP. The Association of Unmet Needs With Subsequent Retention in Care and HIV Suppression Among Hospitalized Patients With HIV Who Are Out of Care. J Acquir Immune Defic Syndr. 2019 Jan 1;80(1):64-72. doi: 10.1097/QAI.0000000000001874. PMID: 30272637; PMCID: PMC6289860. |
| Employment status | 1. Choi JW, Choi Y, Lee TH, Lee HJ, Ju YJ, Park EC. Employment status and unmet dental care needs in South Korea: a population-based panel study. BMJ Open. 2019 Mar 30;9(3):e022436. doi: 10.1136/bmjopen-2018-022436. PMID: 30928921; PMCID: PMC6475345. |
| Substance use disorders | 1. Carlsen SL, Isaksen K, Fadnes LT, Lygren OJS, Åstrøm AN. Non-financial barriers in oral health care: a qualitative study of patients receiving opioid maintenance treatment and professionals' experiences. Subst Abuse Treat Prev Policy. 2021 May 17;16(1):44. doi: 10.1186/s13011-021-00379-6. PMID: 34001202; PMCID: PMC8130131. |
| Office hours | 1. Amorim LP, Senna MIB, Alencar GP, Rodrigues LG, de Paula JS, Ferreira RC. User satisfaction with public oral health services in the Brazilian Unified Health System. BMC Oral Health. 2019 Jun 25;19(1):126. doi: 10.1186/s12903-019-0803-8. Erratum in: BMC Oral Health. 2019 Oct 15;19(1):221. PMID: 31238976; PMCID: PMC6593529. |
| Regional organization and models of public provision | 1. Almeida PF, Giovanella L, Martins Filho MT, Lima LD. Regionalized networks and guarantee of specialized health care: the experience of Ceará, Brazil. Cien Saude Colet. 2019 Dec;24(12):4527-4540. Portuguese, English. doi: 10.1590/1413-812320182412.25562019. Epub 2019 Aug 30. PMID: 31778503. |
| Medical tourism and equity concerns | 1. Adams K, Snyder J, Crooks VA, Berry NS. A critical examination of empowerment discourse in medical tourism: the case of the dental tourism industry in Los Algodones, Mexico. Global Health. 2018 Jul 20;14(1):70. doi: 10.1186/s12992-018-0392-3. PMID: 30029610; PMCID: PMC6054732. |

**Annex 3: Voting results sensitivity checks**

We checked the results of the online voting to avoid introducing skewed results based on higher participant numbers of one stakeholder group or one country by equally weighting all countries or stakeholder groups. As there are no major differences apparent, we decided to go with the overall mean due to relatively low participant numbers overall.

**Annex 4: Detailed list of non-researcher participants by country and stakeholder group**

|  | patients | providers | policymaker | vulnerable |
| --- | --- | --- | --- | --- |
| Denmark | 1 | 1 | 2 | 1 |
| Sweden | - | 2 | - | 1 |
| UK | - | 1 | 1 | - |
| the Netherlands | - | 3 | - | - |
| Germany | 1 | 1 | 1 | 1 |
| Malta | 1 | 1 | - | - |
| Portugal | 3 | 1 | 1 | - |

**Annex 5: starting list derived from the literature screening and used as supporting material in workshops**

# Patient safety

| **Possible topics deduced from literature** | **subitems** |
| --- | --- |
| Avoid, mitigate, or minimize adverse events (from previous definition) |  |
| Avoid or minimize medical errors and treatment errors (this includes over- and undertreatment) (from previous definition) |  |
| Advocate a blame-free culture to facilitate quality improvement (from previous definition) |  |
| Learn from safety incidents to improve the quality of care (from previous definition) |  |
| Enact minimum safety standards (from previous definition) |  |
| Oral health literacy | Community engagement |
| Data management | Privacy, secruirity, Integrated electronic health record |
| Tailored care | Risk assessment to measure disease, medically complex patients, Cultural safety,  Medical history,  Integrated electronic health record |
| Adverse events | Trigger tools, Unintended harm,  Near misses, Medical error, patient safety incidents, Strategies for overcoming failure,  Terminology |
| Reporting systems | Inspection (method / framework),  Patient engagement to improve safety |
| hazardous healthcare  conditions | Safety checklist, Inspection (method / framework) |
| Trusting dentist-patient relationship |  |
| Medication use | Prescribing medicine, Medical interaction |
| Standardized procedures | Clinical audit, guidelines for treatment, diagnostic codes, Continued education,  Radiation control,  Evidence-based dentistry,  Medical emergencies |
| Effective communication | language barrier |
| Pandemic preparedness | Covid-19  (guidlines, PPE, testing, infrastructure adaption, patient care management) |
| Interprofessional coordination | Working together, communication |
| Provider safety | Ocupational & instrumental safety, mental health |
| Continuity of care |  |
| Patient mangement | Urgent appointments, Triage |
| Enabeling factors for patients to seek care | Work environment, especially time-off to go to dentist |

# Effectiveness

| **Possible topics deduced from literature** | **subitems** |
| --- | --- |
| Be informed by the most recent available scientific evidence and guidelines (from previous definition) |  |
| Translate scientific evidence into clinical practice (from previous definition) |  |
| Maximize the intended health outcome of care (from previous definition) |  |
| Be provided to the patients who benefit from the care, and aspire to minimize harm (from previous definition) |  |
| Provide the best available care for the needs and preferences of each patient (from previous definition) |  |
| Evidence based care |  |
| Tailored care | Care dependent patients |
| Managing available resources | Time, number of dental visits |
| Symptom relief | Kinesiophobia, pain, maximum mouth opening, depression, psychosocial impacts, function, Sleep (quality), Activity interference |
| Quality of life |  |
| Patient reporting | PROMS, Function, sensation, Patient satisfaction / preference |
| Anxiety, dental fear |  |
| Support for self-management | (oral hygiene) self-efficacy, oral hygiene / health knowledge -> education, oral health behavior, diet, Patient cooperation / compliance, (Electronic) health literacy |
| prevention of infectious diseases | Pneumonia (ICU), |
| Management of disease | risk assessment, prevalence, prevalence of untreated disease, need for medication, Need for further interventions / maintenance / restoration, Not failing / success / failure rate, referral, Treatment planning, Quality of dentures, cleaning dentures |
| Patient assessment & diagnosis | Signs of illness (swelling, tenderness, radiographic evidence), need for medication, Function, sensation, sensitivity, specificity, Screening |
| Adverse events |  |
| Provider adherence to guidelines / standards of care |  |
| Clinical skill of provider |  |

# Patient-centeredness

| **Possible topics deduced from literature** | **subitems** |
| --- | --- |
| Be respectful of and responsive to individual patient preferences, needs, values, fears, concerns, and/or cultures (from previous definition) |  |
| Follow a shared decision-making model when making clinical decisions. To gain a patient’s trust, the oral healthcare  professional should communicate with and listen to the patient, then informing, educating, and guiding the patient to  ensure that patient values shape all clinical decisions (from previous definition) |  |
| Feedback from patients |  |
| Oral health literacy |  |
| Routine feedback |  |
| Expectations and views of patients |  |
| Communication with patients |  |
| Ambiance |  |
| Patient involvement decision making |  |
| Patient satisfaction |  |
| Interprofessional care |  |
| Motivational interviewing |  |
| Respectfulness |  |
| Involvement in treatment planning/shared decision making |  |
| Patient values |  |
| Informing the patient |  |
| Patient needs/desires |  |
| Tailored care |  |
| Patient perception of disease and treatment (1. pressure: physical, psychosocial, financial; 2. coping adaptation; 3.reflection & evaluation) |  |
| Patient feelings, emotional / psychological support, supportive environment |  |
| Use of PROMs & PREMs  (e.g. OHRQoL, satisfaction) also relevant for research to be more useful, the value of outcomes to patients should be checked in order to effectively drive quality improvement |  |
| Patient activation |  |
| Dental workforce – patient relationship, trust |  |
| No blame culture |  |
| Cultural competence |  |
| Raising awareness, involving family, friends, community |  |
| Access to personal health data, patient-health-portals |  |
| (recognition of) Oral manifestations of disease and disease related impairments (also psychosocial) |  |
| Seeing patient as a whole, person-focused care, (also: special needs: chronic disease, mental health, behavioral, physical, social) |  |
| Good availability of services (e.g. opening hours), accommodating |  |
| Dental / health registry (e.g. caries risk assessment) |  |
| Community connection (e.g. connects patients with needed community & social services) |  |
| Physical & socioeconomic assessment |  |
| Person-/ patient-centered health system design (supported by values, payment environments, encouraging personal health ownership, respects patients’ dignity, adaptable to input from person/patient & provider) | . |
| Interoperability of general & dental electronic health records |  |
| Involving key stakeholders for planning of patient-centered research |  |
| Financial hardship screening, cost conversations between dental team & patient |  |

# Timeliness

| **Possible topics deduced from literature** | **subitems** |
| --- | --- |
| Avoid unnecessary delays in access and utilization of care (from previous definition) |  |
| Prioritize care based on need, not on demand (e.g., employ checkup and recall intervals based on patient’s own risks). (from previous definition) |  |
| Implement care coordination between healthcare providers and institutions. (from previous definition) |  |
| Prioritize prevention; avoid too early use of restorative and other treatments. (from previous definition) |  |
| Implement school-based oral healthcare if appropriate (from previous definition) |  |
| Timely follow up after treatment |  |
| Timely dental care |  |
| Harmful waiting times/protracted waiting times |  |
| Absenteeism from work due to complications of  waiting times |  |
| Influence affordability of care on timeliness (or combine with access to care) |  |
| Availability of dental professionals |  |
| Timeliness of guidelines |  |
| Timeliness medical prescriptions |  |
| Timely advice about oral care |  |
| Timeliness in access to care |  |
| Timely access to care through health literacy |  |
| Timely access to care through screening |  |
| Demographic, socioeconomic, environmental determinants of timely access |  |
| Interprofessional care, effective & timely referral pathways, knowledge of oral health in other healthcare workers, comprehensive care to reduce unmet health needs, continuity of care |  |
| Covid-19, pandemic considerations of timely access |  |
| Timeliness of data (collection) (quality improvement, research, etc.), (implant) registry (data concerning patient, implant, complications, follow-up) to facilitate further treatments |  |
| Legal framework, regulatory barriers to receive timely care |  |
| Willingness of providers to provide care to special needs patients due to perceived lack of knowledge/training |  |
| Triaging patients |  |
| Perceived barriers (special needs patients) ->  organization & resources, financial & welfare policies, empathy & accommodation of staff | . |
| Timely information to patient (regarding treatment) |  |

# Efficiency

| **Possible topics deduced from literature** | **Subitems** |
| --- | --- |
| Avoid waste of resources, including waste of human resources, time, equipment, dental materials. Such measures will  also minimize the carbon footprint of oral healthcare (from previous definition) |  |
| Prioritize cost-efficient interventions that provide good value-for-money (from previous definition) |  |
| Encourage prevention and discourage expensive restorative care (from previous definition) |  |
| Rely on patients’ oral health needs as the central basis for resource and workforce planning (from previous definition) |  |
| Form an integral part of medicine and discourage the dental-medical divide (from previous definition) |  |
| Waste of time |  |
| Waste of resources |  |
| Productivity |  |
| Waste of materials/supplies/equipment |  |
| Technical efficiency |  |
| Efficiency of the workforce (role substitution) |  |
| Usability |  |
| Understanding enablers and barriers to efficiency and acting upon this understanding |  |
| Integration of primary and oral health care, interprofessional working, sharing information |  |
| Building connections, partnerships with the community (“promote the sharing of information, facilitate provision of basic diagnostic services, and bring the bidirectional flow of knowledge, training, and skills to one another in a systematic and sustained manner”) |  |
| net revenue, no-show rates, total encounters, and number of encounters for with special patient groups (such as young children and pregnant women) |  |
| Survival time = efficiency (i.e. sustainability, etc.?) |  |
| Efficient data collection, storage & use (e.g. big data) |  |
| Oral health problems as a reason for absenteeism and presenteeism (-> how does this relate to timely care? -> workers go to work with oral health problems and don’t receive care -> care is not timely?) |  |
| Education |  |

# Equitability

| **Possible topics deduced from literature** | **subitems** |
| --- | --- |
| Not vary in quality and availability because of gender, ethnicity, cultural background, religion and belief, geographic  location, and/or socioeconomic status (from previous definition) |  |
| Provide equitable access to all |  |
| Not be compromised in terms of either quality or availability on the basis of a patient’s health status (from previous definition) |  |
| Address inequities in oral health service design, planning, and commissioning (from previous definition) |  |
| Incorporate equitability in the design of policy and clinical practice guidelines(from previous definition) |  |
| ethnicity disparities |  |
| Access to care: costs |  |
| Geographical disparities |  |
| Socio-economic inequality |  |
| Health literacy |  |
| Cultural disparities/cultural competence |  |
| Inequality due to education |  |
| Barriers to seek oral care |  |
| Interprofessional care to reduce disparities |  |
| Disability and medical related disparities |  |
| Disparity due to age |  |
| Insurance  (e.g. Medicaid vs. private,  China: urban vs rural vs no insurance) / safety net |  |
| “Migration vs. native”, also migration history  (Also barriers due to lacking provider training for migrant patients, language barriers) |  |
| Socioeconomic status of children  / their family, children living conditions (e.g. maternal / parental education, care giver: parents, grandparents, etc., presence of caregiver)  Values and attitudes of parents  Caregiver involvement in brushing,  Parents oral health practices, parents alcohol consumption,  Childhood obesity |  |
| Disparities in community connection, community connection to overcome disparities, community health programs |  |
| Vulnerable groups (e.g. exposure to intimate partner violence) |  |
| Importance of research regarding inequalities / inequity, research design (i.e. what measures are used for example to define SES) |  |
| Equitable policies, funding |  |
| Gender / sex |  |
| Institutionalized living (e.g. size of facility as a factor influencing oral health) |  |
| Diet (e.g. as a part of a systems approach to socioeconomic oral health inequities i.e. socioeconomic conditions and nutrition) |  |
| Screening reduces disparities |  |
| Disparities in safe water / fluoridated water / public health measures  Disparity reduction through water fluoridation, sealant prevalence |  |
| Social isolation  Social support  Also help for people formerly living institutionalized (transition, health care access afterwards) |  |
| Health care provision models (e.g. private / public providers) |  |
| Marital status |  |
| Having been to a care provider before (lifetime perspective) |  |
| Provider variation (individual variation, workforce type variation) |  |
| Discrimination (as a barrier to receive preventive care; reasons for discrimination: lack of money, social class and type of working occupation) |  |
| Equity in covid-19 restrictions (or absence of it) |  |
| Employment (status) |  |
| Patient-provider relation (barriers) |  |
| Health system barriers for special needs patients (e.g. provider training, logistics, equipment) |  |
| Social inequity due to oral health problems |  |
| Substance use (alcohol, nicotine) |  |
| Not receiving health advice due to socioeconomic factors |  |

# Accessibility

| **Possible topics deduced from literature** | **subitems** |
| --- | --- |
| Be accessible and available, in a timely fashion, to everyone seeking care  (from previous definition) |  |
| Be independent of personal characteristics, such as gender, ethnicity, cultural background, socioeconomic status,  religion and belief, geographic location, and/or the patient’s health status (from previous definition) |  |
| Be determined by appointment times and recall intervals that are based on patient needs and preferences (from previous definition) |  |
| Limitations in supply-side resources and availability of dentists |  |
| Inequalities based on resident status (e.g. refugees, migrants) as well as language barriers and lack of knowledge among these groups |  |
| Disparities/challenges for patients with special needs, including disabilities, chronic diseases (also elderly), difficulties in accessing care due to disease |  |
| Integrated oral health care (e.g. integration between primary and oral care, between oral care and long term care faciliites), care coordination |  |
| Affordability of care and out of pocket spending, population (or insurance) coverage to improve financial access |  |
| Socioeconomic status and resulting disparities |  |
| Disparities based on geographical location, distance to facilities, transportation problems (geographical access) |  |
| Disparities based on ethnicities/cultural background and lack of awareness/knowledge among certain populations |  |
| Discrimination (LGBTQ+, racism) |  |
| Fear |  |
| Ability to have paid leave vs. unpaid leave |  |
| Big data and technological innovations as part of oral health care transformation |  |
| Perceptions of need for oral health care |  |
| Suboptimal patient experiences and satisfaction with dentists, trust |  |
| Network ties of citizens, social participation |  |
| Remote oral health care, teledentistry, domiciliary care |  |
| Unmet need among patients with mental illness |  |
| Task shifting in oral health care, broadening oral health care workforce, redistribution of tasks in workforce, workforce inoovations, dental therapists, community health workers, health support workers |  |
| Wait times, timeliness, dental trauma |  |
| Age and/or gender differences, demographic disparities |  |
| Lack of time of citizens |  |
| Appointment setting, unscheduled care, emergency care |  |
| Dental care education and pipeline programs |  |
| Access defined as a quantification of number of appointments |  |
| Health literacy |  |
| Caregiver status, intergenerational effects, family setting |  |
| Pediatric oral health care |  |
| Worse oral health/ perceived oral health |  |
| Women’s health and pregnancy |  |
| HIV and oral care |  |
| Employment status |  |
| Substance use disorders |  |
| Office hours |  |
| Regional organization and models of public provision |  |
| Medical tourism and equity concerns |  |
